# Picuris Pueblo oral history and genomics reveal continuity in US Southwest

**DOI:** 10.1038/s41586-025-08791-9

**Published:** 2025-04-30

**Authors:** Thomaz Pinotti, Michael A. Adler, Richard Mermejo, Julie Bitz-Thorsen, Hugh McColl, Gabriele Scorrano, Motahareh Feizabadifarahani, Devlin Gandy, Matthew Boulanger, Charleen Gaunitz, Jesper Stenderup, Abigail Ramsøe, Thorfinn Korneliussen, Fabrice Demeter, Fabrício R. Santos, Lasse Vinner, Martin Sikora, David J. Meltzer, J. Víctor Moreno-Mayar, Craig Quanchello, Eske Willerslev

**Affiliations:** 1 Lundbeck Foundation GeoGenetics Centre, GLOBE Institute, University of Copenhagen, Copenhagen, Denmark; 2 Laboratório de Biodiversidade e Evolução Molecular (LBEM), Instituto de Ciências Biológicas, Universidade Federal de Minas Gerais, Belo Horizonte, Brazil; 3 Department of Anthropology, Southern Methodist University, Dallas, TX USA; 4 Picuris Pueblo, Peñasco, NM USA; 5 Center for Molecular Anthropology for the Study of Ancient DNA, Department of Biology, University of Rome ‘Tor Vergata’, Rome, Italy; 6 Department of Archaeology and Classical Studies, Stockholm University, Stockholm, Sweden; 7 Department of Archaeology, University of Cambridge, Cambridge, UK; 8 Eco-anthropologie (EA), Departement ABBA, Muséum national d’Histoire naturelle, CNRS, Université Paris Cité, Musée de l’Homme, Paris, France; 9 Department of Genetics, University of Cambridge, Cambridge, UK; 10 MARUM Center for Marine Environmental Sciences, University of Bremen, Bremen, Germany

**Keywords:** Population genetics, Genomics, Archaeology, Anthropology

## Abstract

Indigenous groups often encounter significant challenges when asserting ancestral claims and cultural affiliations based on oral histories, particularly in the USA where such narratives have historically been undervalued. Although ancient DNA offers a tool to complement traditional knowledge and address gaps in oral history, longstanding disregard for Indigenous sovereignty and beliefs has understandably led many Indigenous communities to distrust DNA studies^1–4^. Earlier research often focused on repatriation claims^5–7^, whereas more recent work has increasingly moved towards enhancing Tribal histories^8,9^. Here we present a collaborative study initiated by a federally recognized Native American tribe, the sovereign nation of Picuris Pueblo in the Northern Rio Grande region of New Mexico, USA, to address gaps in traditional knowledge and further their understanding of their population history and ancestry. We generated genomes from 16 ancient Picuris individuals and 13 present-day members of Picuris Pueblo, providing genomic data spanning the last millennium. We show genetic continuity between ancient and present-day Picuris, and more broadly with Ancestral Puebloans from Pueblo Bonito in Chaco Canyon^10^, 275 km to the west. This suggests a firm spatiotemporal link among these Puebloan populations of the North American Southwest. Furthermore, we see no evidence of population decline before European arrival^11–13^, and no Athabascan ancestry in individuals predating 1500 ce, challenging earlier migration hypotheses^14–16^. This work prioritizes Indigenous control of genetic data and brings together oral tradition, archaeology, ethnography and genetics.

## Abstract (web summary)

A collaborative study initiated by the sovereign nation of Picuris Pueblo in the Northern Rio Grande region of New Mexico addresses gaps in traditional knowledge and furthers understanding of their population history and ancestry.

## Main

**Fig. 1:**
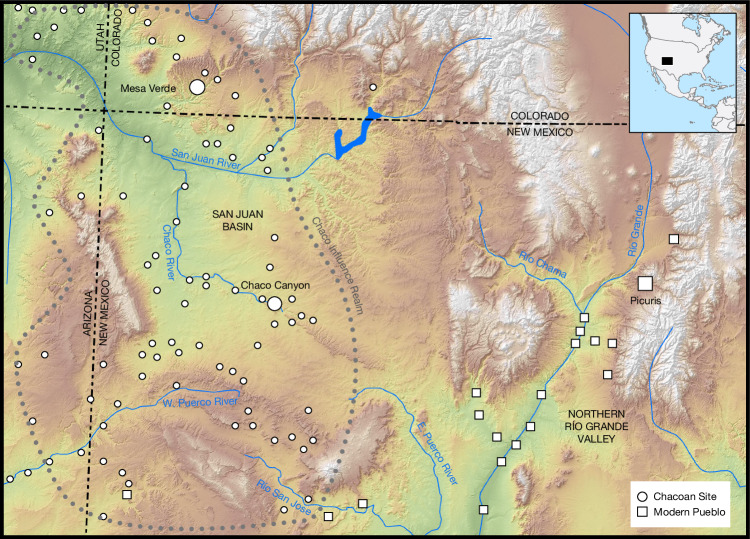
Map of the Four Corners region of the US Southwest, naming sites and rivers discussed in the text. Chaco culture (Ancestral Puebloan) related sites are indicated with circles and present-day Pueblos are indicated with squares. Base elevation data are derived from the USGS EROS SRTM 1-arc second digital elevation model^99^, with smoothing applied to produce derivative hillshade and terrain-relief datasets.

Ancient pueblos created by the Ancestral Pueblo culture, a term indicative of their ancestral relationship to contemporary Native American Pueblo Tribes, are among the most recognizable archaeological culture of North America. Many ancient pueblos have become the focus of US National Parks, Historic Parks or Monuments, and several have become UNESCO World Heritage Sites, including the large, multi-story masonry ‘Great Houses’ concentrated in Chaco Canyon within the San Juan River Basin on the Colorado Plateau in Northwestern New Mexico in the USA^17^. Chacoan Great Houses were constructed between roughly 850 and 1150 ce, and were central to a broader ritual landscape of agricultural communities across the northern Southwest (Fig. 1)^18,19^. Chaco Canyon was the centre of an extensive political and social network of more than 200 Great House communities covering an area larger than modern England. However, after around 1150 ce major construction ceased in Chaco Canyon and the area was largely depopulated for reasons still debated, which include drought, overexploitation of available ecological resources or arable land, collapse of regional exchange and belief systems or a combination of those factors^20,21^.

Where Chacoan populations resettled in roughly 1150 ce, and what subsequent migrations they might have undertaken, remains a debated topic within Southwestern Archaeology and Indigenous communities alike. One area that has gained significant interest for its connections to Chaco is the Northern Rio Grande (NRG) region. Dating to roughly 900 ce, the earliest NRG Puebloan centres were contemporaneous with early Chaco, whereas archaeological, historic and modern pueblos—such as Picuris—provide evidence of NRG cultural continuity since the depopulation of Chaco. Whereas the oral traditions of present-day Puebloans^22–30^ and many lines of scholarly evidence^18,31–33^ support some level of relationship between the inhabitants of Chaco Canyon and the NRG, the nature and depth of the relationship between these two distant regions is debated (see Supplementary Information 3.5 for a discussion on some archaeological models). This has inevitably led to scepticism about the strength of links between past and present Puebloan groups, which has negatively affected Tribal Nations seeking to protect and preserve Chaco Canyon and its ancestral role in the Puebloan world^11–13^.

Traditional knowledge keepers at Picuris Pueblo assert ancestral and cultural connections to numerous Ancestral Pueblo and Chaco culture sites in the San Juan Basin. The specifics of these relationships cannot be discussed outside traditional Picuris contexts, but include architectural motifs, an overlap between their racetracks with the system of Chacoan roads—which connect present-day Picuris with the San Juan Basin—and shared religious symbols found in material culture (such as pottery and sacred figurines) (Supplementary Information 3.1).

However, they also acknowledge uncertainties because of gaps in their oral history. Such gaps are inevitable in a centuries-old community that by our estimate (below) lost at least 85% of their members in the first several decades after European Contact. By 1700 ce, the community had shrunk to around 300 members, making them the smallest of the extant Pueblo nations. In addition, centuries of oppression and prohibition of Picuris religion and rituals, the loss of access to traditional and sacred landscapes, and the placement of Picuris children into Native American boarding schools inhibited the intergenerational transmission of cultural knowledge and history at Picuris. Cumulatively, these factors have often minimized Picuris’ voice in continuing discussions regarding the status and future of Chaco Canyon. The Picuris community initiated this research to bridge and mend gaps in their traditional knowledge with appropriate scientific data, and to address the extent to which they might have ancestral ties to Chaco Canyon and the regional system it anchored centuries ago.

Recognizing the potential of ancient DNA to help secure links to their past, the Governor and leadership of Picuris Pueblo requested some of their archaeological collaborators help them initiate conversations with the Centre for GeoGenetics (University of Copenhagen). In many consultations over a 2-year period, a mutually agreed-on collaborative research design was developed to investigate Picuris population, genomic and health history (Supplementary Information 2). A memorandum of agreement resulted between the sovereign nation of Picuris Pueblo and the University of Copenhagen for this collaborative study. Following that, Picuris approved the transfer of ancient individuals excavated from the pueblo in the 1960s^34^, that were on long-term loan to Southern Methodist University. For clarity, we have chosen to address both the Picuris Pueblo Tribal Nation and its members in the third person, even though some of its members are coauthors of this study.

## Picuris Pueblo history

Indigenous peoples of the Americas have documented their history through rich oral traditions, encompassing narratives, storytelling, songs, poetry and music^35^, as well as through painted and sculpted art forms^27,31,36^. Whereas there is some debate whether those Indigenous histories can be co-interpreted alongside archaeological and/or genetic evidence and how^37–41^, there is a general agreement that Indigenous narratives possess both symbolic and literal meanings, recording historical events^42,43^ and probably others detected archaeologically^29,44^. However, it is vital to recognize that oral narratives have many functions, and they may concentrate on a cosmological dimension or a character’s intentions instead of more quotidian questions such as when and where^22,25^. Furthermore, oral narratives thrive in their plurality, as alternative, complementary accounts, and do not seek consensus to establish their importance^25,26,28^.

Picuris trace their origins to an underground realm inhabited by *Pá à wíá è Páyó*, the Creator, and other peoples. The community is divided in two halves (the ‘North’ and ‘South’ people), which have separate birthplaces in Picuris oral histories. These peoples were sent to the present, surface world by the Creator, some on clouds, others by watery routes, with the earliest peoples emerging from lakes near big white mountains. One of those lakes is called *Phaxwii Oxwalna*, and thought to be Serpent Lake, near Jicarita Peak, a mountain with perennial snow. It is located south of Picuris and, fittingly, the origin of the ‘South’ people. The geographical correspondence of the ‘North’ people’s emergence location is less clear, with some traditions identifying it as a spring near Pikes Peak, Colorado. After migrating through the landscape for an unknown duration of time, these peoples settled at Picuris and Taos Pueblos, as well as Pot Creek Pueblo, which is located between the two. Archaeological estimates put the founding of these communities at roughly 900 ce. Although Pot Creek Pueblo was depopulated in roughly 1320 ce, Picuris and Taos, communities that speak closely related languages, were not, and represent some of the oldest, continuously occupied communities in the Americas.

## Ancestral and present-day Picuris genomes

In dedicated ancient DNA clean laboratories at the Center for GeoGenetics, we extracted DNA^45,46^ and built 29 double-stranded libraries^47,48^ and 42 uracil-specific excision reagent (USER)-treated single-stranded libraries^49,50^ of 16 ancient individuals buried in Picuris Pueblo, dated between 500 and 700 years ago (median depth of coverage, 2.67×, ranging from 0.009× to 36.6×). All non-USER-treated libraries showed positive evidence of post mortem ancient DNA damage^51^, and we estimated present-day human contamination to be below 2% for all individuals^52–54^ (Supplementary Tables 1 and 2 and Supplementary Information 4 and 5).

At the community request, we also sequenced 13 present-day genomes from enrolled members of the Picuris Nation (Supplementary Table 3). We co-analysed these data with 590 ancient and modern genomes from the Americas and Siberia (Methods and Supplementary Information 9.1).

For all individuals, we obtained imputed diploid genotypes for the 44,136,290 variable sites in the 1000 Genomes high-coverage dataset, using GLIMPSE v.1.1.1 (refs. ^55–57^) (Supplementary Table 4 and Supplementary Information 9.1.2). As the genetic data of many Indigenous North American groups are available only as genome-wide arrays or hybridization methodologies, we also subset our dataset accordingly to allow a direct comparison between the newly generated data and the wealth of sequences in the literature^58,59^ (Methods, Supplementary Tables 5 and 6, and Supplementary Information 9.2).

We used principal component analysis (PCA)^60^, *f* statistics^61^, patterns of sharing of identity-by-descent (IBD) segments^62^ and estimated pairwise branch lengths^5^ to explore the genetic affinities between Ancestral and present-day Picuris, and all other Indigenous Americans with available genome-wide data, including the published low-coverage data from elite burials in Pueblo Bonito^10^ (Supplementary Information 10, 11.1, 11.2, 14.2 and 16).

**Fig. 2:**
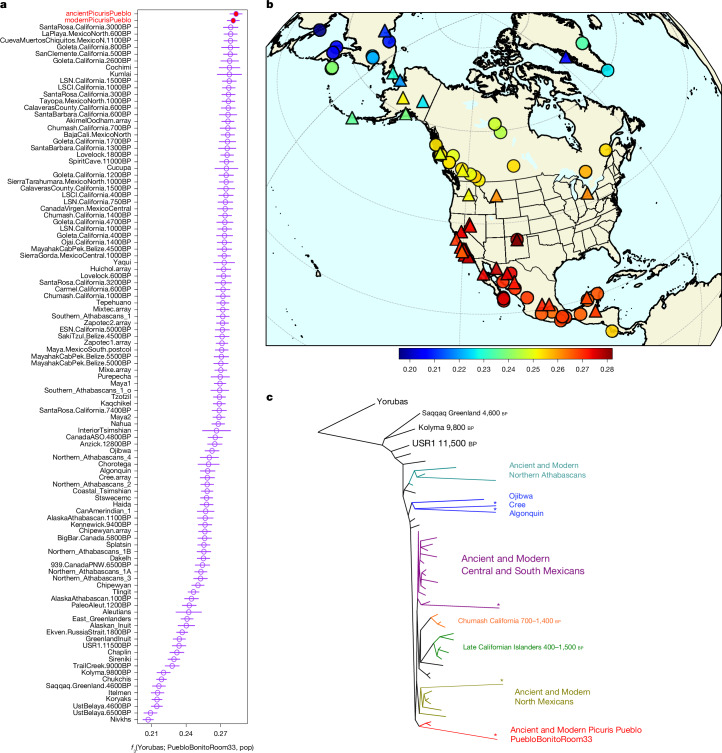
Allele-frequency-based analyses between ancient and present-day Picuris individuals with other Indigenous American populations. **a**, Outgroup *f*_3_ statistics in the format *f*_3_(PuebloBonitoRoom33, pop, Yorubas), in which we find present-day and ancient Picuris Pueblo to share the most shared genetic drift with ancient individuals from Pueblo Bonito. Error bars represent about 3.3 standard errors (*P* ≈ 0.001 in a *Z* test with 172,863 SNPs in 5-Mb jackknife blocks). **b**, Same outgroup *f*_3_ statistics results, plotted on a map of North America. Warmer colours indicate higher *f*_3_ values. Triangle and circle denote ancient and present-day individuals, respectively. **c**, Treemix maximum-likelihood tree of Indigenous North Americans, rooted using a West African population (Yorubas). Populations were grouped and branches were shortened (denoted with a *) for increased readability. Unedited results can be found in Extended Data Fig. 3.

**Extended Data Fig. 1:**
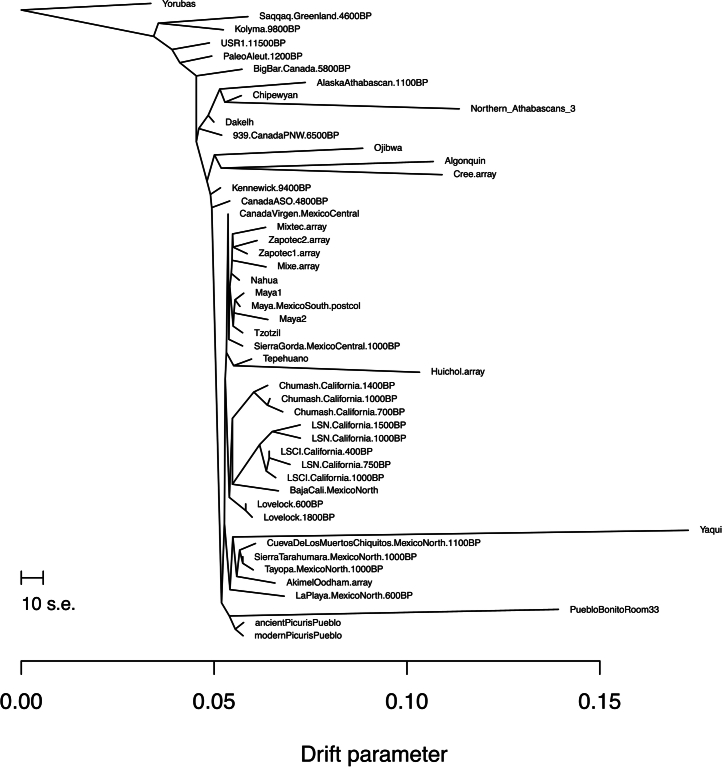
Maximum-likelihood tree built using Treemix on a dataset of North American Indigenous populations. The high branch lengths in some populations may reflect analytical artifacts: for ancient individuals, they arise from high error rates due to low coverage and DNA damage, while for present-day individuals, they result from missing data caused by masking non-Indigenous American segments.

**Extended Data Fig. 2:**
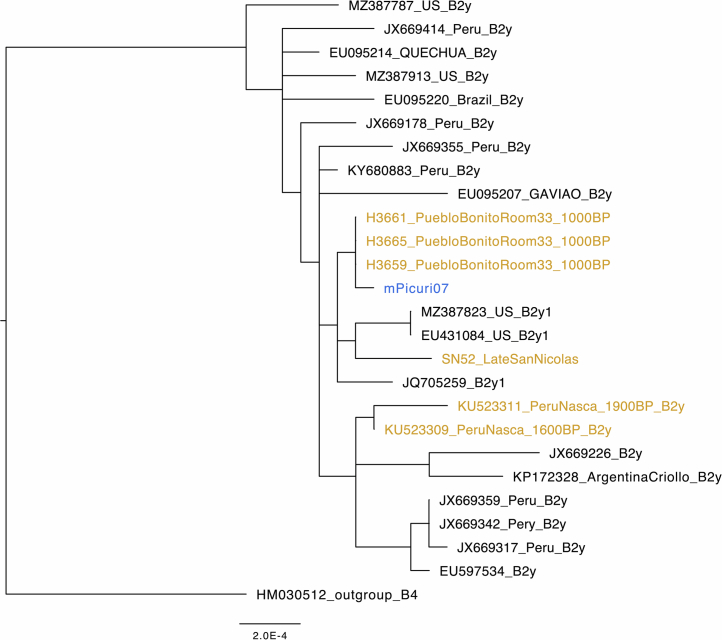
Maximum-likelihood tree of mitochondrial haplogroup B2y. Present-day literature mitogenomes are in black, present-day Picuris in blue and all other ancient mitogenomes in gold. Near-zero length branches were collapsed for convenience.

**Extended Data Fig. 3:**
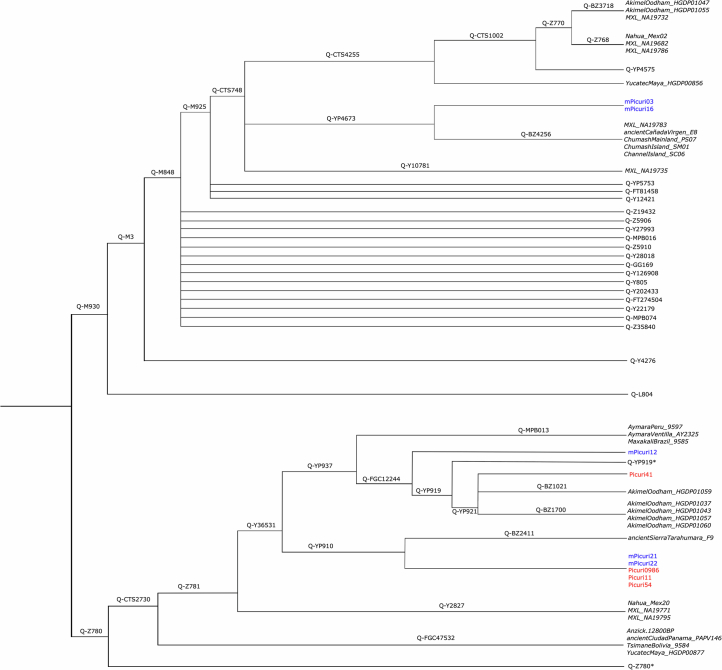
Placement of ancient and present-day Picuris in a schematic Y-chromosome phylogeny of haplogroup Q-MPB001/M1107. Ancient Picuris are in red and present-day Picuris in blue. Relevant literature individuals appear in italic. In accordance with Picuris wish to not have its data included in any private database, we do not describe any variant private to them.

We find that Ancestral and present-day Picuris are most closely related to each other, confirming the genetic continuity in Picuris Pueblo throughout the last millennium. Outgroup *f*_3_ statistics, *D* statistics, TreeMix^63^ and PCA results unambiguously show that no other sampled population, ancient or present-day, is more closely related to Ancestral Puebloans from Pueblo Bonito than the Picuris individuals are (Fig. 2, Supplementary Figs. 10–12 and 15–25 and Extended Data Fig. 1). These results are supported by Y-chromosome^64^ and mitochondrial DNA^65^ haplogroup distributions (Extended Data Figs. 2 and 3 and Supplementary Information 7 and 8). We acknowledge, however, that DNA sampling in the region is very limited and includes no other Pueblo communities. Nonetheless, sampled individuals buried in Room 33 in Pueblo Bonito all carried the same very rare mitochondrial haplotype (B2y1), suggesting a matrilineal dynastic system. We found one modern Picuris individual to carry this same haplotype, and to be closer to the Pueblo Bonito individuals than to any other sampled population in both maximum-likelihood and Bayesian analyses (Extended Data Fig. 1 and Supplementary Fig. 9).

## The broader genomic history of Picuris Pueblo

Previous studies have shown that Native American genomic ancestry featured two early splits, taking place between 22 and 14.6 thousand years ago (ka)^7,58,59,66,67^, giving rise to three main lineages. The first group to diverge were Ancient Beringians, represented by 11.5–9-ka-old individuals from Alaska^7,67^. This is followed by a split that gave rise to two extra lineages; one that includes mainly speakers of Athabascan and Algic languages, as well as groups in the Pacific Northwest^58,59^, whereas the other comprises all other previously studied Native Americans, including all ancient South Americans, and are represented by a roughly 12.8 ka individual of the Clovis culture (‘Anzick1’)^5^. However, many Indigenous groups have a complex population history that involves ancestry from above this split, from both lineages or ancestry related to the lineage represented by Anzick1, but not present in Anzick1 itself^5,7,9,68–71^ (Supplementary Information 11.2.2).

Using *D* statistics of the form *D*(ancientPicurisPueblo, Karitiana, Anzick, Yorubas)^5,61^ we found a significant rejection (*D* = −0.017; *Z* = −4.19) of ancient individuals from Picuris forming a clade with Southern Amazonian Karitiana to the exclusion of Anzick1, indicating Picuris carries ancestry that is not entirely from the lineage represented by Anzick1, a similar signal found in some North Mexican populations^7,71,72^ (Supplementary Figs. 25–31). However, when directly testing whether those populations carry instead ancestry from the other lineage in the form *D*(pop, Karitiana, test, USR1.11,500 bp) we found all to be consistent with zero (maximum *D* = 0.011, *Z* = 1.82 for LaPlaya.600 bp and ‘CanAmerindian_1’), suggesting our dataset does not include a suitable proxy for the excess ancestry not represented in Anzick1 found in Picuris and other populations. This probably represents either ancestry more basal than the one found in the Anzick1 genome, or an ancestry source very distantly related to the ones with genetic data available.

**Fig. 3:**
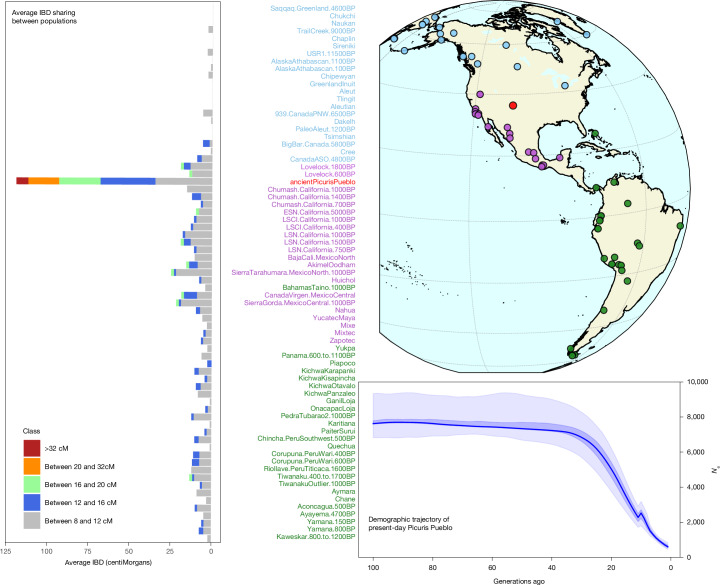
Haplotype and IBD analyses between ancient and present-day Picuris individuals with other Indigenous American populations. Left, the average shared IBD segments longer than 8 centiMorgans (cM) between Indigenous American populations and present-day individuals from Picuris Pueblo. Populations are ordered by latitude and coloured by major geographic areas. Right, the demographical history of present-day Picuris individuals inferred using HapNe-LD with imputed diploid genotypes. Shaded areas show 95 and 90% confidence intervals, and the dotted line the median estimate. The *x* axis is the time (measured in generations ago since the time of sampling), and the *y* axis the effective population size (*N*_e_).

To investigate whether the end of construction and occupation of Chacoan Great Houses in the North American Southwest roughly 1150–1200 ce was followed or preceded by a population bottleneck, we applied three different methods^73–75^ on the imputed diploid genotypes from ancient and modern Picuris separately to estimate founder effects and their effective population size (*N*_e_) over time. We estimate a similar population size in Picuris before European arrival using both ancient and present-day individuals, while also detecting no signal of population decline in pre-Colonial time using the three methods (Fig. 3, Supplementary Figs. 37 and 38 and Supplementary Information 12 and 15).

Archaeological and ethnohistoric estimates put the population of Picuris Pueblo on the eve of European arrival at around 3,000 individuals^76–78^. Architectural room-count-based estimates for the Northern Tiwa area in general (which includes Picuris), suggest that around 10,000 individuals occupied the region roughly 1300–1350 ce (ref. ^77^). Although it is challenging to translate effective population size estimates to census sizes in human populations, effective population sizes are conservatively estimated to roughly one-third of the census size^79^ or of a ratio between 0.21 and 0.65 (refs. ^73,80^). Therefore our point estimates, calculated both with ancient (harmonic mean over the ten last generations: 3,190 using IBD*N*_e_ (ref. ^73^) and 5,300 using HapNe-LD^75^) or present-day Picuris Pueblo members (harmonic mean between 20 and 30 generations, 5,480 using HapNe-LD) are consistent with estimates of around 10,000 individuals being part of the Picuris sphere of influence at the time. The estimate of around 3,000 people at Picuris Pueblo is supported by oral traditions at Picuris, as well as archaeological estimates based on ceramic frequencies and architectural space occupied at the site’s maximal size.

## Athabascan migration into the US Southwest

Using these high-quality genomic data, we sought to investigate the relationship of a Pueblo population with other populations, such as Athabascan-speaking groups. The latter occupy a large contiguous region in interior Alaska and western Canada, with two outlier populations residing along the Pacific Coast of South Oregon and Northern California, and the other in the US Southwest (the so-called Southern Athabascans)^81^. Whereas a north-to-south direction for their migration is attested in oral tradition^82^ and universally accepted among scholars^83^, its timing, causes, source population(s) and routes, and arrival times in those regions have been of longstanding academic debate.

The oldest direct archaeological evidence of Southern Athabascan settlement in the Southwest is a proposed Navajo-affiliated site in the Gobernador area of northwestern New Mexico, with a tree ring date of 1541 ce (ref. ^84^), a date that is coincident with the oldest written colonial record of a Southern Athabascan population—the ‘Querechos’, commonly identified as Apaches^85^—in the region. The archaeological Tierra Blanca complex has been tentatively suggested by some to be culturally associated with these groups, and would push the date of an Athabascan presence back to roughly 1450 ce (refs. ^83,85^). One hypothesis suggests this movement took place due to the cold climate of the Little Ice Age (which peaked in North America roughly 1400 ce)^86,87^, the colder conditions leading some northern groups to move south. In contrast to this hypothesis, other researchers have proposed that there was a substantially earlier Southern Athabascan presence in the region, perhaps by roughly 800 ce. The lack of archaeological evidence in support of this hypothesis is attributed to the group’s low census size and high mobility, which would have left relatively few archaeological traces^14–16^.

**Fig. 4:**
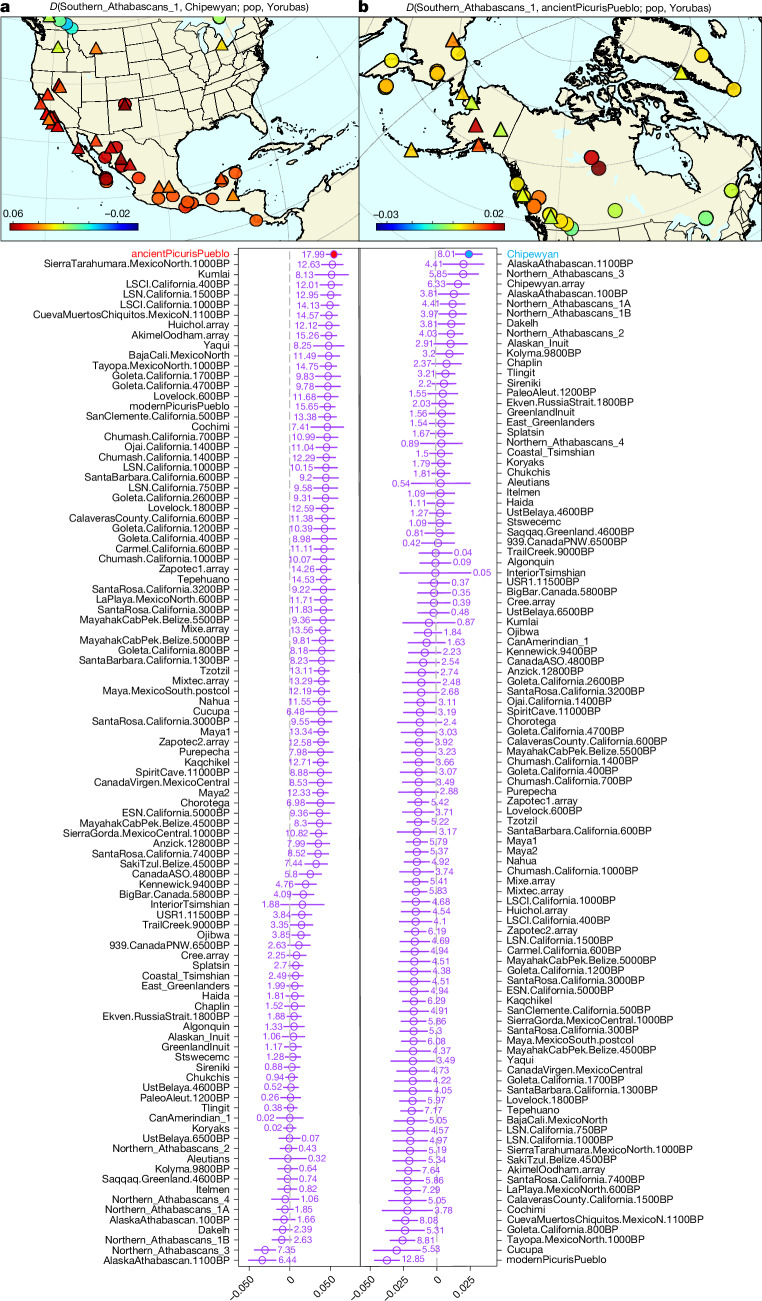
Relationship between ancient Picuris individuals and present-day Southern Athabascans. **a**, *D* statistics in the format *D*(Southern_Athabascans_1, Chipewyan, pop, Yorubas), where warmer colours indicate higher values of *D*, therefore a stronger genetic similarity with Southern Athabascans. Circles and triangles denote present-day and ancient individuals, respectively. Ancestral Puebloans from Picuris Pueblo is the population that maximize the value of *D*. Full *D* statistics results can be found in Supplementary Fig. 36. **b**, *D* statistics in the format *D*(Southern_Athabascans_1, ancientPicurisPueblo, pop, Yorubas). Athabascan speakers from northern North America are the ones to maximize the value of *D* (for example, Chipewyan). Full *D* statistics results can be found in Supplementary Fig. 37. For both plots, error bars represent about 3.3 standard errors (*P* ≈ 0.001 in a *Z* test with 172,863 SNPs in 5-Mb jackknife blocks).

To investigate whether it is possible to resolve the timing of Athabascan dispersal, we compared the Southern Athabascan individuals reported in ref. ^59^ with all individuals in our dataset. Using *f* statistics^61,88^, we found that Southern Athabascans can be modelled as receiving most of their ancestry from a Puebloan source (ancientPicurisPueblo) alongside a Northern Athabascan population (for example, Chipewyan) (*P* = 0.34, Fig. 4, Supplementary Figs. 33 and 34 and Supplementary Information 11.2.3). This is in line with the oral history of Southern Athabascans from the time of their arrival in the Southwest^82^, ethnographic evidence of many clan names being of ultimately Puebloan origin^89–91^, loanwords from Puebloan languages^92^ and, critically, the adoption of a more sedentary lifestyle and cultivation of maize, squash, beans, cotton and tobacco.

Southern Athabascan traditions link them to interactions with Ancestral Puebloan communities before sixteenth century European Contact, but we do not find evidence of Athabascan ancestry in any Ancestral Picuris individual predating 1535 ce; such ancestry only occurs among present-day Picuris individuals (Supplementary Figs. 22 and 23 and Supplementary Information 11.2.1 and 13). These results support a late arrival of Athabascan-related populations in the US Southwest, most likely after 1500 ce. Puebloan marriage preferences, burial practices, or admixture evident in Southern Athabascans could have been one-directional, as for example through Pueblo captives. Although this would explain the absence of an Athabascan ancestry signal in ancient Picuris, given the extensive interactions between those two groups, both historically as well as to this day, it is unlikely that such a scenario persisted over an extended period.

## Conclusion

In conclusion, we show that individuals from Picuris Pueblo are the closest sampled population, past or present, to ancient individuals from Chaco Canyon, challenging claims of depopulation or disappearance in the area, and establishing a genetic component to suspected cultural affiliation between a present-day group and Ancestral Puebloan heritage. We emphasize that this conclusion does not challenge or call into question the connections and relationships that more than two dozen federally recognized Tribes have to Chaco Canyon. It is, however, the only example of paleogenetic data supporting a federally recognized Tribe’s affiliation with Chaco Canyon ancestors.

Whereas Indigenous oral histories are increasingly being accepted and legislated as evidence of cultural affiliation, as they should be, caution is also needed to avoid unintentionally excluding groups such as Picuris Pueblo who have lineal and cultural affiliation, but who may have notable gaps in their histories due to centuries of continuing systemic injustices and colonial rule. Scientific tools, such as genetics, archaeology and linguistics, can assist in demonstrating local genetic continuity^7,8,93–96^ and provide opportunities to mend gaps in oral histories. Their application can have profound restorative value for present-day communities, discussions of their cultural heritage and ensure their federally recognized rights as stakeholders of ancient sites.

Finally, a crucial aspect of this research has been the shared commitment to ensure data sovereignty for Picuris Pueblo throughout this collaboration. Tribal leadership was involved in both drafting the research agreement and crafting the key findings of the work. Furthermore, Picuris Pueblo retained the right to curtail research at any point in the collaboration, and publication of results continues to be the sole decision of Picuris leadership. These guidelines and policies were an essential aspect of this collaborative research between non-Indigenous researchers and an Indigenous stakeholder community^3,97,98^. We hope collaborative efforts such as this one serve as catalysts for meaningful action and policy consideration, greater respect for oral histories and traditions, and that these results can be incorporated into well informed decision-making processes affecting Tribal sovereignty and community identity.

## Methods

### Community engagement

Following the decision of Picuris Pueblo members to study their population history using DNA, their archaeological collaborators initiated contact with the Centre for GeoGenetics in the University of Copenhagen. After 2 years of consultation and the signing of a research agreement, ancient individuals from Picuris Pueblo on long-term loan to Southern Methodist University were sent to the Centre for GeoGenetics. Saliva samples from present-day members of Picuris Pueblo were collected by members of the community following the signature of an informed consent that was explained by a Tribal liaison in both English and Northern Tiwa. Ethical oversight was provided by the Picuris Pueblo Tribal Council, which reviewed the study and granted permission for its conduct, with individual and tribal consent obtained from all participants. Furthermore, we consulted the National Committee on Health Research Ethics in Denmark, which had no comments on the study design H-16018872 (Supplementary Information 1–3).

### Laboratory procedures

All ancient DNA work was performed in dedicated clean laboratories at the Centre for GeoGenetics, GLOBE institute, University of Copenhagen. We extracted DNA from between 50 and 100 mg of crushed bone powder obtained from tooth cementum and the pars petrosa of the temporal bone of 28 samples from 22 ancient individuals. We built 34 double-stranded and 54 single-stranded Illumina libraries from those extracts, the latter after USER treatment. DNA was extracted from present-day saliva samples using Qiagen DNeasy Blood & Tissue Kit and built into dual-indexed Illumina libraries. All libraries were paired-end sequenced (2 × 100 bp) on an Illumina NovaSeq 6000 platform at the GeoGenetics Sequencing Core (Supplementary Information 4).

### Radiocarbon dating

Accelerator mass spectrometry was performed on the University of California Irvine Keck-CCAMS facility to obtain direct radiocarbon dates. Calibrated dates were obtained with Bayesian modelling done using OxCal v.4.4 (ref. ^100^) software using the Northern Hemisphere curve^101^.

### Sequencing data processing and ancient DNA authentication

Raw sequencing data were demultiplexed and had their adaptors trimmed using AdapterRemoval v.2.3.2 (ref. ^102^). Collapsed and paired-end reads were mapped to GRCh38 using bwa aln v.0.7.17 (ref. ^103^), optical duplicates were marked using Picard v.2.25.0 and depth of coverage and average read length estimated using pysam v.0.22.1. Ancient DNA characteristic misincorporation patterns were measured using mapDamage v.2.0 (ref. ^104^), and present-day contamination using ContamMix^52^ and ANGSD v.0.931 (ref. ^53^). For all analyses, only reads with mapping quality greater than or equal to 30 and bases with quality equal to or greater than 20 were considered. Chromosomal sex was estimated by estimating the ratio of reads mapping to the autosomal, X- and Y-chromosomes (Supplementary Information 5 and 6).

### Mitochondrial DNA analysis

Consensus sequences were obtained using samtools v.1.3.1 mpileup^105^), requiring a minimum of five reads and more than 70% frequency in a locus to call a base. Haplogroup assignment was done using Haplogrep v.3 (ref. ^106^). Following sequence alignment using mafft^107^, haplogroup-specific phylogenetic trees were built using raxml-ng v.0.8.1 (ref. ^108^) and BEAST v.2.6 (ref. ^109^) (Supplementary Information 7).

### Y-chromosome analysis

Genotypes for positions located in the single-copy short-read callable 10-Mb region of the Y-chromosome^110^ were called using bcftools v.1.17 mpileup, excluding triallelic locus, indels and variants called in less than 95% frequency in the locus. Haplogroups were called by matching ancestral and derived calls to ISOGG 2019–2020, private databases and a previously published curated database^111^ (Supplementary Information 8).

### Reference datasets

We compared Picuris Pueblo present and ancient individuals with 835 published present-day and ancient genomes from the literature^5–7,59,67,68,72,93–95,112–123^, alongside the 2,503 individuals from the 1000 Genomes high-coverage dataset^124^. Furthermore, as data from some key population only exists as hybridization capture or single-nucleotide polymorphism (SNP) array^10,58,69,71,125–133^, we subset our whole genome dataset to those to allow a direct comparison. In total, we compared Picuris Pueblo data with more than 300 populations from the American continent and more than 5,500 individuals worldwide (Supplementary Information 9).

### Imputation and phasing

We used GLIMPSE v.1.1.1 (ref. ^55^) to impute ancient and present-day genomes with depth of coverage above 0.3× on a set of 44,136,290 variable sites on the 1000 Genomes high-coverage dataset, which was used as a reference panel. We excluded individuals with average genotype probability below 0.98. For the remaining individuals, we excluded variants with an INFO score below 0.8, not present in the GRCh38 strict accessibility mask and with a reference panel allele frequency below 1% (Supplementary Information 9).

### Population structure analyses

We performed PCA on the dataset as implemented in plink v.1.90 (ref. ^134^), first including all individuals, then restricting to Far East Asia and the Americas and finally only including North American individuals. We used ADMIXTURE^63^ to investigate ancestry proportions and detect the presence of non-Indigenous American ancestry. We masked African and European tracts in present-day Indigenous Americans using RFMix v.2.03-r0 (ref. ^135^) (Supplementary Information 9 and 10).

### *f* statistics

Using FrAnTK^136^, we computed *f*_3_ statistics to estimate shared drift between two populations and *D* statistics to formally test hypothesis of cladality and gene flow^61^. We used qpAdm^88^, as implemented in Admixtools 2 (ref. ^137^), to model individual and population ancestry proportions (Supplementary Information 11).

### IBD segment sharing

We identified segments in IBD in the whole genome dataset using IBDseq^138^, but only segments with an LOD score higher than three and that were longer than two centiMorgans, and removing regions with excess IBD sharing following^138^. IBD sharing between individuals was averaged out per population (Supplementary Information 14).

### Estimation of demographic trajectories, admixture time and recent bottlenecks

To test the hypothesis of a pre-Colonial depopulation event taking place in the North American Southwest, we estimated demographic trajectories using IBD segments with IBD*N*_e_ (ref. ^73^) and using patterns of linkage disequilibrium using HapNe-LD^75^. Age of founder population and bottlenecks were estimated using ASCEND^74^, and timing for admixture events with DATES^139^ (Supplementary Information 12, 13 and 15).

### Reporting summary

Further information on research design is available in the Nature Portfolio Reporting Summary linked to this article.

## Online content

Any methods, additional references, Nature Portfolio reporting summaries, source data, extended data, supplementary information, acknowledgements, peer review information; details of author contributions and competing interests; and statements of data and code availability are available at 10.1038/s41586-025-08791-9.

## Supplementary information

Supplementary Information

Reporting Summary

Supplementary Tables Supplementary Table 1: Per library sequencing summary statistics. Supplementary Table 2: Per individual sequencing summary statistics. Supplementary Table 3: Present-day Picuris individuals sequencing summary statistics. Supplementary Table 4: Reference genomic dataset summary statistics. Supplementary Table 5: Reference hybridization capture dataset. Supplementary Table 6: Reference array dataset.

## Data Availability

All maps were made using freely available vector and raster data from Natural Earth or the US Geological Survey and plotted using ESRI ArcGIS Pro v.3.0 and cartopy v.0.24.1. Compressed sequence alignment map files (BAM) aligned using human reference genome GRCh38 for ancient and present-day individuals are available in the European Genome-Phenome Archive under accession numbers EGAD50000001245 and EGAD50000001246. Picuris Pueblo is the sole owner of this dataset. Data access is controlled by the Picuris Pueblo Tribal Council through standard signed agreements that regulate the use of the ancient and/or present-day data. Consent for access will be granted exclusively to universities and research institutions exclusively for studies in population genetics that focus on understanding population history. The agreement strictly prohibits the use of the data for commercial purposes, inclusion in any kind of private databases (including forensic, mitochondrial or Y-chromosome) or medical or natural selection studies. Data cannot be used for tribal enrolment purposes. Present-day Picuris genomes are available exclusively for replication purposes using exactly identical comparison datasets, parameters and software and are controlled under a different agreement. Requests for data access will be evaluated jointly by the authors and the currently serving Picuris Pueblo Tribal Council every 3 months during the first year since publication, and every 6 months thereafter. Legitimate medical applications will be considered by the Picuris Pueblo Tribal Council if they explicitly serve Picuris health interests and guarantee that Picuris Pueblo retains full ownership of the data at all times and the right to halt any project at its sole discretion. Furthermore, previously published genomic data used for comparison are detailed and referenced in Supplementary Tables 4–6. They were obtained from the following sources: access granted by authors^58,120,122,131–133^; custom repositories (https://ftp.1000genomes.ebi.ac.uk/vol1/ftp/data_collections/)^120,121,124^; European Nucleotide Archive: SRX381032 (ref. ^5^), SRS93795 (ref. ^6^), PRJEB20398 ref. ^7^), SRP094965 (ref. ^10^), PRJEB9733 (ref. ^59^), PRJEB20398 (ref. ^67^), PRJEB25445 (ref. ^68^), PRJEB28961 (ref. ^69^), PRJEB66319 (ref. ^71^), PRJEB51440 (ref. ^72^, PRJEB24629 (ref. ^93^), PRJEB22578 (ref. ^94^), PRJEB42372 (ref. ^95^), PRJEB41550 (ref. ^112^), PRJNA883375 (ref. ^113^), SRA010102 (ref. ^114^), PRJEB37726 (ref. ^115^), PRJNA470966 (ref. ^116^), SRP029640 (ref. ^117^), PRJEB29700 and PRJEB26336 (ref. ^118^), PRJEB30575 (ref. ^119^), PRJNA883976 (ref. ^123^), PRJEB39010 (ref. ^125^), PRJEB38555 (ref. ^126^), PRJEB37518 (ref. ^127^), PRJEB50901 (ref. ^128^), PRJEB37446 (ref. ^129^) and PRJEB49391 (ref. ^130^).

## Code availability

Analyses were done using freely available software, which have been fully referenced throughout the paper and Supplementary Information. Custom code can be found on https://github.com/abigailramsoe/lundbeck-pipeline.
