# Supplementary material for: Picuris Pueblo oral history and genomics reveal continuity in US Southwest
Source: Nature. 2025 Apr 30;642(8066):125–32. doi: 10.1038/s41586-025-08791-9 (PMC12137115; doi:10.1038/s41586-025-08791-9)
Supplement: Supplementary file 1 — Supplementary Information [file 41586_2025_8791_MOESM1_ESM.pdf]

nature portfolio

https://doi.org/10.1038/s41586-025-08791-9

Supplementary information

Picuris Pueblo oral history and genomics reveal continuity in US Southwest

In the format provided by the
authors and unedited

Picuris Pueblo oral history and genomics reveal continuity in US Southwest

Supplemental Information

---

1. Statement on Ethics and Language

2. Community engagement with Picuris Pueblo

3. Contextualizing the history of Picuris Pueblo
- **3.1. Picuris Traditional knowledge in relation to Chaco Canyon**
- **3.2. Archaeological context of Picuris Pueblo**
- **3.3. Historical context of genetic studies in the North American Southwest**
- **3.4. Linguistic context of Picuris Pueblo**
- **3.5. Current archaeological models for the peopling of the Northern Rio Grande Region**

4. Sampling and laboratory procedures
- **4.1. Ancient individuals**
- **4.2. Present-day saliva samples**

5. Mapping, post-processing and ancient DNA authentication

6. Chromosomal sex inference

7. Mitochondrial DNA analyses

8. Y-chromosome analyses

9. Dataset curation
- **9.1. Whole genome dataset**
  - **9.1.1. Remapping**
  - **9.1.2. Imputation and phasing**
  - **9.1.3. Local ancestry and masking of non-Indigenous American segments**
- **9.2. Array-based dataset**

10. Principal Component Analysis (PCA) and genetic clustering

11. *f*-statistics
- **11.1. Outgroup *f*3-statistics**
- **11.2. *D*-statistics and qpAdm**
  - **11.2.1. Population continuity in the North American Southwest**
  - **11.2.2. Relationship with SNA and NNA groups**
  - **11.2.3. Modelling the ancestry of Southern Athabascans**

12. Founder population age and bottleneck estimation

13. Estimating the timing of admixture at present-day Picuris Pueblo

14. Identity-by-Descent (IBD) segment sharing
- **14.1. Detection and validation of Identical-by-Descent (IBD) segments**
- **14.2. Average IBD sharing profile with other populations**

15. Estimation of population demography in recent time

16. Pairwise branch lengths

1. Statement on Ethics and Language

Some of the publicly available comparative reference genetic data used in this study are from biological samples collected decades ago, and it is impossible to ensure consent was informed and voluntary, and whether the participant population(s) understood the risks, benefits, and, crucially, future potential uses of these data. Our use of these data should not be seen as an endorsement of the past practices. More practically, these past practices make it difficult, if not impossible, to meaningfully consult those groups in the context of new analyses that use previously published data. As a result, this study does not involve community engagement with the several populations from which these earlier samples were obtained. In the case of data generated from museum "culturally unaffiliated" individuals, we concur with many others that lack of community engagement in these cases is an act of disrespect and a direct attack to communities' sovereignty and identity[1,2]. Our usage of these reference data has been directly requested by Picuris Pueblo, an Indigenous community with established cultural affiliation to Ancestral Pueblo groups in the U.S. Southwest.

In this work, we attempt, to our best knowledge, to use the term Indigenous groups use to refer to themselves in English. This include modifying literature metadata whenever necessary. For completeness, we describe the changes here below.

1) *Athabascan* instead of *Athapascan/Athapaskan/Athabaskan*. This is the preferred spelling by Alaska's Tanana Chiefs Conference[3].

2) *Ancestral Puebloan* instead of *Anasazi*. Both words are exonyms – "*Anaasází*" (Navajo = "ancestor of our enemies") and *Pueblo* (Castilian = "village"). Present-day Pueblo communities have voiced their discomfort with the Navajo term, as it has a negative connotation regarding Ancestral Pueblo identities. While some archaeologists and anthropologists have argued that the *Anasazi* in literature is detached of its original meaning, we see no reason for using a word that many descendant communities have explicitly argued against its usage. Many Pueblo communities have proposed terms in their own language to refer to their own ancestors, but given the lack of agreement on a single Indigenous name for all Pueblo ancestors, we default to Ancestral Pueblo.

*3) Akimel O'odham instead of Pima***.** "Pima Indians" is a term mainly used by early ethnographers, and members of the group do not refer to themselves as such.

4) Inuit instead of Eskimo. This is the preferred term by the Inuit Circumpolar Council, and it means '*people*' in most Inuit languages. Despite universal agreement against the term Eskimo, we note that some communities, such as the Yupik, do not use the term Inuit as it does not exist in their languages (Yupik = '*yuit*').

Additionally, we stress that genetic ancestry proportions may or may not correspond with identity; identity is a complex aspect of individual and community life which extends much beyond genetic makeup. Throughout the text, our discussion remains exclusively centered on genetic variation which is Indigenous American in origin, and we make no claims regarding the identity of present or past peoples. Furthermore, while we make use in the text of discrete labels and categories for genetic ancestry, we recognize them as useful simplifications, not faithfully representations of the nature of human ancestry[4].

Finally, we note that, for increased readability and description, we address both the Picuris Pueblo Tribal Nation and its members in the third person, despite some of its members being coauthors of this study.

2. Community Engagement with Picuris Pueblo

Active research collaboration between Picuris Pueblo and professional archaeologists started in the early 1960's when Dr. Herbert Dick from Southern Methodist University (SMU) initiated a multidisciplinary project, funded by the U.S. National Science Foundation (NSF), to better understand Picuris' ancestry[5]. Over the next decade, Dick involved community members, archaeologists, palynologists, zooarchaeologists and many others in the excavation of surface architecture, midden deposits, subsurface ritual and habitation features. Many of the recovered items were put on display in the Picuris Museum which opened in 1969. Parts or all of 113 surface rooms, 13 kivas and 1,660 linear feet of trenching exposed large areas of ancestral occupation dating back to approximately 900 CE or earlier. Well over 1 million artifacts were recovered, catalogued and summarized in a series of reports to NSF and the National Park Service[5,6]. Nearly all of these materials are stored in trust at Southern Methodist University's campus (SMU-in-Taos) located south of Taos, New Mexico.

As was common during these 20th century excavations, human remains were exhumed and stored with the associated archaeological collections on the SMU-in-Taos campus. Following the passage of the Native American Graves Protection and Repatriation Act in 1990, all human remains then known to be part of the collections were returned to Picuris Pueblo for reburial. These included culturally affiliated remains from Pot Creek Pueblo, one of Picuris Pueblo's ancestral villages, located on the SMU-in-Taos campus.

In 2018, several sets of human remains that had been stored in a locked storage room since Dr. Dick's death in 1992 were found. In discussions with Picuris tribal leadership to decide disposition of the remains, Picuris Governor Craig Quanchello asked if the DNA from the Picuris ancestral remains, if analysed, would show Picuris' genetic relationships with other Pueblos, including Ancestral Pueblo populations from Chaco Canyon. Following conversations with Michael Adler (Southern Methodist University, SMU) during 2019 and in 2020, the Picuris Pueblo Tribal Council decided to request that Adler contact Eske Willerslev to explore the possibility of a collaboration (see detailed timeline below). Researcher team members and Picuris Pueblo leadership subsequently held several online meetings to outline the scope and objectives of the project.

During 2020 and early 2021, the collaboration involved frequent communication focusing on ethical considerations, community consent and practical planning. Topics discussed with Picuris Pueblo leadership included the destructive nature of ancient DNA methods, the availability and spatial distribution of reference genetic datasets for comparison, and limitations and capabilities of genetic methods. Following these discussions, the Centre for GeoGenetics and Picuris Pueblo signed a Memorandum of Understanding (MoU) in June 2021. The MoU established several principles guiding the collaboration:

1) a major project goal is to expand the scientific understanding of Picuris Pueblo's genetic relationships with present and past communities in both the US Southwest and surrounding regions, as well as to investigate past demographic changes at Picuris Pueblo. Picuris Pueblo is to be fully informed through written communications and/or online meetings each time there are significant project results.

2) Picuris Pueblo tribal leadership has full control of the generated sequence data, and can request the termination of the project at any time. Future publication of research results is also fully controlled by tribal leadership.

3) Picuris Pueblo is supportive of historical genetics research with its data, and understands that the project is only possible due to existence of previous reference datasets being made available for researchers. Picuris Pueblo, however, strictly forbids commercial usage of its data, its usage by for-profit companies, its inclusion in private commercial databases of any sort, or the use of genetics data for tribal enrollment purposes.

4) All human remains, bone powder, DNA extracts and genomic libraries will be returned to Picuris for reburial.

Additionally, due to the wishes of Picuris Pueblo members to have a direct comparison point with those ancient individuals, conversations started between Picuris Pueblo lawyers and the University of Copenhagen for drafting an informed consent form and broader agreement governing personal data. Under this agreement, modern DNA sampling could be undertaken through the collection of saliva samples from consenting tribal members.

During 2021 and 2022, many online meetings took place between the research team – Picuris Pueblo leadership, Centre for GeoGenetics and SMU – to discuss initial findings, particularly on the nature of the genetic relationships between ancient individuals from Picuris and previously published low-coverage hybridisation capture data from individuals interred in Pueblo Bonito, Chaco Canyon. Picuris Pueblo Governor and Tribal Council advocated for the inclusion of the capture data from Chaco Canyon. In March 2023, a final collaborative agreement governing the sequencing of present-day individuals was presented by Picuris Pueblo lawyers and signed. Informed consent forms were explained in both English and Northern Tiwa by shared first author and by the Tribal Liaison of the project Richard Mermejo. These were then signed, and saliva was collected from voluntary members of the community.

In May 2023, Picuris Pueblo leadership requested that we jointly prepare a draft manuscript for submission to a scientific journal. Multiple meetings and communications focused on data-sharing governance after publication. Given that Picuris Pueblo currently lacks the infrastructure to support and curate the data safely, four options were considered, each with its own risks and benefits. Those options were: (1) data could be deposited in a University of Copenhagen repository under Picuris control; (2) data could be held by Indigenous-led initiatives in the US; (3) data could be deposited in a European Union access-controlled genetic archive (EGA) or (4) Picuris Pueblo could make the data fully public. Additionally, two trips were planned: one of the Centre for GeoGenetics researchers to Picuris Pueblo, and one of the Picuris Pueblo leadership to Copenhagen, Denmark.

In April 2024, the Centre for GeoGenetics team travelled to New Mexico (USA). During this visit, they met Picuris Pueblo leadership and the tribe's legal representatives to discuss and revise the draft manuscript following Picuris Pueblo feedback. The Picuris Pueblo Tribal Council decided on option 3 for data-sharing governance, depositing the data in EGA, and the data access agreements governing the ancient and present-day data were presented to the Picuris legal counsel. Next, the research results were presented Picuris community during a tribal meeting where all members were invited, with 48 of the 198 tribal members in attendance. Community members asked questions and shared their impressions and comments with the researchers and tribal leadership. Many of the questions concerned the treatment of the ancient individuals in Copenhagen laboratory, the return of ancestral remains, and future directions the collaboration could take. Specific interest focused on the application of genetic information to better understand past and present health challenges among Picuris community members, as well as applications to traditional medicine. The community later hosted a lunch

and guided researchers through the Pueblo, including visitation of spiritually important locations on tribal lands.

After the presentation to the community, the manuscript was approved by the Picuris Pueblo Tribal Council for submission. Following the first round of reviews, the revised version of the manuscript was again submitted to the Tribal Council for approval, which was granted in August 2024.

Below, we include a detailed chronology of the collaboration between Picuris Pueblo, Centre for GeoGenetics and Southern Methodist University (SMU). We include all meetings in which the project was discussed.

Meetings of the entire research team (Picuris Pueblo Governor Craig Quanchello, Tribal Council, Tribal Liaison of the project Richard Mermejo, Eske Willerslev, J. Víctor Moreno-Mayar, Thomaz Pinotti, Michael Adler and David Meltzer) are highlighted in bold.

05 Feb 2020	Picuris Pueblo leadership asked Michael Adler (SMU) to contact on their behalf ancient DNA researchers to study the recently discovered ancient individuals from the SMU-in-Taos campus.
06 Feb 2020	Adler contacted his colleague, David Meltzer (SMU) who in turn reached out to Eske Willerslev (Centre for GeoGenetics) to collaborate with Picuris Pueblo on the project they wished to initiate.
**25 May 2020**	**Online meeting between Governor Craig Quanchello, Tribal Liaison for the project Richard Mermejo and all Picuris Pueblo Tribal Council members with Michael Adler and David Meltzer (SMU) and Eske Willerslev and J. Víctor Moreno-Mayar (Centre for GeoGenetics). An agreement is reached to move forward with the collaboration.**
**11 Jun 2020**	**Online meeting between Governor Craig Quanchello, Tribal Liaison for the project Richard Mermejo and all Picuris Pueblo Tribal Council with Michael Adler and David Meltzer (SMU) and Eske Willerslev and J. Víctor Moreno-Mayar (Centre for GeoGenetics). Picuris approved Adler shipping the ancient Picuris individuals to Copenhagen.**
16 Jul 2020	Online meeting between Eske Willerslev, Fabrice Demeter (Centre for GeoGenetics) and David Meltzer and Michael Adler (SMU) to discuss next steps in analyses.
22 Jul 2020	Discussions between Dena Plemmons (UC Riverside) and Michael Adler (SMU) regarding the ethics and documentation of ancient DNA collaboration.
31 Jul 2020	Initial draft of Memorandum of Understanding (MoU) between GeoGenetics and Picuris Pueblo.
07 Oct 2020	Thomaz Pinotti (Centre for GeoGenetics) start lab work with ancient individuals from Picuris Pueblo.
**12 Mar 2021**	**Online meeting between Governor Craig Quanchello, Tribal Liaison of the project Richard Mermejo, Eske Willerslev, J. Víctor Moreno-Mayar, Thomaz Pinotti, Michael Adler, David Meltzer (henceforth, *the research team*), Devlin Gandy and Tribal Council on screening results.**
29 May 2021	Online meeting between Eske Willerslev, J. Víctor Moreno-Mayar, Thomaz Pinotti, Michael Adler and David Meltzer on first round of deep sequencing results, clear evidence of links with ancient individuals from Pueblo Bonito, Chaco Canyon.
**31 May 2021**	**Online meeting between research team and Tribal Council to share initial results of deep sequencing results.**
24 Jun 2021	Memorandum of Understanding (MoU) approved by Picuris Pueblo legal representatives and signed by all parties.
**12 Oct 2021**	**Online meeting between research team on more results, including findings relating to Chaco Canyon and relationship with Southern Athabascans.**
02 Feb 2022	Samples from two ancient individuals sent for C14 dating at UC Irvine radiocarbon laboratory.
07 Mar 2022	Online meeting between Eske Willerslev, J. Víctor Moreno-Mayar, Thomaz Pinotti, Michael Adler and David Meltzer to discuss next steps.
17 Oct 2022	Pinotti submits last round of sequencing of ancient individuals from Picuris Pueblo.
10 Mar 2023	Final collaborative agreement written by University of Copenhagen's and Picuris Pueblo's legal team signed by all parties.
**20 Mar 2023**	**Online meeting between research team to discuss final results using data from ancient individuals. Update on signature of inform consent forms and the collection of saliva samples.**
**28 Mar 2023**	**Online meeting between research team for planning next steps.**
30 Mar 2023	Saliva samples for voluntary Picuris Pueblo members arrive in Copenhagen.
02 May 2023	Online meeting between Eske Willerslev, J. Víctor Moreno-Mayar, Thomaz Pinotti, Michael Adler and David Meltzer following Picuris Pueblo Tribal Council request for publication of results in a scientific journal.

22 May 2023	Julie Bitz-Thorsen (Centre for GeoGenetics) start lab work with saliva samples from present-day individuals from Picuris Pueblo.
31 May 2023	Initial drafts for main text and supplemental information for the manuscript.
13 Jun 2023	Bitz-Thorsen submits last round of sequencing of present-day individuals from Picuris Pueblo.
02 Aug 2023	Online meeting between Eske Willerslev, J. Víctor Moreno-Mayar, Thomaz Pinotti, Michael Adler and David Meltzer to discuss edits to manuscript.
**04 Oct 2023**	**Online team between research team and Tribal Council to discuss current version of the manuscript and plans for submission.**
14 Dec 2023	Online meeting between Eske Willerslev, J. Víctor Moreno-Mayar, Thomaz Pinotti, Michael Adler and David Meltzer to discuss edits to manuscript.
27 Jan 2024	Shared first author, former Picuris Pueblo Governor and Tribal Liaison of this project, Richard Mermejo, passes away at 78.
02 Feb 2024	Pinotti defends his PhD thesis at University of Copenhagen, which is dedicated to the memory of Richard Mermejo.
04 Mar 2024	Online meeting between Eske Willerslev, J. Víctor Moreno-Mayar, Thomaz Pinotti, Michael Adler and David Meltzer to discuss edits to manuscript and plan the trip to Picuris Pueblo.
**27 Mar 2024**	**Online meeting between Eske Willerslev, J. Víctor Moreno-Mayar, Thomaz Pinotti, Michael Adler, David Meltzer, Picuris Pueblo Tribal Council and Picuris Pueblo legal representatives meeting to discuss details of data-sharing governance.**
**12 Apr 2024**	**Eske Willerslev, J. Víctor Moreno-Mayar, Thomaz Pinotti, Michael Adler and David Meltzer travel to Santa Fe, New Mexico, to meet with Picuris Pueblo Tribal Council and their legal representatives. Following discussion and after incorporating suggestions, the 'Santa Fe version' of the final manuscript is approved by the Tribal Council for submission to a scientific journal.**
**13 Apr 2024**	**Eske Willerslev, J. Víctor Moreno-Mayar, Thomaz Pinotti, Michael Adler and David Meltzer travel to Picuris Pueblo to present research results to community members during a tribal meeting, following discussion and a lunch hosted by Picuris Pueblo.**
14 Apr 2024	Picuris Pueblo community members are invited to visit SMU-in-Taos campus and Pot Creek Pueblo.
06 May 2024	Manuscript submitted to *Nature*.

3. Contextualizing the history of Picuris Pueblo

3.1. Picuris Traditional knowledge in relation to Chaco Canyon

In regard to Picuris traditional knowledge regarding Chaco Canyon, co-author Richard Mermejo points to strong linkages between the traditional racetracks and raceways used by Picuris Pueblo to the system of "Chaco roads" that have been documented across the Chacoan interaction region[7]. Like the Chaco roads, the system of racetracks made and used by the Picuris community serve to physically and spiritually connect Picuris Pueblo with sacred locations and historically important relationships with other pueblo communities. One example is the raceway that connects Picuris with Jicarita Peak, one of the most important locations in the Picuris ritual landscape. This raceway was used up until the 1950s for ritual races of the two community moieties up to Jicarita Peak during certain annual ceremonials. Use of the Jicarita raceway ended when local landowners fenced private lands, preventing use of the traditional raceway. A second important raceway described by Richard Mermejo connects Picuris with the Ojo Caliente locale in the Chama River Drainage. This raceway continues past Ojo Caliente, to the lithic sources at Pedernal Peak, and according to Richard, stretches into the San Juan Basin, the center of the Chaco regional interaction realm. Finally, Richard Mermejo identifies certain of the painted motifs in kivas excavated by Herbert Dick in the 1960s[8], as symbols linking Picuris to Chaco Canyon. Mermejo adds that the details of these symbolic linkages are not to be discussed outside of traditional Picuris contexts.

3.2. Archaeological context of Picuris Pueblo

Picuris Pueblo, home to Picuris village life for over a millennium, is located in the northeasternmost region of Ancestral and present-day Pueblo occupations in the Greater Southwest. The trajectory from mobile, foraging strategies to more sedentary, agrarian village life occurred comparatively late in the northern Rio Grande relative to areas to the south and west. Foraging groups have been in this region since the late Pleistocene, c. 13,000 years ago. The earliest agrarian settlements in the region date to the late 9th or early 10th centuries CE, comprising scattered communities dependent on rainfall-fed agriculture, with these early farmers living in subterranean pit houses that likely housed extended family groups. The Picuris Pueblo occupation starts at around 900 CE and is generally divided in ten temporal phases, chronologically discernible by ceramic types and previous research conducted in the area [6]. For this study, we directly dated nine individuals (**Supplemental Table S07**), whose ages range from 1615 ± 20 radiocarbon years BP to 380 ± 20 radiocarbon years BP (corresponding to median calibrated ages of 473 CE to 1492 CE)[9]. The majority of the individuals date to the pre-contact period of occupation at Picuris Pueblo. The earliest individual (Picuri0986) predates the establishment of the Pueblo and agrarian settlement in the region, and likely was part of an ancestral forager-horticultural population in the Northern Rio Grande.

The evidence from the post-900 CE occupation at Picuris contrasts with contemporary occupations during this same time in areas such as Chaco Canyon, where agrarian communities were beginning to build multi-story masonry and adobe structures housing much larger residential groups. In fact, the largest architectural feature of Chaco Canyon, the Pueblo Bonito great house, comprised over 800 surface rooms and multiple pit structures (kivas) by the middle of the 11th century CE[10]. Pueblo Bonito is one of many great houses found across the Chaco regional integrative system, a complex of beliefs, architecture and community organization found across an estimated 115,000 square miles[11] in what is now western New Mexico, southern Colorado and eastern Arizona. This complex of features that defines the Chaco regional system is not found along the Rio Grande drainage, setting this area apart from the rest of the Ancestral Pueblo world during this time. Nonetheless, and as detailed in our study,

the lack of material cultural ties between the Northern Rio Grande and the Chaco regional system contrasts with the clear genetic ties between individuals buried in Chaco Canyon and ancestors of today's community of Picuris Pueblo.

individual	AutoDepth	karyosex	14C_age	stderr	*C14code* (UCIAMS)	median_calCE	from95%_calCE	to95%_calCE	median_calBP	from95%_calBP	to95%_calBP	d15N(‰)	d13C(‰)
**Picuri0986**	2.882	XY	1615	20	304716	473	415	538	1478	1536	1413	10.3	-10.7
**Picuri11**	2.587	XY	880	15	304717	1187	1157	1219	763	794	731	11	-7
**Picuri12**	2.863	XX	935	15	304721	1100	1040	1159	851	911	791	10	-7.1
**Picuri21**	4.168	XX	380	20	259753	1492	1451	1624	459	499	327	13,0	-5.9
**Picuri31**	2.770	XX	535	15	304718	1413	1396	1430	538	554	521	10.6	-6.3
**Picuri41**	21.593	XY	420	25	304722	1459	1432	1615	492	519	335	10	-7
**Picuri54**	5.619	XY	870	20	304719	1190	1054	1224	760	897	726	11.4	-6.7
**Picuri64**	4.234	XX	630	15	304720	1358	1297	1395	593	653	556	10.5	-6.8
**Picuri82**	36.614	XX	685	20	259754	1295	1277	1384	655	674	566	10,0	-7.2

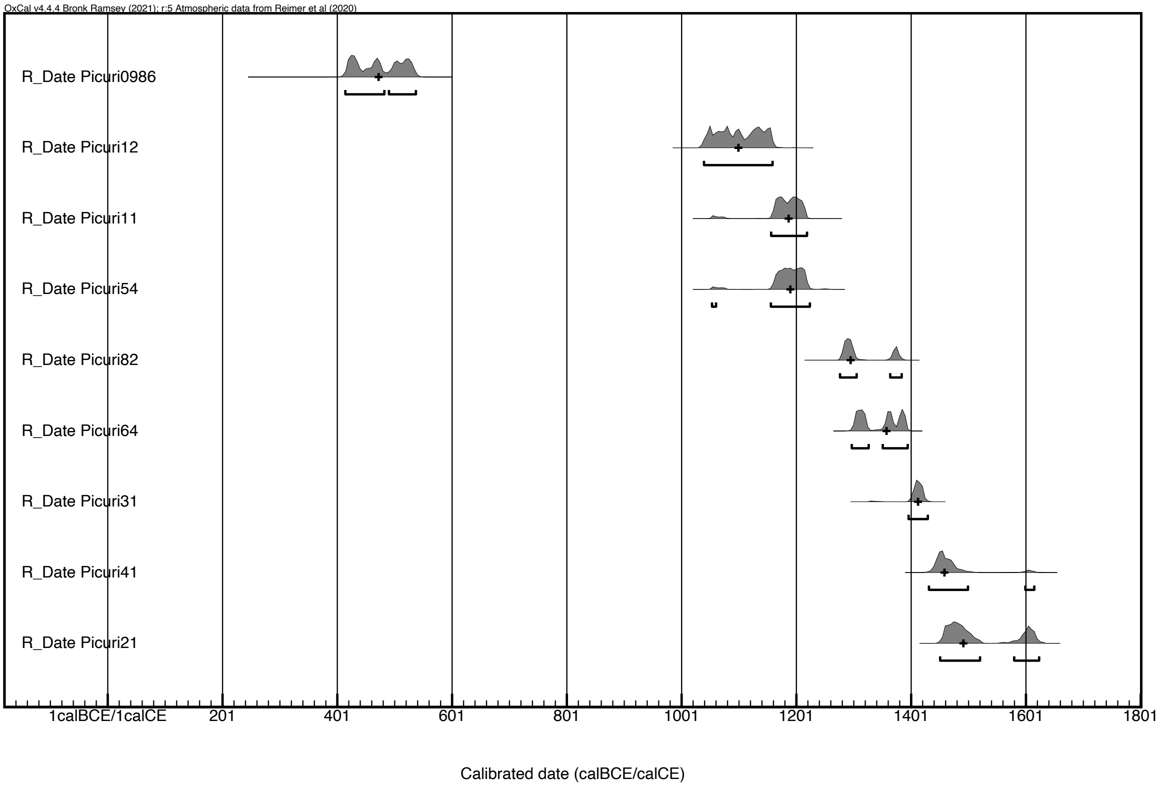

Supplemental Table S07: Direct radiocarbon dated ancient individuals from Picuris Pueblo.

3.3. Historical context of genetic studies in the North American Southwest

With the exception of a few preliminary studies surveying blood group prevalence[12–15] – including, quite remarkably, ones done on ancient mummified tissue in the 30s[16–18] – genetic studies of populations of the American Southwest are scarce. After the publication of Pääbo's successful recovery of DNA from Egyptian mummies in 1985[19], an attempt was made to obtain ancient DNA from mummies from Basketmaker and Ancestral Puebloan contexts from the area[20]. The study was purely exploratory and aimed only to evaluate DNA preservation in mummified tissue. Even though the results at the time were positive, those long fragments (>500kb) are now known to not be endogenous, but rather stem from present-day contamination[21,22]. Further PCR-based, ancient DNA attempts were made in the 90s and beginning of 2000s[23–26], but again, due to no reliable way of assessing the authenticity and source of the DNA molecules obtained, the results cannot be verified. To our knowledge, no Indigenous community was contacted at the time about the destructive intervention performed in those ancient individuals.

After the success of the application of novel molecular methods for population genetics – such as detection of mitochondrial DNA polymorphisms using restriction enzymes[27] and the resulting description of the four main maternal haplogroups found in Native Americans[28] – much interest existed for a thorough description of the variation found in North America. Turning our attention to the Southwest, initial studies showed haplogroup B to be the dominant lineage, peaking in Pueblo communities, followed by haplogroup C[29–32]. Haplogroup A, in turn, seemed to be very rare, except among Athabascan-speaking communities[31,32]. This is in stark contrast with Mesoamerica, where haplogroup A was found to be the dominant lineage, with a relatively low occurrence of haplogroup B[32,33]. However, those methods were restricted to control region variants, providing very shallow haplogroup definition, and were prone to misidentification due to reoccurring mutations on the hypervariable region or to noise stemming from poorly preserved samples.

Nevertheless, when reassessing the American Southwest data from [31] using current subhaplogroup knowledge, we can infer the presence of two of the haplogroups found in Picuris: B2a (defined by C16111T and G16483) in 4 out of 4 individuals from Zuni Pueblo, 1 individual from Jemez Pueblo and 1 Apache individual; and C1b11 (defined by C16295T) in 1 Apache. Repeating the same exercise in the data from [32] allows us to diagnose haplogroup B2a among the Akimel O'odham, Jemez Pueblo, Tarahumara, Tohono O'odham and Zuni Pueblo, and specifically haplogroup B2a4 among the Tarahumara, Jemez Pueblo and Zuni Pueblo. Haplogroup C1b11 is found on the Tohono O'odham.

Some studies were also done using diagnostic Y-chromosome markers. Due to its much more complex structure, size and copy number in the cell (in comparison to the mtDNA)[34], the resolution only allowed to differentiate between Native and non-Native lineages. However, due to the fact that Athabascan speakers carry a highly divergent Y-chromosome lineage (C3-P39/Z30536)[35], this allowed the detection of a direct northern contribution on the male gene pool among the Southern Athabascans, that is not found in any other population of the Southwest, such as Puebloans[36].

A historical abuse and misuse of biological material collected from communities in the US by researchers has fostered an environment of distrust between Indigenous people and academia. One of its most glaring examples is the Havasupai case, where material collected for diabetes research in 1989 was being used freely in 2003 for other studies – such as population genetics

and psychiatric disorders – without community consent[37]. Broad consent forms were used for justification of the legality of this kind of research, including consent for technologies that did not exist at the time, such as whole-genome sequencing. This has further eroded the fragile bond between communities and researchers, and have effectively halted further studies in the 21st century.

With the advancement of the ancient DNA field in the 2010s, the relationship between researchers and communities was again put under distress. Questions such as biocolonialism, consent and data ownership are significantly more complex when it comes to archaeological material, and which ethical standards to be followed is an active and heated debate[38–44]. While US legislation makes community consent a requirement for individuals with known cultural affiliation, the same is not true when that connection for some reason is not established. This led to the genomic processing of 9 individuals from Room 33 of Pueblo Bonito, Chaco Canyon, New Mexico, housed in the American Museum of Natural History[45] and has stirred a large controversy around its adequacy[2]. This study was able to establish that the burials in the room were all members of a matrilineal elite, a system described in historical records for almost all Puebloan communities and lengthily discussed in anthropological and archaeological literature in the region[46–48].

3.4. Linguistic context of Picuris Pueblo

The language spoken in Picuris is called *Northern Tiwa*, and it is part of a language family called Kiowa-Tanoan. Its closest extant relative is the language spoken at Taos Pueblo, that is 50 kilometres away from Picuris Pueblo. The literature is conflicting in how much mutual intelligibility there is, if any, between those languages[3,49]. The authors of this paper that are native Picuris speakers, however, report that there is a high level of understanding between them and Taos speakers, despite the pronunciation being very different for some words. This close relationship is supported by the fact that both communities have oral histories placing their origin in Pot Creek Pueblo, which was occupied contemporaneously with Picuris and Taos Pueblo until approximately 1320 CE. Oral traditions at Picuris Pueblo recognize that some of the inhabitants of Pot Creek Pueblo relocated to Picuris Pueblo after the depopulation of Pot Creek Pueblo.

Picuris, together with Taos, are referred to as 'Northern Tiwa' and alongside with 'Southern Tiwa' – the languages of Isleta Pueblo, Sandia Pueblo and Ysleta del Sur Pueblo – form the Tiwa languages. The meaning of the word 'Tiwa' is not clear, but it is likely a Southern Tiwa endonym, and the first Spanish chronists and maps name the area as 'Tiguex'. Additionally, most authors consider the extinct language spoken in Piro Pueblo also a Tiwa language (but see [50]), despite it being very poorly attested – its entire corpus being of a few place names and an 1860's translation of a catholic prayer[51,52].

The Tiwa languages, together with Towa (spoken at Jemez Pueblo) and the Tewa languages form a language group named Tanoan. Tewa is divided in Arizona Tewa (spoken in Hano, a Hopi Pueblo village) and Rio Grande Tewa (from Nambe Pueblo, Pojoaque Pueblo, San Ildefonso Pueblo, Ohkay Okingeh, Santa Clara Pueblo and Tesuque Pueblo). While the relationship between Tewa, Tiwa and Towa (henceforth, Tanoan) seem to have been clear even to the Spanish invaders (and the very early works on linguistics in the continent already place them together, e.g. [53]), it was only in 1910 that the relationship between Tanoan and Kiowa was proposed [54]. With further work in the following 50 years, this relationship was widely accepted[55–59]. The nature of this relationship can be traced to the long-term exchange and marriage relationships between the Kiowa – which historically has comprised communities of

largely mobile, bison-hunting peoples on the Southern Plains of the US – to the Tanoan sedentary, agricultural lifestyle. These contrasting economies were highly integrated in the "Plains-Pueblo Interaction System"[60] that flourished in the Northern Rio Grande and Southern Plains prior to European contact. These contrasts in lifestyle and economy in societies speaking related languages are found in other language families, such as in Uto-Aztecan[61], and stand witness to the large time depth of the cultural divergences within the Tanoan language family.

Despite significant advances in glottochronological analyses, including using Bayesian statistics and methods originally developed for genetic data (e.g. [62–64]), most scholars would agree that the best way to time linguistic divergence is reconstructing terms in its proto-language that can be archaeologically dated. One such example is the reconstruction of many words related to wheels, chariot or chariot-riding in Proto-Indo-European[65]. Similarly, proto-languages can be used to infer a group's homeland, by investigating the geographical distribution of plants and animals for which terms can be reconstructible.

Using this methodology, scholars have argued that reconstructed Proto-Kiowa-Tanoan and Proto-Tanoan provide insights into their divergence time and homelands[66,67]. The fact that the word for 'maize' is reconstructible, but not for 'squash' and 'beans', indicates that those languages split before maize arrived in the Southwest, but after squash and beans were introduced. This would place the split between 4,260 and 3,200 years ago. Instead, the split between the Tanoan languages (Towa and Tewa-Tiwa), occurred before the emergence of pottery in the region (1,700 years ago), as those languages do not have a cognate for it. Additionally, plant and animal species seem to suggest an origin of Proto-Kiowa-Tanoan in the upper San Juan river in Colorado, and a later movement of Tiwa speakers to the Rio Grande area.

3.5. Current archaeological models for the population history of northern Rio Grande

Current models regarding the origins of northern Rio Grande (NRG) populations, including their links to Chaco Canyon, based on archaeological and linguistic evidence. A summary can be found in **Supplemental Figure S1**.

Model 1 (Southern origins)

Under this scenario, northern Rio Grande (NRG) populations are derived from proto-Tewa-Tiwa speakers, who had been occupying the middle Rio Grande region since c. 600 CE, and who diverged into Tewa and Tiwa language groups during the 9th or 10th centuries. The ancestral Tewa remained in the Tewa Basin, while some ancestral Tiwa peoples moved north to found the Northern Tiwa populations (which include Picuris and Taos Pueblos), while other Tiwa-speakers stayed in the more southerly region to form the Southern Tiwa group. Various lines of evidence linking the Northern Tiwa to this divergence and dispersal include continuity in architectural features and layouts, as well as in ceramic styles and technologies.

Model 2 (Multi-ethnic origins)

A contrasting hypothesis proposes that NRG populations are descendants of a multi-ethnic enclave[68,69], that emerged c. 400-700 CE with agrarian peoples migrating from the La Plata region of southern Colorado south into the San Juan Basin/Chaco Canyon area. Around 800-850 CE, a second influx of groups migrated to the Chaco area from the Cíbola region to the south and west. This was soon followed by an influx of groups from the Dolores/Mesa Verde region (coincident with the large depopulation of that area starting ~880 CE). Following this rapid increase in population in the Chaco area there was a period of divergence and dispersal and it is argued – based on ceramic data[69] – that some groups made their way into the NRG,

arriving there soon after 900 CE (which marks the initial settlement of Picuris Pueblo). Craniometric analyses between NRG and Chaco Canyon also seem to support this hypothesis[68]. Although the impetus and timing for this move to the NRG are unknown, some scholars attribute its cause to differences in belief systems then-developing in Chaco[70].

Model 3 (Northern origins)
Finally, in contrast to hypotheses 1 and 2, others[71] have proposed that the proto-Tewa-Tiwa speakers emerged as distinct group in 9th-10th century CE in the Navajo Reservoir/Fruitland area in northwestern New Mexico. Soon thereafter it is argued these groups diverged linguistically and dispersed, with the ancestral Northern Tiwa migrating southeast into the NRG. The ancestral Tewa, on the other hand, remained in the Upper San Juan area, and only much later (the 13th and 14th centuries) expanded southeast into the Rio Grande valley[72]. Neither dispersal is presumed to have contributed significant populations to, or received populations from, the Chaco region.

There are several differences in these models. The first is the ultimate geographic source(s) of the Tewa / Northern Tiwa populations: Northern source populations are implicated in models 2 and 3; Southern sources are implicated in model 1. Importantly, the contribution from those different sources should implicate with the genetic affinity between populations in the northern Rio Grande region and Chaco Canyon. However, due to the complete lack of sampling in the region – the only data points are elite burials from Pueblo Bonito and Picuris Pueblo – we are not able to formally test any of those models.

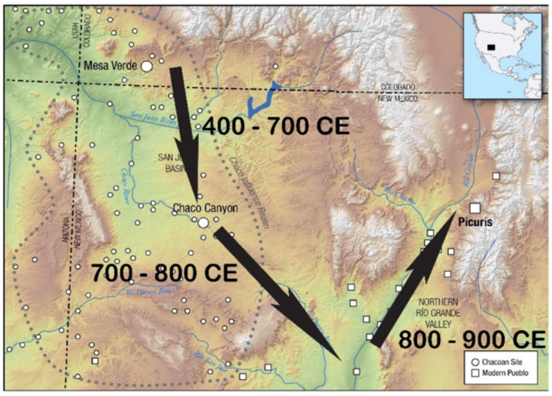

Model 1: Taos via Chaco, Southern Hypothesis (Boyer, Lakatos, Schillaci and others)

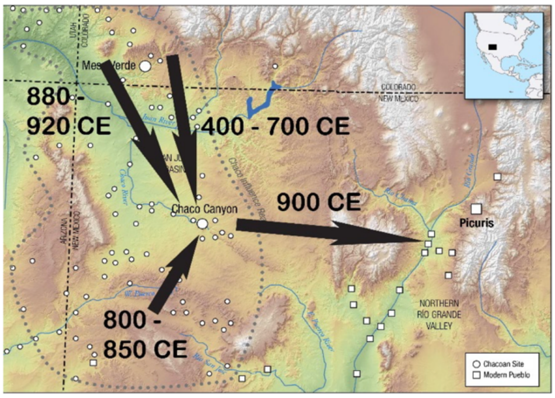

Model 2: Multiethnic Chaco (Schillaci and others)

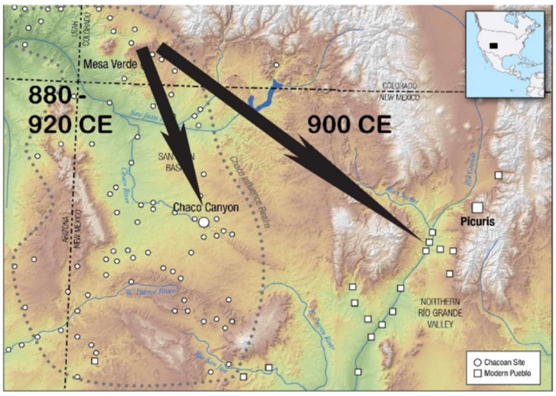

Model 3: Taos via Upper San Juan, Northern Hypothesis (Ortman , Cooper, others)

Supplemental Figure S01 Archaeological and linguistic models for the relationship between Chaco Canyon and Northern Rio Grande (NRG) region.

4. Sampling and laboratory procedures

4.1. Ancient individuals

In total, 35 ancient individuals excavated in early 1960s by Herbert Dick were housed in a locked room on the SMU-in-Taos campus located south of Picuris Pueblo. After two years of discussion between Eske Willerslev and tribal leadership at Picuris Pueblo (see **section S3**), 28 samples belonging to 22 individuals were selected by Picuris leadership and researchers based on criteria known to correlate with good DNA preservation, such as macroscopic integrity, colour and anatomy[73,74]. All work pre-amplification was done in dedicated ancient DNA clean laboratories at the Centre for GeoGenetics, GLOBE Institute, University of Copenhagen. Tooth and bone samples were first gently cleaned with a low speed sterile cutting disc to remove the outer layer and only then sampled at the histological regions that are richer in DNA (cementum and cortex, respectively[74]). In the case of petrous bones, the sampling was done at the area surrounding the otic capsule, which was cleaned using a round-shaped drilling utensil at low speed. All samples were then further crushed before lysis. In all cases, to reduce to a minimum the amount of destructive intervention performed in the material, all sampling was done with the aim of obtaining only between 50 and 100mg of bone powder, trying to leave as much area as possible untouched.

To further remove potential surface contaminants, samples were briefly pre-digested following[73] before being incubated at 37ºC in an EDTA-based buffer for 2-3 days. DNA was then extracted and purified using the protocol described in [75], but by making use of an optimised binding buffer described in [76]. To assess the authenticity of the extracted ancient DNA (*screening*), we built 34 double-stranded blunt-end libraries (following [77] with modifications from [78]) using 10µL of extracted DNA as input for shallow sequencing. Libraries were amplified and indexed using Illumina adapters, the concentration of libraries was estimated using qPCR and whole-genome sequencing was performed on an Illumina NovaSeq 6000 platform at the GeoGenetics Sequencing Core. Individuals displaying low level of contamination, endogenous DNA content higher than 0.5% and ancient DNA damage patterns[79] (**section S4.3**) were selected to undergo another round of library build and deeper sequencing. For this stage (*deep sequencing*), 23.1µL of each extract was first treated at 37ºC for 3 hours by a enzymatic cocktail consisting of Uracil DNA Glycosylase (UDG) and DNA glycosylase-lyase Endonuclease VIII ("USER enzyme") to remove characteristic ancient DNA damage created by cytosine deamination[80]. Those extracts were then built into 54 single-stranded libraries, following a combination of steps from [81–83], and showed an increase of library complexity of many fold. Those libraries were indexed and amplified using custom adapters from [83], its concentration estimated using qPCR and whole-genome sequenced at a Illumina NovaSeq 6000 platform at the GeoGenetics Sequencing Core.

After the conclusion of the project, all bone material, leftover bone powder, digested bone powder, DNA extracts, pre-amplified and amplified libraries were separated per individual and will be repatriated to Picuris Pueblo for reburial.

4.2. Present-day saliva samples

During one of the meetings between the research group in Copenhagen and Dallas and the tribal council of Picuris, the council suggested the inclusion of DNA from present-day members of the tribe to compare with the genetic results obtained from the ancient individuals. A collaboration agreement between the University of Copenhagen and the Picuris Tribal Nation was signed by both parties after evaluation by both the Universities' legal team and ethics committee and by Picuris Nation lawyers. The agreement includes, among other dispositions, the usage of these data exclusively for the means of this project or for replication

purposes, and the subsequent destruction of all DNA extracts and libraries obtained from present-day individuals after its conclusion.

Twenty-two saliva self-collection vials were shipped from Copenhagen to Picuris Pueblo. Sample collection was performed by Michael Adler and Richard Mermejo, tribal liaison to the project, Picuris Pueblo member and a co-author of this study. Participation was entirely voluntary, and individuals were informed on the nature of the study by the tribal liaison in both English and Northern Tiwa. Thirteen individuals volunteered to contribute with their biological sample after signing an informed consent form. Vials were labelled 1 to 22 prior to sampling, but were not used in sequence. Therefore, present-day individuals were labelled *mPicuri* plus the vial number (1, 3, 4, 7 to 12, 15, 16, 21, 22). In order to allow volunteers to be removed from the project at any time, in case they no longer wish to participate, samples were not fully anonymised. The list that identifies volunteers by name and their corresponding sample number is kept by the Picuris Tribal Secretary, at the tribal office at Picuris Pueblo. No identifying information was made available to the research group in Copenhagen or Dallas, with the sole exception that the individual who contributed the saliva was an enrolled member of the Picuris Tribal Nation.

Extractions were conducted using Qiagen DNeasy Blood & Tissue Kit with a slightly different protocol. Sample concentrations were normalized based on Qubit measurements, and genomic DNA was fragmented with Covaris. The preparation of DNA libraries was done using KAPA HiFi HotStart Uracil+ ReadyMix Mastermix for the indexing PCR, along with Illumina TruSeq DNA UD indices. Following library build and purification, quality control was conducted using Fragment Analyzer. Libraries were equimolarly pooled for sequencing, with the addition of 1µL of the negative control. The sequencing was carried on a NovaSeq 6000 S4 flow cell (paired-end, 2x100bp) at the GeoGenetics Sequencing Core.

5. Mapping, post-processing and ancient DNA authentication

Raw sequencing data was demultiplexed used Illumina's BCL Convert software, allowing one mismatch in the index. Reads had their adapters trimmed using AdapterRemoval[84] v2.3.2, with a minimum length threshold of 30 bp and overlapping reads collapsed, by specifying options --minlength 30, --collapse-conservatively and by providing the appropriate adapter sequence, depending on if it was a double- or single-stranded library. Collapsed and paired-end reads were mapped to genome assembly GRCh38 using bwa aln v. 0.7.17[85], and reads from the same library were duplicate marked using Picard v.2.25.0 MarkDuplicates. Depth of coverage and average read length was estimated using an in-house pysam-based script[86,87] by estimating the total length of mapped reads and dividing it by 1) the sum of the total length of chromosomes 1 to 22 for depth of coverage or 2) total number of mapped reads for the average read length. When estimating the coverage of chromosome Y, we restrict the calculation to reads mapped in the short-read accessible region defined in[88] and divide by the length of this mappable tract.

To assess the authenticity of the DNA obtained in the extractions, we evaluated two hallmarks of ancient DNA, the short average read length and post-mortem fragmentation and misincorporation patterns estimated using mapDamage 2.0[89] and bam2prof[90]. Modern-day contamination estimates were generated by measuring heterozygosity on the mitochondrial genome with ContamMix[91] in comparison to a set of possible worldwide contaminants. In the case of male individuals, this was also performed on the X-chromosome (following [92]), using ANGSD v. 0.931[93] new implementation described in [94]. All analyses were performed on reads with mapping quality ≥ 30 and bases with quality ≥ 20. Individuals with the lower bound of the mitochondrial contamination estimate above 5% (if mtDNA coverage > 10x) and X-

chromosome estimate above 2% were flagged but kept in the analyses. Individuals where the mean estimates exceeded 5% were deemed contaminated and excluded from downstream analyses.

We provide results and summary statistics both per library and individual in **Supplemental Table 01-02**. Naming convention for sequencing data in this project is individual, code, extraction, library, method (separated by underline), followed by either "E" or "U", denoting non-USER or USER-treated libraries. Libraries with the same name underwent the same PCR reaction and therefore duplicates should be identified and removed jointly. Results for present-day members of Picuris Pueblo can be found in **Supplemental Table 03.**

6. Chromosomal sex inference

The genetic sex of individuals can be inferred by comparing the depth of coverage between autosomes and sex chromosomes in many ways. Because of its relative small size and regions unmappable using short-read technology[34], the estimated coverage of the Y-chromosome can greatly vary in low-depth genomes, particularly in the presence of small amount of contamination. We therefore diagnosed the genetic sex of the individuals using the read dosage of the X-chromosomes in relation to the autosomes, comparing it to a function describing the expected ratio for each sex[95]. In order to investigate the presence of potential sex chromosome aneuploidies, we plotted the ratio of Y, X and autosomes, and found all individuals to be either XX or XY (**Supplemental Figure S02**).

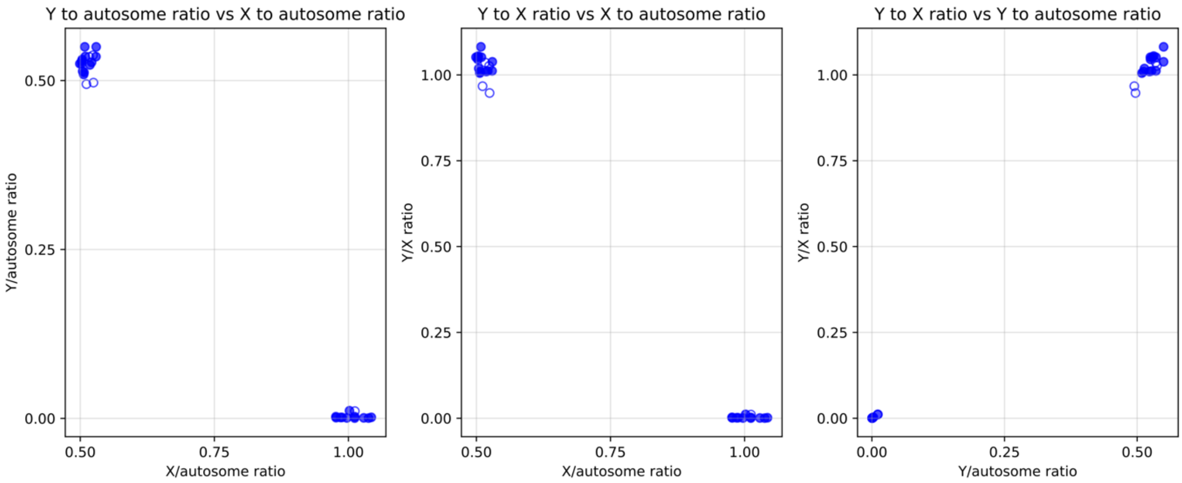

Supplemental Figure S02 Ratio between autosomes, X- and Y-chromosomes of ancient individuals from Picuris Pueblo. Low-coverage individuals are represented as unfilled circles.

7. Mitochondrial DNA analyses

To investigate the relationship between Picuris maternal lineages with other populations in the Americas, we turned our attention to the mitochondrial DNA of the new individuals. We estimated depth of coverage using samtools[86] version 1.3.1 "depth" function, and use its "mpileup" module to generate consensus sequences by requiring minimum 5 reads and over 70% frequency on the population of non-clonal reads in a locus to call a base. We estimated the completeness of the consensus by counting the number of positions with a call and dividing it by the length of the mitochondrial reference. Haplogroup assignment was done using Haplogrep 3[96]. All individuals belonged to Native American haplogroups, including all present-day Picuris Pueblo members. Ancient Picuris were of haplogroups A2f, B2, B2a, B2f and C1b11, while present-day individuals belonged to A2a, B2a, B2s, B2y and C1b11.

We then collected all mitogenomes from ancient and present-day individuals from the literature belonging to haplogroups A2f, B2a, B2f, B2y and C1b11 from[45,97–118], and aligned them to the mitochondrial reference sequence (rCRS) using mafft[119] --add and --keep-length options. Positions 309-315, 515-522, 16182-16183, 16193 and 16519 were hard masked using bedtools[120] after [121]. A maximum-likelihood tree of each haplogroup was built using raxml-ng v.0.8.1[122] using a bootstopping strategy[123] to determine the necessary number of Felsenstein replicates[124]. Near-zero branches were collapsed and annotated trees can be found in (**Extended Data Figure 02, Supplemental Figure S03-S06**). For haplogroup B2a, B2y and C1b11 we also used BEAST v2.6[125], using a birth-death model including ancient DNA individual ages as tip dates. We performed 200,000,000 steps with sampling every 10,000 generations, with a 5% burn-in during tree sampling (**Supplemental Figure S07-S09**).

Haplogroup A2f is mainly restricted to North America, and divided in three branches (**Supplemental Figure S03**). A2f1 seems to have a more septentrional distribution, being present in the Cree and Mi'kmaq, Algonquin groups from Canada. A2f2 and A2f3 instead are found in Mexican-Americans and an individual from Texas, hinting at a more southerly area of occurrence. Five unrelated ancient individuals from Picuris have haplogroup A2f, but do not belong to neither of those three previously described clades, instead forming their own branch.

Haplogroup B2a is the most common Indigenous haplogroup of US Southwest and North Mexico **(Supplemental Figure S04, S07)**. It has at least 5 branches, that are found in present-day and ancient individuals from the US states of California, Nevada, Utah, Texas, Colorado, New Mexico and Arizona, as well as Mexico states of Baja California, Sonora, Chihuahua, Durango, Jalisco and Sinaloa. One individual from Jemez Pueblo is B2a1, and one Akimel O'odham from Northern Mexico is B2a5. Ancient and present-day Picuris individuals belong to B2a2 (4), B2a2x2 (1) and B2a4a (5). One low coverage ancient Picuris is also B2a, but its coverage is not enough for further definition downstream.

Haplogroup B2f is a relatively rare haplogroup, present only in US and Mexico (**Supplemental Figure S05)**. Among ancient individuals, it occurs both in Picuris and in the Casas Grandes culture from Northern Mexico, which some researchers claim to have strong ties with Ancestral Puebloan culture.

Haplogroup B2y is a widespread haplogroup, being present in the US, Peruvian Andes, North Argentina and the Brazilian Amazon **(Extended Data Figure 02, Supplemental Figure S08)**. Subhaplogroup B2y1, however, seem to be restricted to North America. In an ancient DNA study conducted in ancient individuals from Pueblo Bonito's Room 33 (Chaco Canyon) housed in the American Museum of Natural History in New York, Kennett and collaborators identified

that all individuals buried there belonged to the same matrilineage[45], downstream of B2y1. Strikingly, one present-day individual from Picuris Pueblo of haplogroup B2y1 carries all variants present in the Pueblo Bonito matrilineage, and forms a clade with the Pueblo Bonito haplotype with 100% bootstrap support in the maximum-likelihood analysis and 1 posterior probability in the Bayesian phylogeny. Using BEAST, we estimate this branch to have formed around 2,800 BP. Alongside the autosome results, this result represents one of the strongest evidence of population continuity between Ancestral Puebloan and present-day Puebloans.

Two ancient Picuris fall basal to haplogroup B2, not belonging to any known clades.

Finally, haplogroup C1b11 also seem to be spatially restrict to US and Mexico **(Supplemental Figure S06, S09)**. Ancient and present-day Picuris form their own branch downstream of subhaplogroup C1b11x1. In what is likely an effect of the population collapse following European arrival, all but one of the present-day Akimel O'odham from North Mexico from [117] belong to C1b11x2.

One of the publicly available mitogenomes stems from an individual who identifies itself as a member of the Navajo (*Diné*) nation. As the source of this sequence is a publication post-2002 (when the Navajo nation has issued a moratorium on all genetic research[126]), either the consent predates the moratorium or the individual has claimed this ancestry for its maternal lineage. In respect to the wish of the Navajo nation to not be part of any kind of genetic research, we have erased the population label of this individual.

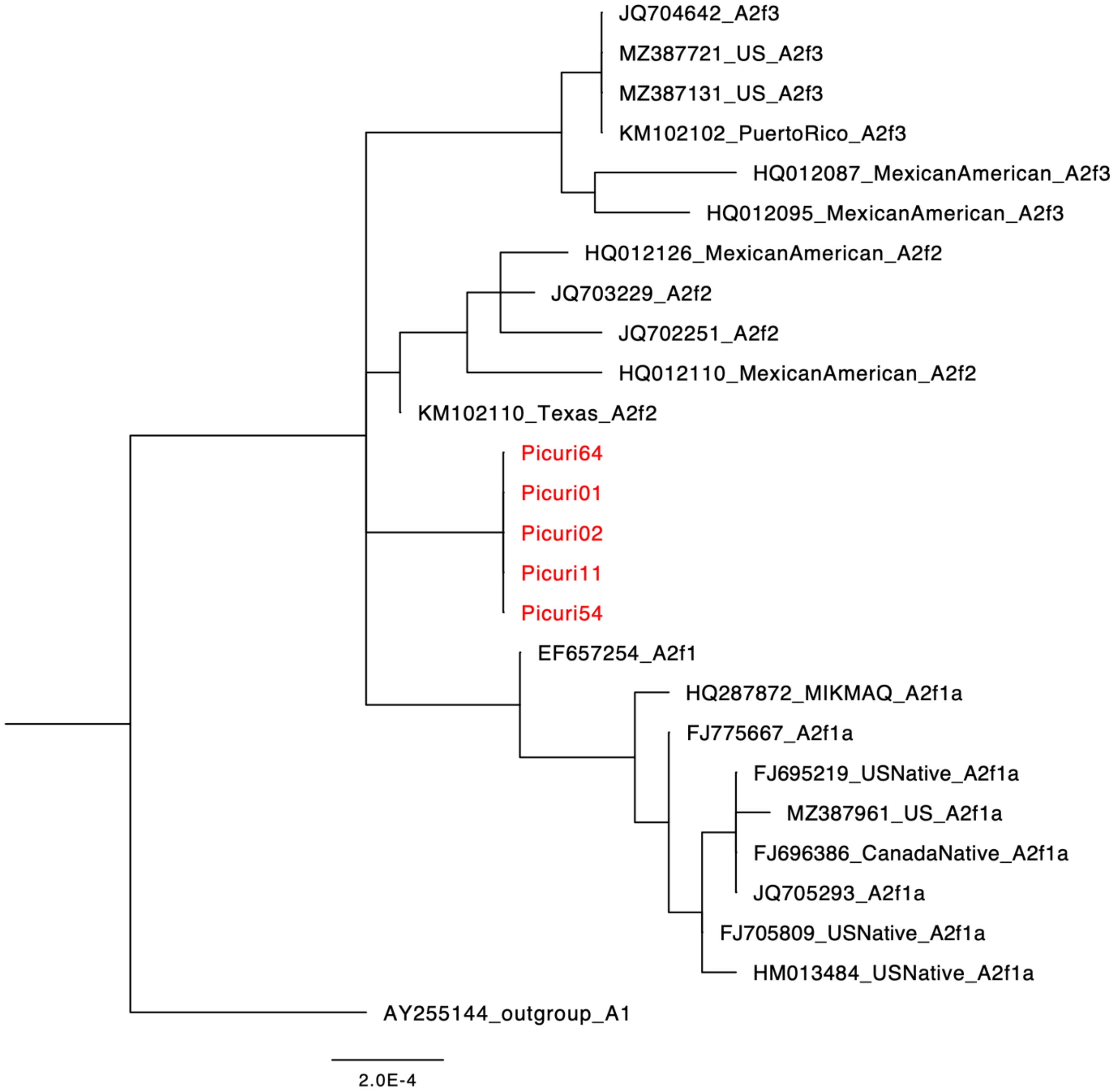

Supplemental Figure S03 Maximum-likelihood tree of mitochondrial haplogroup A2f. Present-day literature mitogenomes are in black, ancient Picuris in red and all other ancient mitogenomes in orange. Near-zero length branches were collapsed for convenience.

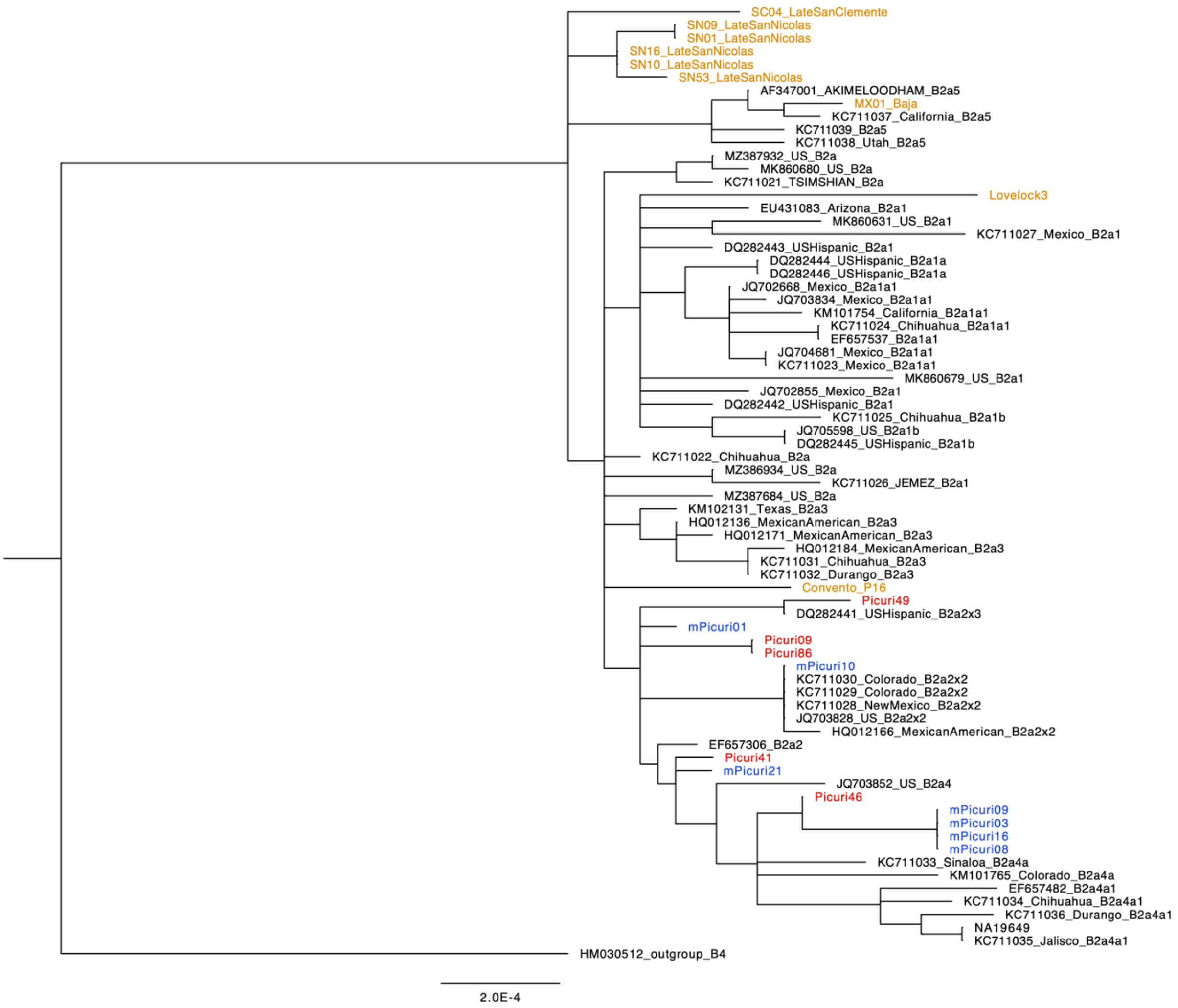

Supplemental Figure S04 Maximum-likelihood tree of mitochondrial haplogroup B2a. Present-day literature mitogenomes are in black, present-day Picuris in blue, ancient Picuris in red and all other ancient mitogenomes in orange. Near-zero length branches were collapsed for convenience.

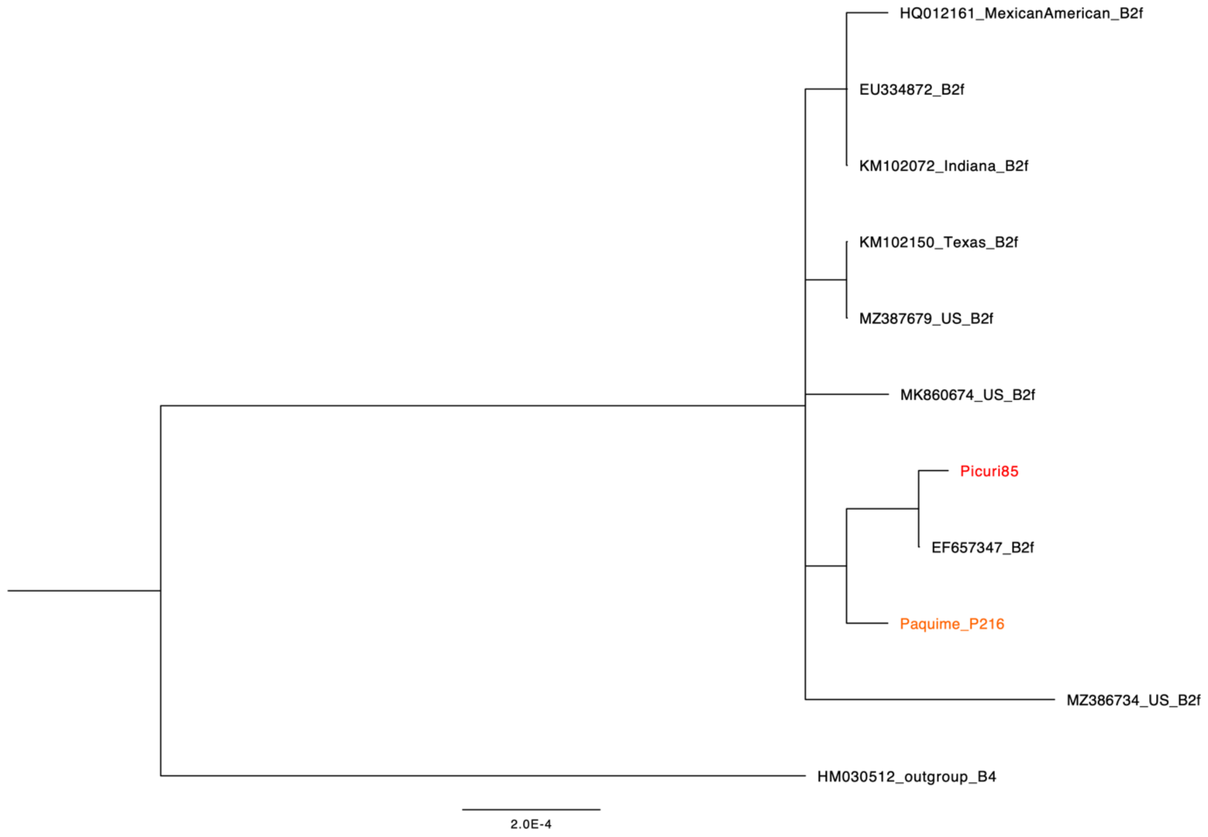

Supplemental Figure S05 Maximum-likelihood tree of mitochondrial haplogroup B2f. Present-day literature mitogenomes are in black, ancient Picuris in red and all other ancient mitogenomes in orange. Near-zero length branches were collapsed for convenience.

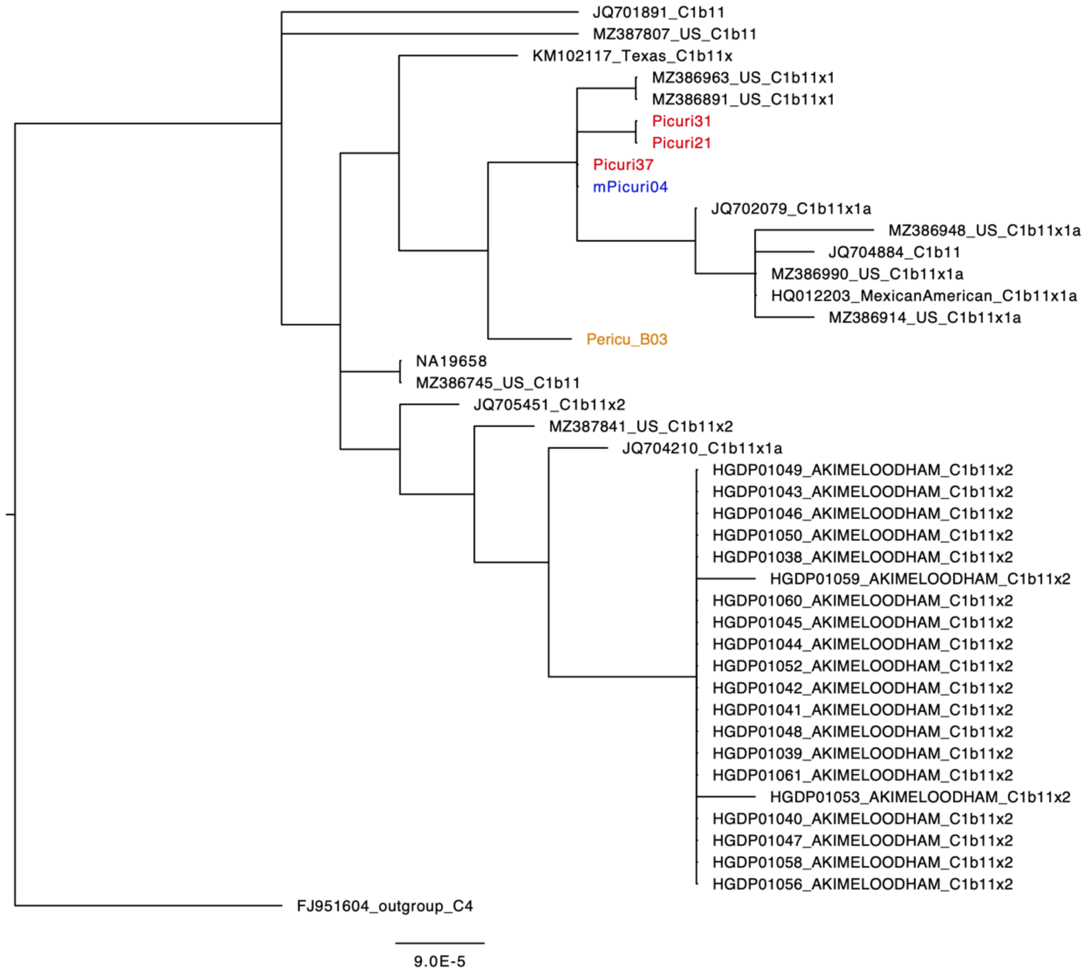

Supplemental Figure S06 Maximum-likelihood tree of mitochondrial haplogroup C1b11. Present-day literature mitogenomes are in black, present-day Picuris in blue, ancient Picuris in red and all other ancient mitogenomes in orange. Near-zero length branches were collapsed for convenience.

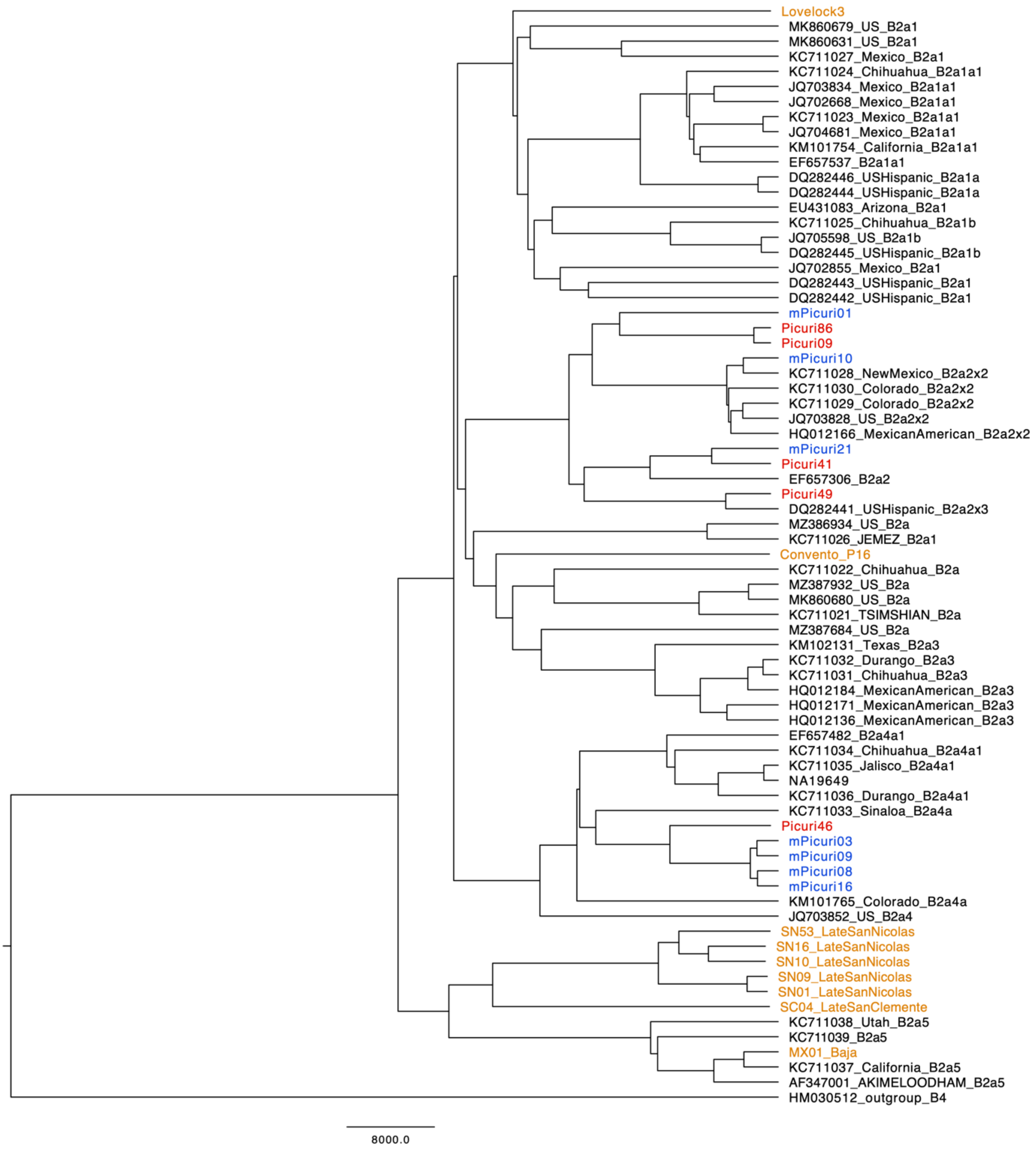

Supplemental Figure S07 Bayesian tree of mitochondrial haplogroup B2a. Present-day literature mitogenomes are in black, present-day Picuris in blue, ancient Picuris in red and all other ancient mitogenomes in orange.

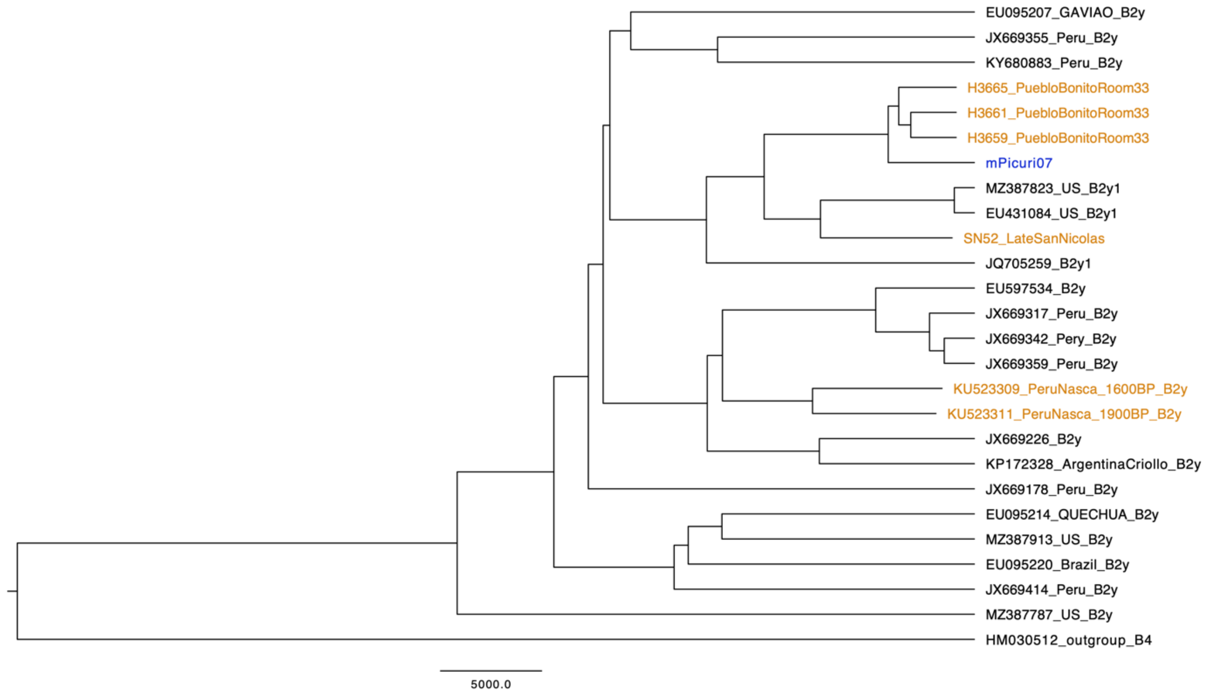

Supplemental Figure S08 Bayesian tree of mitochondrial haplogroup B2y. Present-day literature mitogenomes are in black, present-day Picuris in blue, ancient Picuris in red and all other ancient mitogenomes in orange.

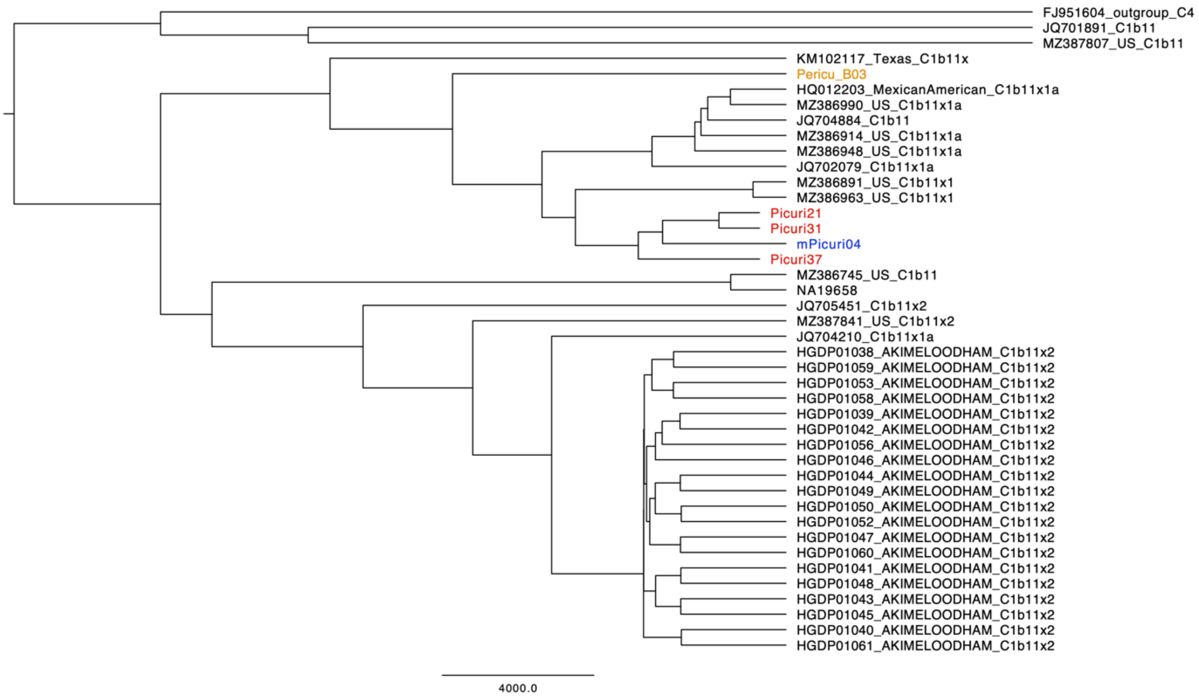

Supplemental Figure S09 Bayesian tree of mitochondrial haplogroup C1b11. Present-day literature mitogenomes are in black, present-day Picuris in blue, ancient Picuris in red and all other ancient mitogenomes in orange.

8. Y-chromosome analysis

We used bcftools v.1.17[127] "mpileup" and "call" function on the 10 Mb single-copy short-read callable region defined in [88] of the Y-chromosome to call genotypes, and further excluded triallelic locus, indels and variants called in less than 95% of non-clonal reads. Haplogroups were then called by matching ancestral and derived calls to ISOGG 2019-2020 and other private databases, using a script that generates root-to-tip haplogroup paths, sorted by the number of supporting variants[128]. Results can be found in **(Supplemental Table 02-03, Extended Data Figure 03)**.

All ancient Picuris with sufficient coverage belong to exclusively Indigenous American haplogroups Q-M3 and Q-CTS1780[35,129]. Eight out of thirteen present-day Picuris carry Y-chromosomes, and despite the widespread sex-bias in post-colonial admixture in the Americas[130], the majority of them are of Indigenous American haplogroups (5/8). Similarly with the mitochondrial results, the characteristic paternal lineages of Picuris Pueblo are genetically closer to other North Mexicans populations, such as the Akimel O'odham and Mexicans from Los Angeles from 1000 Genomes. We found that many of the Y-chromosomes among present-day Picuris to share plenty of undescribed variants, likely due to the population bottleneck post European arrival.

In respect to Picuris' wish to not have their data included in any commercial database, we place ancient and present-day Picuris in the Y-chromosome phylogeny, but do not list variants private to them.

Despite over 200 years of contact between Puebloans and Spaniards during Spanish colonization of the US Southwest, and historical figures known to be of mixed Picuris and Spanish descent (such as Luis Tupatú, one of the leaders of 1680's Pueblo Revolt), European subhaplogroups among Picuris are typically British or Northern European, such as R1b-L21 and I1a-Z58.

9. Dataset curation

9.1. Whole genome dataset

9.1.1. Remapping

To compare the newly generated shotgun data, we assembled all available present-day and ancient genomes from Far East Asia and the Americas for imputation[114,115,117,131–149], also including representative populations from Europe, East Asia and Oceania. Most of the ancient genomes were mapped to genome reference GRCh37, and therefore we remapped them to reference GRCh38 and re-assessed them for patterns such as ancient DNA damage, read length and mitochondrial and X-chromosome contamination, using identical settings as for ancient Picuris (**section S5**). We also use the same contamination thresholds for inclusion/exclusion of data. Most of the present-day individuals, in turn, were available in GRCh38. Therefore, we reproduced the steps used in the literature[117] to remap the few present-day genomes only available in GRCh37. Only genomes passing quality control and with depth of coverage above 0.3x were considered in the following steps. The full list of shotgun genomes included and their depth of coverage, average read length, damage pattern and contamination estimates is available at **(Supplemental Table 04)**.

9.1.2. Imputation and phasing

To generate genotype calls and genotype likelihoods, we used bcftools v. 1.17[127] commands *mpileup* (options "-I -E -a 'FORMAT/DP' --ignore-RG") followed by *call* (options "-Aim -C alleles") on a set of 44,136,290 variable sites (henceforth "1000 Genomes positions")[128,150,151] filtering for mapping quality above 30. The resulting files were used as input for GLIMPSE v1.1.1[152] to slice the chromosomes into 2 Mb chunks (plus a 200 kb buffer region) and to perform the imputation using 1000 Genomes as the reference panel. The chunks were then stitched back together using GLIMPSE_ligate and annotated using bcftools. Individuals displaying an average genotype probability lower than 0.98 were excluded from downstream analyses. This resulted in a final imputed dataset of 835 ancient and present-day genomes passing all filters, 590 of which stem from Siberia and East Asia (142) or the Americas (448).

On the variant level, we performed the following filters for all downstream analyses: (1) INFO score above 0.8[150,152] and (2) exclusion of variants not in the GRCh38 strict accessibility mask. We also apply filters for minimum allele frequency in the reference panel, as imputation has a significant lower performance for rare variants. Therefore, we restricted our analyses exclusively to non-rare variants (>1%), applying either a minimum 1% or 5% threshold for inclusion.

Additionally, we imputed all individuals from the Americas with publicly available capture hybridisation technology[45,153,143,154–159] using the same parameters and quality thresholds, except that we required depth of coverage at targeted sites to be above 1.5x. While this amounted to 141 individuals, only 8 stem from mainland North America. We also included data from a recent paper on North America[160], but in this case as pseudohaploid genotypes. The three previously genotyped individuals from Pueblo Bonito were at too low coverage for imputation, so they were also included as pseudo-haploid genotypes.

9.1.3. Local ancestry and masking of non-Indigenous American segments

As Y-chromosome analyses suggest some level of European admixture in present-day members of Picuris Pueblo, we attempted to identify regions of non-Indigenous American ancestry for masking for subsequent allele-frequency based analyses. We first ran an unsupervised ADMIXTURE analysis[161,162] with K equals to 3, to estimate general ancestry proportions, and confirmed some individuals to carry non-Indigenous American ancestry.

Using the imputed diploid genotypes from Picuris and a reference set of Western African, Western European and unadmixed Indigenous American individuals from both North and South America, we ran RFMIX v2.03-r0[163] with default parameters. We then used an in-house python script to generate a bed file containing only regions where both haplotypes were estimated to be of Indigenous ancestry with 0.99 probability, and set all other regions to missing in the vcf using bedtools intersect[120]. Both analyses were highly congruent, and the majority of individuals were found to have more than 80% Indigenous ancestry after diploid masking. We note that this is not an ancestry proportion, but rather a much stricter cut off. A notable exception and example of this is mPicuri15, which despite being estimated to be of 48 and 52 percent of Indigenous American and European ancestry, respectively, was almost completely masked out (~97.7%). This high proportion of ancestry 'heterozygous' sites likely represents a recent, possibly first generation, European ancestor.

9.2. Array-based dataset
Due to our focus in understanding the relationship between Picuris and other populations from the North American continent, we used the Illumina-chip genotype dataset assembled in [131] (including data from[136,145,146,164,165]), which corresponds to 2,537 individuals typed at 199,295 SNP sites. It includes 67 populations from the American continent, and is by far the most comprehensive panel for North American variation. We further enriched this panel with other 233 North and South American individuals stemming from 16 populations typed using similar Illumina technology from[166,167]. Overlap and merging was performed using plink v1.90[168], and plink files were lifted over from genome assembly 37 to 38 using UCSC LiftOver and a custom script. After filtering out sites that now mapped to decoys or locus where all worldwide individuals are monomorphic, we obtained an extended array-based panel covering 172,863 SNPs. Finally, we added to the panel the imputed diploid genotypes of all present-day and ancient whole-genome shotgun data from the literature, all individuals from the 1000 Genomes project, as well as all capture hybridisation data, which have an almost complete site overlap with the used array. This amounted to a dataset with over 300 different populations stemming from the American continent and more than 5,500 individuals **(Supplemental Table 04, 05, 06**).

10. Principal Component Analysis (PCA) and genetic clustering
To visualise the placement of Picuris individuals in the broad worldwide and American genetic landscape, we performed Principal Component Analysis (PCA)[169] using plink v1.90[168] using the diploid genotypes of individuals of our array dataset as input. We performed three iterations of this analyses, first including all individuals in our dataset (**Supplemental Figure S10**), then restricting to individuals from Far East Asia and the Americas (**Supplemental Figure S11**) and finally by only including individuals from North America (excluding Greenland) (**Supplemental Figure S12**). In order to identify potential intra-population outliers, we then used a model-based clustering method[161,162] on the dataset of North American individuals to identify major genetic groupings. We used plink v1.90[168] to prune the dataset to markers in high LD, and removed individuals with more than 70% missing data. We run 10 replicates of K from 2 to 10, and found the lowest cross-validation error at K=3 **(Supplemental Figure S13-S14)**.

Two populations were found to harbour genetic outliers with significant deviation in both MDS and ADMIXTURE: Southern_Athabascans_1 (1 out of 4) and Northern_Athabascans_1 (2 out of 8). After conducting formal "treeness" tests (**section S11**) we found those outliers to harbour significant additional ancestry, and therefore we re-labelled those individuals to avoid biasing the population frequencies.

In all PCA iterations, we found ancient and present-day individuals from Picuris Pueblo to be relatively homogeneous, and co-occur in PCA space with other populations from North Mexico and Southwestern US in general and with individuals from Pueblo Bonito[45] in particular, in line with allele-frequency based results (below). When restricting to North American populations and excluding Palaeo- and Neo-Inuits, this signal is even clearer, and Puebloan individuals occupy an intermediate position between Northern Native American (NNA) and Southern Native American (SNA) populations, but closer to SNA.

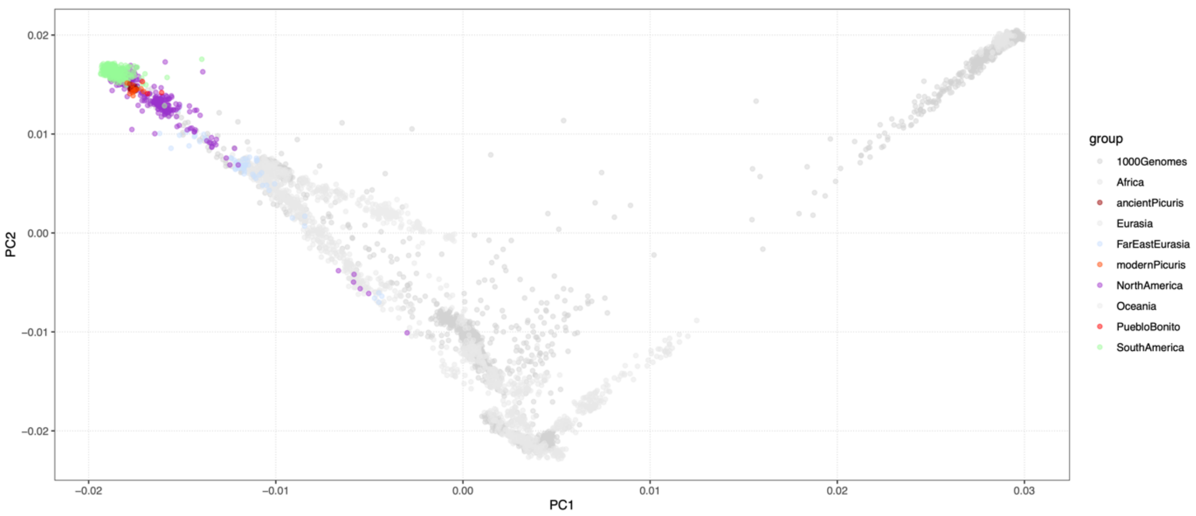

Supplemental Figure S10 Principal component analysis (PCA) of a set of worldwide present-day and ancient individuals.

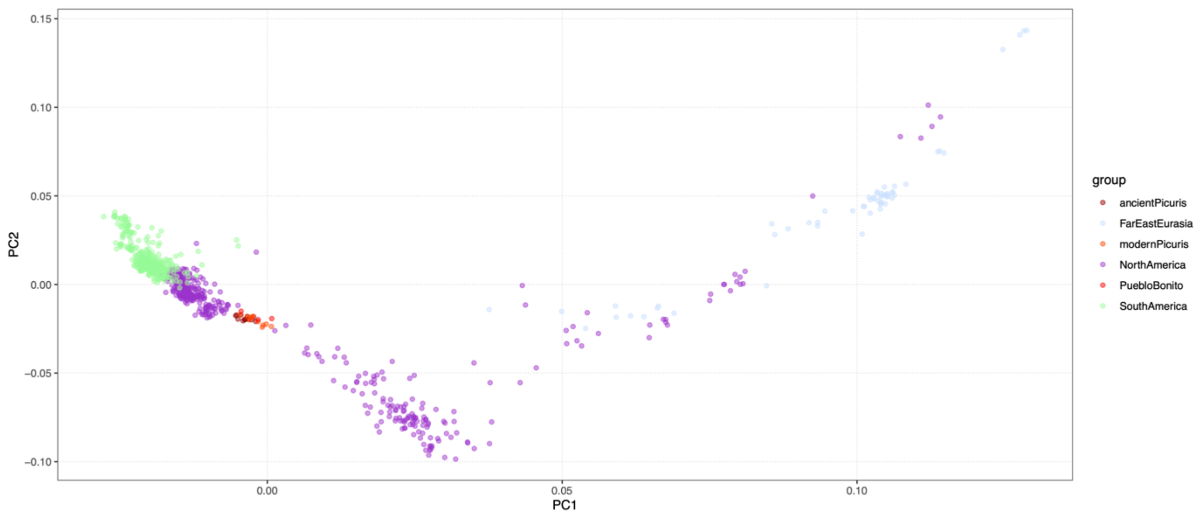

Supplemental Figure S11 Principal component analysis (PCA) excluding individuals not from Far East Asia or the American continent.

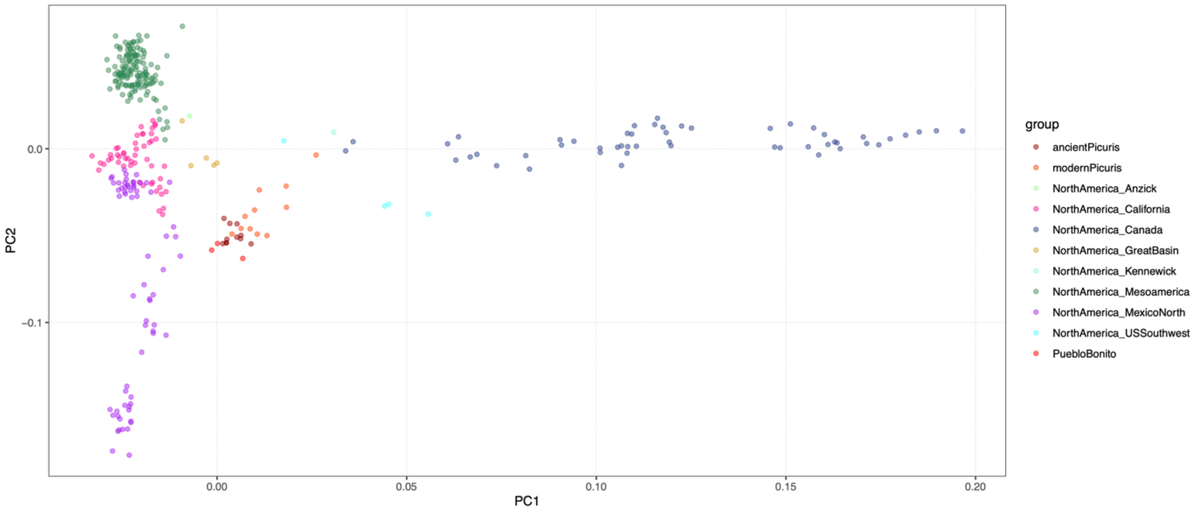

Supplemental Figure S12 Principal component analysis (PCA) only including genotyped individuals from North America.

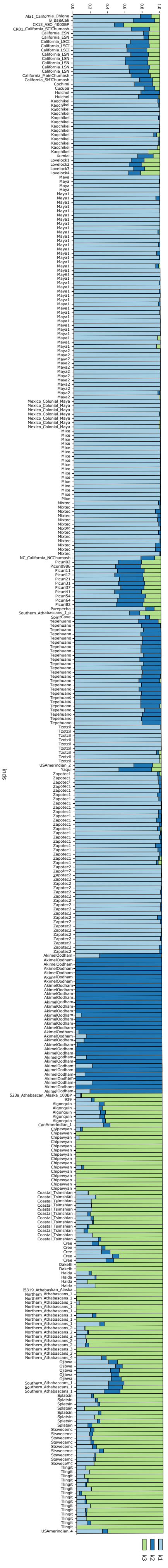

0.0
0.2
0.4
0.6
0.8
1.0
inds
k1
k2
k3

Supplemental Figure S13 Admixture analyses (K=3), only including individuals with less than 70% missing data.

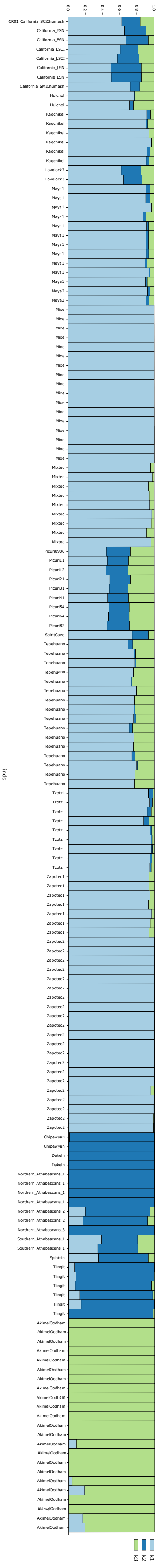

0.0
0.2
0.4
0.6
0.8
1.0
inds
CR01_California_SCIChumash
California_ESN
California_ESN
California_LSC1
California_LSC1
California_LSN
California_LSN
California_SMIChumash
Huichol
Huichol
Kaqchikel
Kaqchikel
Kaqchikel
Kaqchikel
Kaqchikel
Kaqchikel
Lovelock2
Lovelock3
Maya1
Maya1
Maya1
Maya1
Maya1
Maya1
Maya1
Maya1
Maya1
Maya1
Maya1
Maya2
Maya2
Mixe
Mixe
Mixe
Mixe
Mixe
Mixe
Mixe
Mixe
Mixe
Mixe
Mixe
Mixe
Mixe
Mixe
Mixe
Mixe
Mixe
Mixtec
Mixtec
Mixtec
Mixtec
Mixtec
Mixtec
Mixtec
Mixtec
Mixtec
Picuri0986
Picuri11
Picuri12
Picuri21
Picuri31
Picuri41
Picuri54
Picuri64
Picuri82
SpiritCave
Tepehuano
Tepehuano
Tepehuano
Tepehuano
Tepehuano
Tepehuano
Tepehuano
Tepehuano
Tepehuano
Tepehuano
Tepehuano
Tepehuano
Tepehuano
Tepehuano
Tepehuano
Tepehuano
Tzotzil
Tzotzil
Tzotzil
Tzotzil
Tzotzil
Tzotzil
Tzotzil
Tzotzil
Tzotzil
Zapotec1
Zapotec1
Zapotec1
Zapotec1
Zapotec1
Zapotec1
Zapotec1
Zapotec2
Zapotec2
Zapotec2
Zapotec2
Zapotec2
Zapotec2
Zapotec2
Zapotec2
Zapotec2
Zapotec2
Zapotec2
Zapotec2
Zapotec2
Zapotec2
Zapotec2
Zapotec2
Zapotec2
Zapotec2
Zapotec2
Zapotec2
Zapotec2
Chipewyan
Chipewyan
Dakelh
Dakelh
Northern_Athabascans_1
Northern_Athabascans_1
Northern_Athabascans_1
Northern_Athabascans_1
Northern_Athabascans_2
Northern_Athabascans_2
Northern_Athabascans_3
Southern_Athabascans_1
Southern_Athabascans_1
Splatsin
Tlingit
Tlingit
Tlingit
Tlingit
Tlingit
Tlingit
AkimelOodham
AkimelOodham
AkimelOodham
AkimelOodham
AkimelOodham
AkimelOodham
AkimelOodham
AkimelOodham
AkimelOodham
AkimelOodham
AkimelOodham
AkimelOodham
AkimelOodham
AkimelOodham
AkimelOodham
AkimelOodham
AkimelOodham
AkimelOodham
AkimelOodham
AkimelOodham
AkimelOodham
AkimelOodham
AkimelOodham
k1
k2
k3

Supplemental Figure S14 Admixture analyses (K=3), only including individuals with less than 10% missing data.

11. *f*-statistics

Two related allele-frequency-based methods were used to investigate the relationship between Picuris and other populations with available array data: outgroup *f3*-statistics and *D*-statistics[170]. For a "population" consisting of a single diploid individual, there are only three possible allele frequencies in a single locus (0, 0.5 or 1), thus largely reducing the statistical power for discriminating fine structure between populations. However, when individuals have 1) similar ancestry and 2) are roughly contemporary, it is reasonable to pool those individuals and calculate more precise allele frequencies. To test if assumption 1) is true, we tested whether any two individuals of a population formed a clade to the exclusion of other worldwide populations. We used FrAnTK[171] to iterate over all individuals of the same population in the form *D(individual1, individual2, pop, Yorubas)* where "pop" represents all other Native American individuals plus French, Han and Papuans. When *D* is not significantly different from zero ($|Z| < 3.3$), then the test cannot reject the cladality of the two individuals, and therefore we proceed to pool those individuals and jointly estimating their allele frequencies.

11.1. outgroup *f*3-statistics

We performed 'outgroup' *f*3-statistics, which is a special case of the *f*3-statistics where an outgroup is used as a target population. This statistic measure the shared drift between the two non-target populations, which is higher if they have a close genetic relationship, as shared drift correlates with shared population history. We therefore computed all possible statistics of the form *f3(ancientPicurisPueblo, pop, Yorubas)*, *f3(modernPicurisPueblo, pop, Yorubas)* and *f3(PuebloBonitoRoom33, pop, Yorubas)* where 'pop' represents all other Native American populations in the panel. We did this twice, first restricting to the Illumina-chip array positions (**Supplemental Figure S15, S16, S17**) and then to a subset of individuals covered in a larger number of sites ('1240K') (**Supplemental Figure S18, S19, S20, S21**). In this second case, we also run the analysis involving 'PuebloBonitoRoom33' restricting to transversion polymorphisms, as the individuals are at very low coverage and are not USER-treated.

In all cases, we found past and present Picuris Pueblo to be closer to each other than any other sampled population, with individuals from Pueblo Bonito having the second highest value in both cases. Instead, when testing Pueblo Bonito, ancient and present-day Picuris Pueblo were the populations with the highest value, showcasing the high genetic similarity between those three populations and the local continuity in both Picuris Pueblo and the North American Southwest.

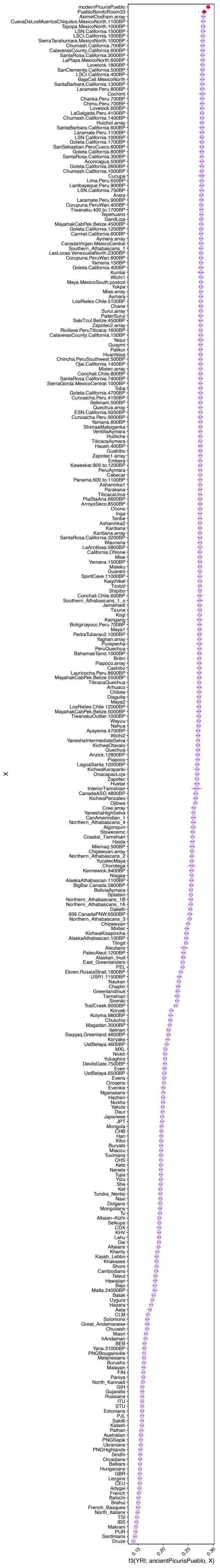
modernPicurisPueblo
PuebloBonitoRoom33
AkimelOodham.array
CuevaDeLosMuertosChiquitos.MexicoNorth.1100BP
Tayopa.MexicoNorth.1000BP
LSN.California.1500BP
LSCI.California.1000BP
SierraTarahumara.MexicoNorth.1000BP
Chumash.California.700BP
CalaverasCounty.California.600BP
SantaRosa.California.3000BP
LaPlaya.MexicoNorth.600BP
Lovelock.1800BP
SanClemente.California.500BP
LSCI.California.400BP
BajaCali.MexicoNorth
SantaBarbara.California.1300BP
Laramate.Peru.800BP
Cochimi
Chanka.Peru.700BP
Chimu.Peru.700BP
Lovelock.600BP
LaGalgada.Peru.4100BP
Chumash.California.1400BP
Huichol.array
SantaBarbara.California.600BP
Laramate.Peru.1100BP
LSN.California.1000BP
Goleta.California.1700BP
SanSebastian.PeruCusco.600BP
Goleta.California.800BP
SantaRosa.California.300BP
Aconcagua.500BP
Goleta.California.2600BP
Chumash.California.1000BP
Cucupa
Lima.Peru.650BP
Lambayeque.Peru.900BP
LSN.California.750BP
Arara
Laramate.Peru.900BP
Corupuna.PeruWari.400BP
Tiwanaku.400.to.1700BP
Tepehuano
GanilLoja
MayahakCabPek.Belize.4500BP
Goleta.California.1200BP
Carmel.California.600BP
Aymara.array
CanadaVirgen.MexicoCentral
Southern_Athabascans_1
LasLocas.VenezuelaNorth.2300BP
Corupuna.PeruWari.600BP
Yamana.150BP
Goleta.California.400BP
Kumiai
Wichi1
Maya.MexicoSouth.postcol
Yukpa
Mixe.array
Aymara
LosRieles.Chile.5100BP
Chane
Surui.array
PaiterSurui
SakiTzul.Belize.4500BP
Zapotec2.array
Riollave.PeruTiticaca.1600BP
CalaverasCounty.California.150BP
Yaqui
Guaymi
Palikur
Huambisa
Chincha.PeruSouthwest.500BP
Ojai.California.1400BP
Mixtec.array
Conchali.Chile.800BP
SantaRosa.California.7400BP
SierraGorda.MexicoCentral.1000BP
Toba
Goleta.California.4700BP
Cuncaicha.Peru.4100BP
Selknam.500BP
Quechua.array
ESN.California.5000BP
Cuncaicha.Peru.9000BP
Yamana.800BP
ShimaaMatsigenka
VentillaAymara
Huilliche
TiticacaAymara
Haush.400BP
Guahibo
Zapotec1.array
Embera
Kaweskar.800.to.1200BP
PeruAymara
Cabecar
Panama.600.to.1100BP
Ashaninka1
Parakana
TiticacaUros
PtaStaAna.6600BP
ArroyoSeco.8500BP
Chono
Inga
Teribe
Ashaninka2
Karitiana
Karitiana.array
SantaRosa.California.3200BP
Waunana
LaArcillosa.5800BP
California.Ohlone
Mixe
Yamana.1500BP
Maleku
Guarani
SpiritCave.11000BP
Kaqchikel
Tzotzil
Shipibo
Conchali.Chile.600BP
Southern_Athabascans_1_o
Jamamadi
Ticuna
Kogi
Kaingang
Botigiriayocc.Peru.700BP
Maya1
PedraTubarao2.1000BP
Yaghan.array
Purepecha
PeruQuechua
BahamasTaino.1000BP
Bribri
Piapoco.array
Cashibo
Lauricocha.Peru.8600BP
MayahakCabPek.Belize.5500BP
TiticacaQuechua
Arhuaco
Chilote
Diaguita
Maya2
LosRieles.Chile.12000BP
MayahakCabPek.Belize.5000BP
TiwanakuOutlier.1000BP
Wayuu
Nahua
Ayayema.4700BP
Wichi2
YaneshaIntermediateSelva
KichwaOtavalo
Quechua
Anzick.12800BP
Piapoco
LagoaSanta.10000BP
KichwaKarapanki
OnacapacLoja
Zapotec
Huetar
InteriorTsimshian
CanadaASO.4800BP
KichwaPanzaleo
Ojibwa
Cree.array
YaneshaHighSelva
CanAmerindian_1
Northern_Athabascans_4
Algonquin
Stswecemc
Coastal_Tsimshian
Haida
Mikmaq.500BP
Chipewyan.array
Northern_Athabascans_2
YucatecMaya
Chorotega
Kennewick.9400BP
Nisgaa
AlaskaAthabascan.1100BP
BigBar.Canada.5600BP
BoliviaAymara
Splatsin
Northern_Athabascans_1B
Northern_Athabascans_1A
Dakelh
939.CanadaPNW.6500BP
Northern_Athabascans_3
Chipewyan
Mixtec
KichwaKisapincha
AlaskaAthabascan.100BP
Tlingit
Aleutians
PaleoAleut.1200BP
Alaskan_Inuit
East_Greenlanders
PEL
Ekven.RussiaStrait.1800BP
USR1.11500BP
Naukan
Chaplin
GreenlandInuit
Tsimshian
Sireniki
TrailCreek.9000BP
Koryak
Kolyma.9800BP
Chukchis
Magadan.3000BP
Itelmen
Saqqaq.Greenland.4600BP
Koryaks
UstBelaya.4600BP
MXL
Nivkh
Yukaghirs
DevilsGate.7500BP
Even
UstBelaya.6500BP
Evens
Oroqens
Evenks
Nganasans
Hezhen
Nivkhs
Yakuts
Daur
Japanese
JPT
Mongola
CHB
Han
Xibo
Buryats
Miaozu
Tuvinians
CHS
Kets
Nenets
Tujia
Yizu
She
Ket
Tundra_Nentsi
Naxi
Dolgans
Mongolians
Tu
Altaian-Kizhi
Selkups
CDX
KHV
Lahu
Dai
Altaians
Khanty
Kayah_Lebbo
Khakases
Shors
Cambodians
Teleut
Hawaiian
Bajo
Malta.24000BP
Batak
Uygurs
Hazara
Aeta
CLM
Solomons
Great_Andamanese
Chuvash
Maori
hAndaman
BEB
Yana.31000BP
PNGBougainville
Melanesians
Burusho
Malayan
FIN
Paniya
North_Kannadi
GIH
Gujaratis
Russians
ITU
STU
Estonians
PJL
Saklit
Kalash
Pathan
Australian
PNGSepik
Ukranians
PNGHighlands
Sindhi
Orcadians
Balkars
Hungarians
GBR
Lezgins
CEU
Adygei
French
Balochi
Brahui
French_Basques
North_Italians
TSI
IBS
Makrani
PUR
Sardinians
Druze
X
0.15
0.20
0.25
0.30
f3(YRI; ancientPicurisPueblo, X)

Supplemental Figure S15 Outgroup *f*3-statistics in the format *f*3(ancientPicurisPueblo, pop, Yorubas) performed on the Illumina-chip array positions.

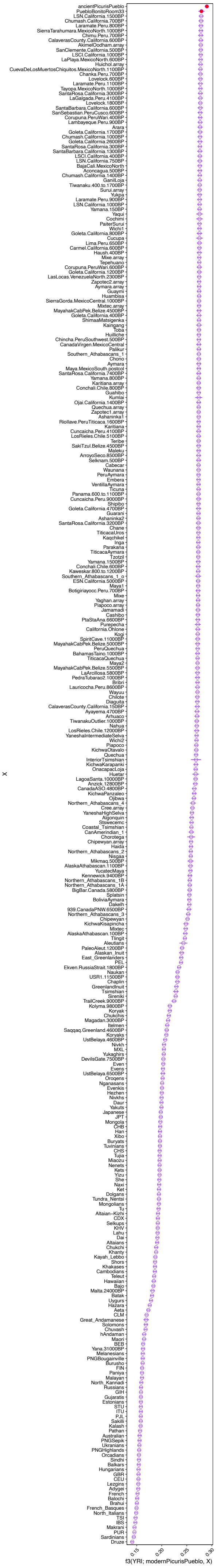

ancientPicurisPueblo
PuebloBonitoRoom33
LSN.California.1500BP
Chumash.California.700BP
Laramate.Peru.800BP
SierraTarahumara.MexicoNorth.1000BP
Chimu.Peru.700BP
CalaverasCounty.California.600BP
AkimelOodham.array
SanClemente.California.500BP
LSCI.California.1000BP
LaPlaya.MexicoNorth.600BP
Huichol.array
CuevaDeLosMuertosChiquitos.MexicoNorth.1100BP
Chanka.Peru.700BP
Lovelock.600BP
Laramate.Peru.1100BP
Tayopa.MexicoNorth.1000BP
SantaRosa.California.3000BP
LaGalgada.Peru.4100BP
Lovelock.1800BP
SantaBarbara.California.600BP
SanSebastian.PeruCusco.600BP
Corupuna.PeruWari.400BP
Lambayeque.Peru.900BP
Arara
Goleta.California.1700BP
Chumash.California.1000BP
Goleta.California.2600BP
SantaRosa.California.300BP
SantaBarbara.California.1300BP
LSCI.California.400BP
LSN.California.750BP
BajaCali.MexicoNorth
Aconcagua.500BP
Chumash.California.1400BP
GanilLoja
Tiwanaku.400.to.1700BP
Surui.array
Yukpa
Laramate.Peru.900BP
LSN.California.1000BP
Yamana.150BP
Yaqui
Cochimi
PaiterSurui
Wichi1
Goleta.California.800BP
Cucupa
Lima.Peru.650BP
Carmel.California.600BP
Haush.400BP
Mixe.array
Tepehuano
Corupuna.PeruWari.600BP
Goleta.California.1200BP
LasLocas.VenezuelaNorth.2300BP
Zapotec2.array
Aymara.array
Guaymi
Huambisa
SierraGorda.MexicoCentral.1000BP
Mixtec.array
MayahakCabPek.Belize.4500BP
Goleta.California.400BP
ShimaaMatsigenka
Kaingang
Toba
Huilliche
Chincha.PeruSouthwest.500BP
CanadaVirgen.MexicoCentral
Palikur
Southern_Athabascans_1
Chono
Aymara
Maya.MexicoSouth.postcol
SantaRosa.California.7400BP
Yamana.800BP
Karitiana.array
Conchali.Chile.800BP
Guahibo
Kumiai
Ojai.California.1400BP
Quechua.array
Zapotec1.array
Ashaninka1
RioIlave.PeruTiticaca.1600BP
Karitiana
Cuncaicha.Peru.4100BP
LosRieles.Chile.5100BP
Teribe
SakiTzul.Belize.4500BP
Maleku
ArroyoSeco.8500BP
Selknam.500BP
Cabecar
Waunana
PeruAymara
Embera
VentillaAymara
Ticuna
Panama.600.to.1100BP
Cuncaicha.Peru.9000BP
Shipibo
Goleta.California.4700BP
Guarani
Ashaninka2
SantaRosa.California.3200BP
Chane
TiticacaUros
Kaqchikel
Inga
Parakana
TiticacaAymara
Tzotzil
Yamana.1500BP
Conchali.Chile.600BP
Kaweskar.800.to.1200BP
Southern_Athabascans_1_o
ESN.California.5000BP
Maya1
Botigiriayocc.Peru.700BP
Mixe
Yaghan.array
Piapoco.array
Jamamadi
Cashibo
PtaStaAna.6600BP
Purepecha
California.Ohlone
Kogi
SpiritCave.11000BP
MayahakCabPek.Belize.5000BP
PeruQuechua
BahamasTaino.1000BP
TiticacaQuechua
Maya2
MayahakCabPek.Belize.5500BP
LaArcillosa.5800BP
PedraTubarao2.1000BP
Bribri
Lauricocha.Peru.8600BP
Wayuu
Chilote
Diaguita
CalaverasCounty.California.150BP
Ayayema.4700BP
Arhuaco
TiwanakuOutlier.1000BP
Nahua
LosRieles.Chile.12000BP
YaneshaIntermediateSelva
Wichi2
Piapoco
KichwaOtavalo
Quechua
InteriorTsimshian
KichwaKarapanki
OnacapacLoja
Huetar
LagoaSanta.10000BP
Anzick.12800BP
CanadaSO.4800BP
KichwaPanzaleo
Ojibwa
Northern_Athabascans_4
Cree.array
YaneshaHighSelva
Algonquin
Stswecemc
Coastal_Tsimshian
CanAmerindian_1
Chorotega
Chipewyan.array
Haida
Northern_Athabascans_2
Nisgaa
Mikmaq.500BP
AlaskaAthabascan.1100BP
YucatecMaya
Kennewick.9400BP
Northern_Athabascans_1B
Northern_Athabascans_1A
BigBar.Canada.5800BP
Splatsin
BoliviaAymara
Dakelh
939.CanadaPNW.6500BP
Northern_Athabascans_3
Chipewyan
KichwaKisapincha
Mixtec
AlaskaAthabascan.100BP
Tlingit
Aleutians
PaleoAleut.1200BP
Alaskan_Inuit
East_Greenlanders
PEL
Ekven.RussiaStrait.1800BP
Naukan
USR1.11500BP
Chaplin
GreenlandInuit
Tsimshian
Sireniki
TrailCreek.9000BP
Kolyma.9800BP
Koryak
Chukchis
Magadan.3000BP
Itelmen
Saqqaq.Greenland.4600BP
Koryaks
UstBelaya.4600BP
Nivkh
MXL
Yukaghirs
DevilsGate.7500BP
Even
Evens
UstBelaya.6500BP
Oroqens
Nganasans
Evenkis
Hezhen
Nivkhs
Daur
Yakuts
Japanese
JPT
Mongola
CHB
Han
Xibo
Buryats
Tuvinians
CHS
Tujia
Miaozu
Nenets
Kets
Yizu
She
Naxi
Ket
Dolgans
Tundra_Nentsi
Mongolians
Tu
Altaian–Kizhi
CDX
Selkups
KHV
Lahu
Dai
Altaians
Chukchi
Khanty
Kayah_Lebbo
Shors
Khakases
Cambodians
Teleut
Hawaiian
Bajo
Malta.24000BP
Batak
Uygurs
Hazara
Aeta
CLM
Great_Andamanese
Solomons
Chuvash
hAndaman
Maori
BEB
Yana.31000BP
Melanesians
PNGBougainville
Burusho
FIN
Paniya
Malayan
North_Kannadi
Russians
GIH
Gujaratis
Estonians
STU
ITU
PJL
Saklli
Kalash
Pathan
Australian
PNGSepik
Ukranians
PNGHighlands
Orcadians
Sindhi
Balkars
Hungarians
GBR
CEU
Lezgins
Adygei
French
Balochi
Brahui
French_Basques
North_Italians
TSI
IBS
Makrani
PUR
Sardinians
Druze
X
0.15
0.20
0.25
0.30
f3(YRI; modernPicurisPueblo, X)

Supplemental Figure S16 Outgroup *f*3-statistics in the format *f*3(modernPicurisPueblo, pop, Yorubas) performed on the Illumina-chip array positions.

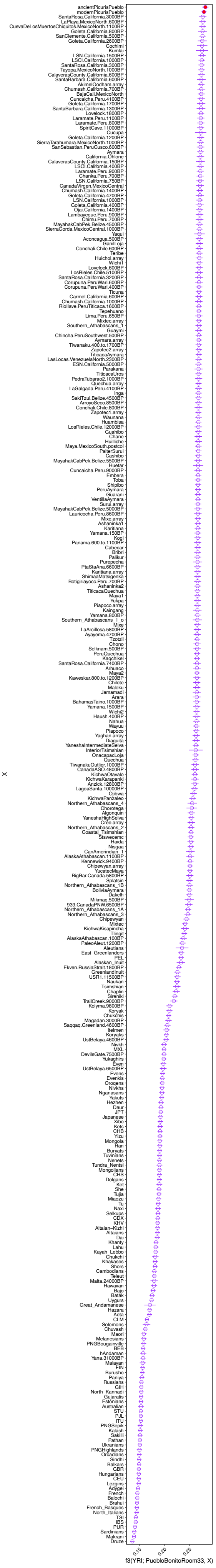
X
0.15
0.20
0.25
f3(YRI; PuebloBonitoRoom33, X)

Supplemental Figure S17 Outgroup *f*3-statistics in the format *f*3(PuebloBonitoRoom33, pop, Yorubas) performed on the Illumina-chip array positions.

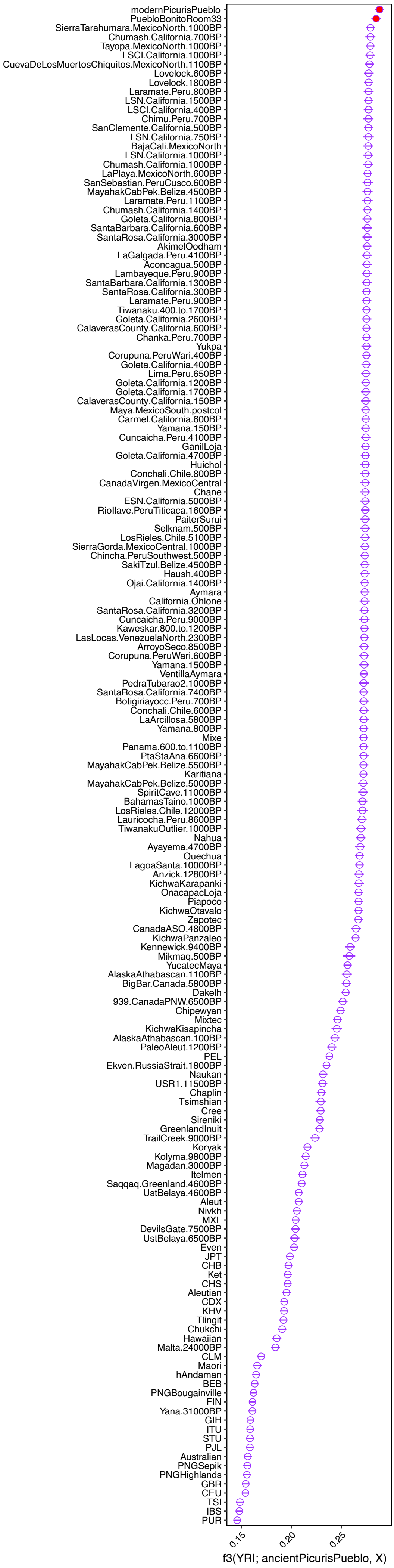
modernPicurisPueblo
PuebloBonitoRoom33
SierraTarahumara.MexicoNorth.1000BP
Chumash.California.700BP
Tayopa.MexicoNorth.1000BP
LSCI.California.1000BP
CuevaDeLosMuertosChiquitos.MexicoNorth.1100BP
Lovelock.600BP
Lovelock.1800BP
Laramate.Peru.800BP
LSN.California.1500BP
LSCI.California.400BP
Chimu.Peru.700BP
SanClemente.California.500BP
LSN.California.750BP
BajaCali.MexicoNorth
LSN.California.1000BP
Chumash.California.1000BP
LaPlaya.MexicoNorth.600BP
SanSebastian.PeruCusco.600BP
MayahakCabPek.Belize.4500BP
Laramate.Peru.1100BP
Chumash.California.1400BP
Goleta.California.800BP
SantaBarbara.California.600BP
SantaRosa.California.3000BP
AkimelOodham
LaGalgada.Peru.4100BP
Aconcagua.500BP
Lambayeque.Peru.900BP
SantaBarbara.California.1300BP
SantaRosa.California.300BP
Laramate.Peru.900BP
Tiwanaku.400.to.1700BP
Goleta.California.2600BP
CalaverasCounty.California.600BP
Chanka.Peru.700BP
Yukpa
Corupuna.PeruWari.400BP
Goleta.California.400BP
Lima.Peru.650BP
Goleta.California.1200BP
Goleta.California.1700BP
CalaverasCounty.California.150BP
Maya.MexicoSouth.postcol
Carmel.California.600BP
Yamana.150BP
Cuncaicha.Peru.4100BP
GanilLoja
Goleta.California.4700BP
Huichol
Conchali.Chile.800BP
CanadaVirgen.MexicoCentral
Chane
ESN.California.5000BP
Riollave.PeruTiticaca.1600BP
PaiterSurui
Selknam.500BP
LosRieles.Chile.5100BP
SierraGorda.MexicoCentral.1000BP
Chincha.PeruSouthwest.500BP
SakiTzul.Belize.4500BP
Haush.400BP
Ojai.California.1400BP
Aymara
California.Ohlone
SantaRosa.California.3200BP
Cuncaicha.Peru.9000BP
Kaweskar.800.to.1200BP
LasLocas.VenezuelaNorth.2300BP
ArroyoSeco.8500BP
Corupuna.PeruWari.600BP
Yamana.1500BP
VentillaAymara
PedraTubarao2.1000BP
SantaRosa.California.7400BP
Botigiriayocc.Peru.700BP
Conchali.Chile.600BP
LaArcillosa.5800BP
Yamana.800BP
Mixe
Panama.600.to.1100BP
PtaStaAna.6600BP
MayahakCabPek.Belize.5500BP
Karitiana
MayahakCabPek.Belize.5000BP
SpiritCave.11000BP
BahamasTaino.1000BP
LosRieles.Chile.12000BP
Lauricocha.Peru.8600BP
TiwanakuOutlier.1000BP
Nahua
Ayayema.4700BP
Quechua
LagoaSanta.10000BP
Anzick.12800BP
KichwaKarapanki
OnacapacLoja
Piapoco
KichwaOtavalo
Zapotec
CanadaASO.4800BP
KichwaPanzaleo
Kennewick.9400BP
Mikmaq.500BP
YucatecMaya
AlaskaAthabascan.1100BP
BigBar.Canada.5800BP
Dakelh
939.CanadaPNW.6500BP
Chipewyan
Mixtec
KichwaKisapincha
AlaskaAthabascan.100BP
PaleoAleut.1200BP
PEL
Ekven.RussiaStrait.1800BP
Naukan
USR1.11500BP
Chaplin
Tsimshian
Cree
Sireniki
GreenlandInuit
TrailCreek.9000BP
Koryak
Kolyma.9800BP
Magadan.3000BP
Itelmen
Saqqaq.Greenland.4600BP
UstBelaya.4600BP
Aleut
Nivkh
MXL
DevilsGate.7500BP
UstBelaya.6500BP
Even
JPT
CHB
Ket
CHS
Aleutian
CDX
KHV
Tlingit
Chukchi
Hawaiian
Malta.24000BP
CLM
Maori
hAndaman
BEB
PNGBougainville
FIN
Yana.31000BP
GIH
ITU
STU
PJL
Australian
PNGSepik
PNGHighlands
GBR
CEU
TSI
IBS
PUR
X
0.15
0.20
0.25
f3(YRI; ancientPicurisPueblo, X)

Supplemental Figure S18 Outgroup $f3$-statistics in the format $f3$(ancientPicurisPueblo, pop, Yorubas) performed on the 1240K hybridization capture positions.

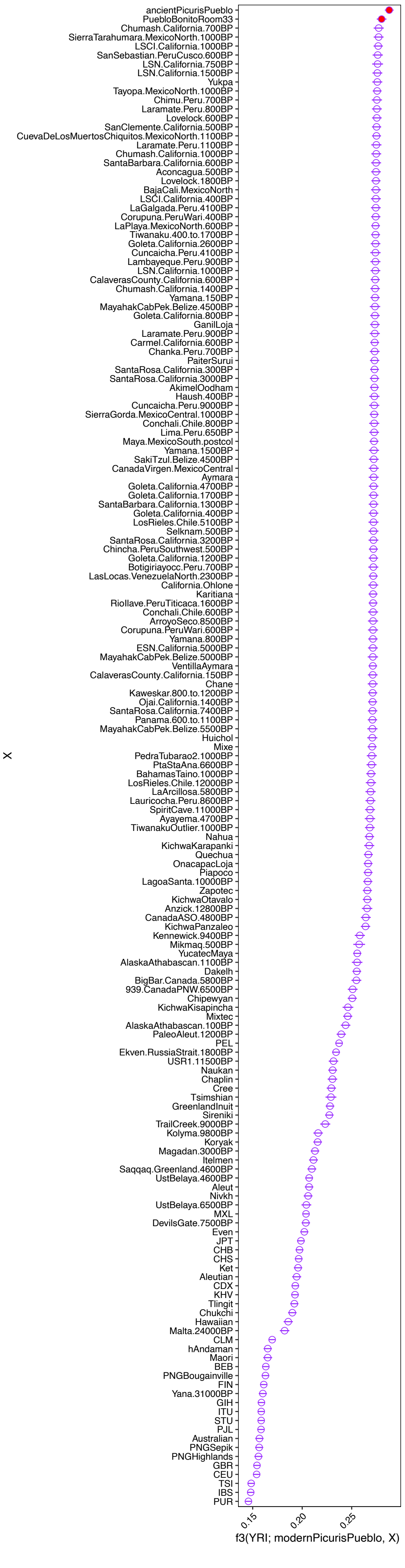

ancientPicurisPueblo
PuebloBonitoRoom33
Chumash.California.700BP
SierraTarahumara.MexicoNorth.1000BP
LSCI.California.1000BP
SanSebastian.PeruCusco.600BP
LSN.California.750BP
LSN.California.1500BP
Yukpa
Tayopa.MexicoNorth.1000BP
Chimu.Peru.700BP
Laramate.Peru.800BP
Lovelock.600BP
SanClemente.California.500BP
CuevaDeLosMuertosChiquitos.MexicoNorth.1100BP
Laramate.Peru.1100BP
Chumash.California.1000BP
SantaBarbara.California.600BP
Aconcagua.500BP
Lovelock.1800BP
BajaCali.MexicoNorth
LSCI.California.400BP
LaGalgada.Peru.4100BP
Corupuna.PeruWari.400BP
LaPlaya.MexicoNorth.600BP
Tiwanaku.400.to.1700BP
Goleta.California.2600BP
Cuncaicha.Peru.4100BP
Lambayeque.Peru.900BP
LSN.California.1000BP
CalaverasCounty.California.600BP
Chumash.California.1400BP
Yamana.150BP
MayahakCabPek.Belize.4500BP
Goleta.California.800BP
GanilLoja
Laramate.Peru.900BP
Carmel.California.600BP
Chanka.Peru.700BP
PaiterSurui
SantaRosa.California.300BP
SantaRosa.California.3000BP
AkimelOodham
Haush.400BP
Cuncaicha.Peru.9000BP
SierraGorda.MexicoCentral.1000BP
Conchali.Chile.800BP
Lima.Peru.650BP
Maya.MexicoSouth.postcol
Yamana.1500BP
SakiTzul.Belize.4500BP
CanadaVirgen.MexicoCentral
Aymara
Goleta.California.4700BP
Goleta.California.1700BP
SantaBarbara.California.1300BP
Goleta.California.400BP
LosRieles.Chile.5100BP
Selknam.500BP
SantaRosa.California.3200BP
Chincha.PeruSouthwest.500BP
Goleta.California.1200BP
Botigiriayocc.Peru.700BP
LasLocas.VenezuelaNorth.2300BP
California.Ohlone
Karitiana
RioIlave.PeruTiticaca.1600BP
Conchali.Chile.600BP
ArroyoSeco.8500BP
Corupuna.PeruWari.600BP
Yamana.800BP
ESN.California.5000BP
MayahakCabPek.Belize.5000BP
VentillaAymara
CalaverasCounty.California.150BP
Chane
Kaweskar.800.to.1200BP
Ojai.California.1400BP
SantaRosa.California.7400BP
Panama.600.to.1100BP
MayahakCabPek.Belize.5500BP
Huichol
Mixe
PedraTubarao2.1000BP
PtaStaAna.6600BP
BahamasTaino.1000BP
LosRieles.Chile.12000BP
LaArcillosa.5800BP
Lauricocha.Peru.8600BP
SpiritCave.11000BP
Ayayema.4700BP
TiwanakuOutlier.1000BP
Nahua
KichwaKarapanki
Quechua
OnacapacLoja
Piapoco
LagoaSanta.10000BP
Zapotec
KichwaOtavalo
Anzick.12800BP
CanadaASO.4800BP
KichwaPanzaleo
Kennewick.9400BP
Mikmaq.500BP
YucatecMaya
AlaskaAthabascan.1100BP
Dakelh
BigBar.Canada.5800BP
939.CanadaPNW.6500BP
Chipewyan
KichwaKisapincha
Mixtec
AlaskaAthabascan.100BP
PaleoAleut.1200BP
PEL
Ekven.RussiaStrait.1800BP
USR1.11500BP
Naukan
Chaplin
Cree
Tsimshian
GreenlandInuit
Sireniki
TrailCreek.9000BP
Kolyma.9800BP
Koryak
Magadan.3000BP
Itelmen
Saqqaq.Greenland.4600BP
UstBelaya.4600BP
Aleut
Nivkh
UstBelaya.6500BP
MXL
DevilsGate.7500BP
Even
JPT
CHB
CHS
Ket
Aleutian
CDX
KHV
Tlingit
Chukchi
Hawaiian
Malta.24000BP
CLM
hAndaman
Maori
BEB
PNGBougainville
FIN
Yana.31000BP
GIH
ITU
STU
PJL
Australian
PNGSepik
PNGHighlands
GBR
CEU
TSI
IBS
PUR
X
0.15
0.20
0.25
f3(YRI; modernPicurisPueblo, X)

Supplemental Figure S19 Outgroup *f*3-statistics in the format *f*3(modernPicurisPueblo, pop, Yorubas) performed on the 1240K hybridization capture positions.

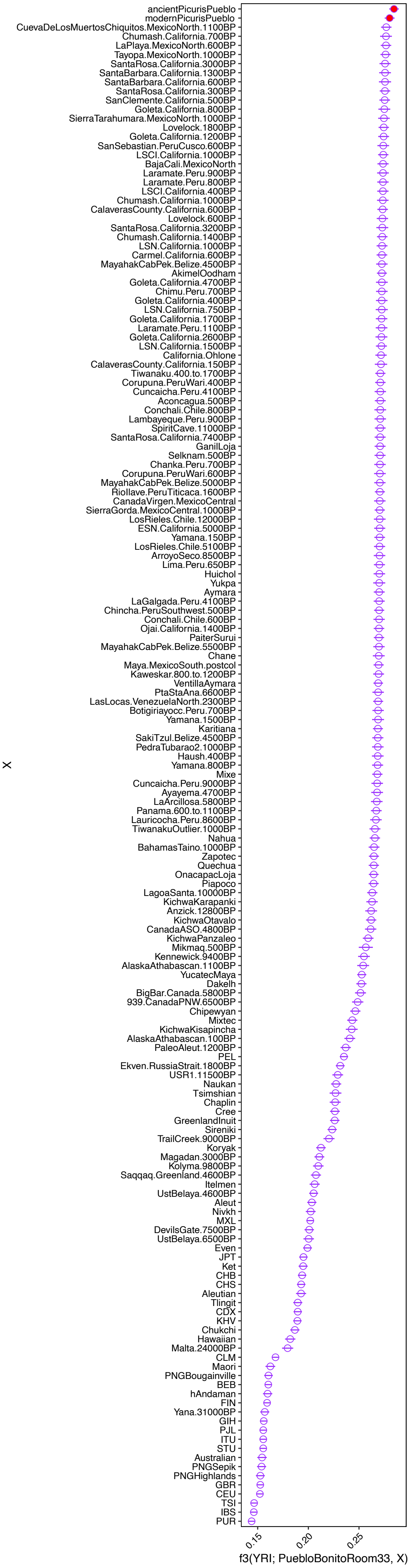
ancientPicurisPueblo
modernPicurisPueblo
CuevaDeLosMuertosChiquitos.MexicoNorth.1100BP
Chumash.California.700BP
LaPlaya.MexicoNorth.600BP
Tayopa.MexicoNorth.1000BP
SantaRosa.California.3000BP
SantaBarbara.California.1300BP
SantaBarbara.California.600BP
SantaRosa.California.300BP
SanClemente.California.500BP
Goleta.California.800BP
SierraTarahumara.MexicoNorth.1000BP
Lovelock.1800BP
Goleta.California.1200BP
SanSebastian.PeruCusco.600BP
LSCI.California.1000BP
BajaCali.MexicoNorth
Laramate.Peru.900BP
Laramate.Peru.800BP
LSCI.California.400BP
Chumash.California.1000BP
CalaverasCounty.California.600BP
Lovelock.600BP
SantaRosa.California.3200BP
Chumash.California.1400BP
LSN.California.1000BP
Carmel.California.600BP
MayahakCabPek.Belize.4500BP
AkimelOodham
Goleta.California.4700BP
Chimu.Peru.700BP
Goleta.California.400BP
LSN.California.750BP
Goleta.California.1700BP
Laramate.Peru.1100BP
Goleta.California.2600BP
LSN.California.1500BP
California.Ohlone
CalaverasCounty.California.150BP
Tiwanaku.400.to.1700BP
Corupuna.PeruWari.400BP
Cuncaicha.Peru.4100BP
Aconcagua.500BP
Conchali.Chile.800BP
Lambayeque.Peru.900BP
SpiritCave.11000BP
SantaRosa.California.7400BP
GanilLoja
Selknam.500BP
Chanka.Peru.700BP
Corupuna.PeruWari.600BP
MayahakCabPek.Belize.5000BP
RioIlave.PeruTiticaca.1600BP
CanadaVirgen.MexicoCentral
SierraGorda.MexicoCentral.1000BP
LosRieles.Chile.12000BP
ESN.California.5000BP
Yamana.150BP
LosRieles.Chile.5100BP
ArroyoSeco.8500BP
Lima.Peru.650BP
Huichol
Yukpa
Aymara
LaGalgada.Peru.4100BP
Chincha.PeruSouthwest.500BP
Conchali.Chile.600BP
Ojai.California.1400BP
PaiterSurui
MayahakCabPek.Belize.5500BP
Chane
Maya.MexicoSouth.postcol
Kaweskar.800.to.1200BP
VentillaAymara
PtaStaAna.6600BP
LasLocas.VenezuelaNorth.2300BP
Botigiriayocc.Peru.700BP
Yamana.1500BP
Karitiana
SakiTzul.Belize.4500BP
PedraTubarao2.1000BP
Haush.400BP
Yamana.800BP
Mixe
Cuncaicha.Peru.9000BP
Ayayema.4700BP
LaArcillosa.5800BP
Panama.600.to.1100BP
Lauricocha.Peru.8600BP
TiwanakuOutlier.1000BP
Nahua
BahamasTaino.1000BP
Zapotec
Quechua
OnacapacLoja
Piapoco
LagoaSanta.10000BP
KichwaKarapanki
Anzick.12800BP
KichwaOtavalo
CanadaASO.4800BP
KichwaPanzaleo
Mikmaq.500BP
Kennewick.9400BP
AlaskaAthabascan.1100BP
YucatecMaya
Dakelh
BigBar.Canada.5800BP
939.CanadaPNW.6500BP
Chipewyan
Mixtec
KichwaKisapincha
AlaskaAthabascan.100BP
PaleoAleut.1200BP
PEL
Ekven.RussiaStrait.1800BP
USR1.11500BP
Naukan
Tsimshian
Chaplin
Cree
GreenlandInuit
Sireniki
TrailCreek.9000BP
Koryak
Magadan.3000BP
Kolyma.9800BP
Saqqaq.Greenland.4600BP
Itelmen
UstBelaya.4600BP
Aleut
Nivkh
MXL
DevilsGate.7500BP
UstBelaya.6500BP
Even
JPT
Ket
CHB
CHS
Aleutian
Tlingit
CDX
KHV
Chukchi
Hawaiian
Malta.24000BP
CLM
Maori
PNGBougainville
BEB
hAndaman
FIN
Yana.31000BP
GIH
PJL
ITU
STU
Australian
PNGSepik
PNGHighlands
GBR
CEU
TSI
IBS
PUR
X
0.15
0.20
0.25
f3(YRI; PuebloBonitoRoom33, X)

Supplemental Figure S20 Outgroup *f*3-statistics in the format *f*3(PuebloBonitoRoom33, pop, Yorubas) performed on the 1240K hybridization capture positions.

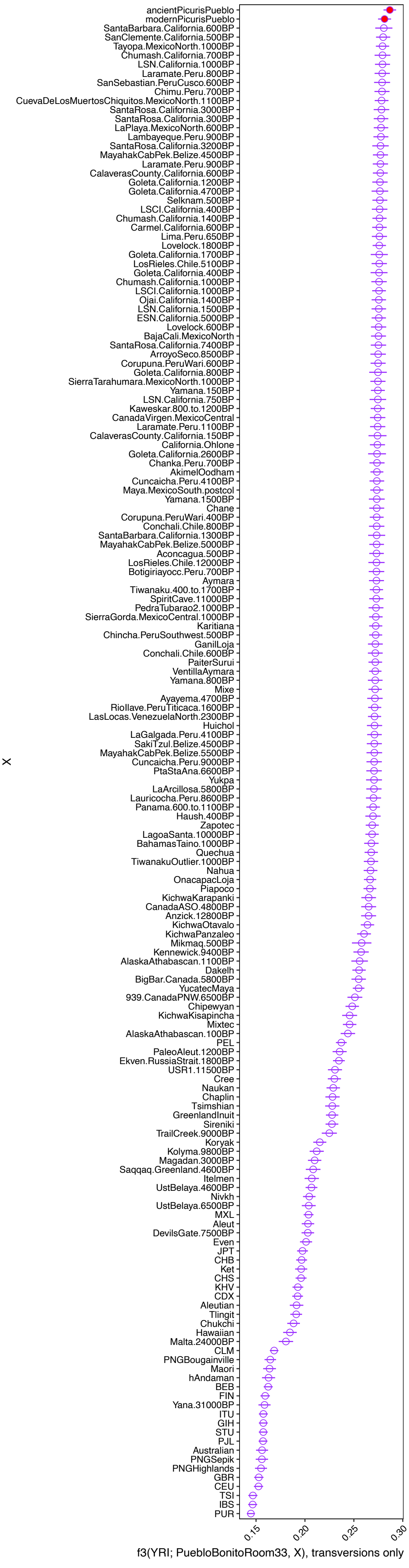
ancientPicurisPueblo
modernPicurisPueblo
SantaBarbara.California.600BP
SanClemente.California.500BP
Tayopa.MexicoNorth.1000BP
Chumash.California.700BP
LSN.California.1000BP
Laramate.Peru.800BP
SanSebastian.PeruCusco.600BP
Chimu.Peru.700BP
CuevaDeLosMuertosChiquitos.MexicoNorth.1100BP
SantaRosa.California.3000BP
SantaRosa.California.300BP
LaPlaya.MexicoNorth.600BP
Lambayeque.Peru.900BP
SantaRosa.California.3200BP
MayahakCabPek.Belize.4500BP
Laramate.Peru.900BP
CalaverasCounty.California.600BP
Goleta.California.1200BP
Goleta.California.4700BP
Selknam.500BP
LSCI.California.400BP
Chumash.California.1400BP
Carmel.California.600BP
Lima.Peru.650BP
Lovelock.1800BP
Goleta.California.1700BP
LosRieles.Chile.5100BP
Goleta.California.400BP
Chumash.California.1000BP
LSCI.California.1000BP
Ojai.California.1400BP
LSN.California.1500BP
ESN.California.5000BP
Lovelock.600BP
BajaCali.MexicoNorth
SantaRosa.California.7400BP
ArroyoSeco.8500BP
Corupuna.PeruWari.600BP
Goleta.California.800BP
SierraTarahumara.MexicoNorth.1000BP
Yamana.150BP
LSN.California.750BP
Kaweskar.800.to.1200BP
CanadaVirgen.MexicoCentral
Laramate.Peru.1100BP
CalaverasCounty.California.150BP
California.Ohlone
Goleta.California.2600BP
Chanka.Peru.700BP
AkimelOodham
Cuncaicha.Peru.4100BP
Maya.MexicoSouth.postcol
Yamana.1500BP
Chane
Corupuna.PeruWari.400BP
Conchali.Chile.800BP
SantaBarbara.California.1300BP
MayahakCabPek.Belize.5000BP
Aconcagua.500BP
LosRieles.Chile.12000BP
Botigiriayocc.Peru.700BP
Aymara
Tiwanaku.400.to.1700BP
SpiritCave.11000BP
PedraTubarao2.1000BP
SierraGorda.MexicoCentral.1000BP
Karitiana
Chincha.PeruSouthwest.500BP
GanilLoja
Conchali.Chile.600BP
PaiterSurui
VentillaAymara
Yamana.800BP
Mixe
Ayayema.4700BP
Riollave.PeruTiticaca.1600BP
LasLocas.VenezuelaNorth.2300BP
Huichol
LaGalgada.Peru.4100BP
SakiTzul.Belize.4500BP
MayahakCabPek.Belize.5500BP
Cuncaicha.Peru.9000BP
PtaStaAna.6600BP
Yukpa
LaArcillosa.5800BP
Lauricocha.Peru.8600BP
Panama.600.to.1100BP
Haush.400BP
Zapotec
LagoaSanta.10000BP
BahamasTaino.1000BP
Quechua
TiwanakuOutlier.1000BP
Nahua
OnacapacLoja
Piapoco
KichwaKarapanki
CanadaASO.4800BP
Anzick.12800BP
KichwaOtavalo
KichwaPanzaleo
Mikmaq.500BP
Kennewick.9400BP
AlaskaAthabascan.1100BP
Dakelh
BigBar.Canada.5800BP
YucatecMaya
939.CanadaPNW.6500BP
Chipewyan
KichwaKisapincha
Mixtec
AlaskaAthabascan.100BP
PEL
PaleoAleut.1200BP
Ekven.RussiaStrait.1800BP
USR1.11500BP
Cree
Naukan
Chaplin
Tsimshian
GreenlandInuit
Sireniki
TrailCreek.9000BP
Koryak
Kolyma.9800BP
Magadan.3000BP
Saqqaq.Greenland.4600BP
Itelmen
UstBelaya.4600BP
Nivkh
UstBelaya.6500BP
MXL
Aleut
DevilsGate.7500BP
Even
JPT
CHB
Ket
CHS
KHV
CDX
Aleutian
Tlingit
Chukchi
Hawaiian
Malta.24000BP
CLM
PNGBougainville
Maori
hAndaman
BEB
FIN
Yana.31000BP
ITU
GIH
STU
PJL
Australian
PNGSepik
PNGHighlands
GBR
CEU
TSI
IBS
PUR
X
0.15
0.20
0.25
0.30
f3(YRI; PuebloBonitoRoom33, X), transversions only

Supplemental Figure S21 Outgroup *f*3-statistics in the format *f*3(PuebloBonitoRoom33, pop, Yorubas) performed on the 1240K hybridization capture transversions.

11.2. *D*-statistics and qpAdm

11.2.1 Population continuity in the North American Southwest

In order to test population continuity in Picuris Pueblo and whether they form a clade with Pueblo Bonito to the exclusion of every other Native American population, we used *D*-statistics.

First, we investigated the relationship between ancient Picuris Pueblo and Pueblo Bonito, by performing the test *D(ancientPicurisPueblo, PuebloBonitoRoom33, pop, Yorubas)*, restricting to transversion polymorphisms, and found no population to significantly deviate from 0, except present-day Picuris Pueblo (D = 0.0288, Z = 4.79), that is significantly closer to ancient Picuris than Pueblo Bonito (**Supplemental Figure S22**).

When we repeat this test by replacing ancient Picuris with present-day Picuris Pueblo (*D(ancientPicurisPueblo, modernPicurisPueblo, pop, Yorubas)*), we recover significant positive (highest value from *PuebloBonitoRoom33*, D = 0.0102, Z= 4.05) and negative values of D (highest in *Kolyma.9800BP*, D= -0.075, Z= -2.88) indicating the presence of additional Indigenous American ancestry in present-day Picuris Pueblo members that is not present in ancient Picuris (**Supplemental Figure S23**). This signal can also be seen on the PCA, with some individuals' positions shifted towards NNA populations (**Supplemental Figure S12**).

To test if the extra Northern Athabascan admixture is enough to explain both positive and negative results, we used admixture-subtracted *D*-statistics[164] and found that when accounting for 10% Northern Athabascan ancestry in present-day Picuris Pueblo using Chipewyan as a source – in the format *f*4(*ancientPicurisPueblo, modernPicurisPueblo, pop, Yorubas*; x=*Chipewyan*) – the test is not significantly different from zero for all other sampled populations (**Supplemental Figure S24**).

This result is similar to the one obtained with Southern Athabascans (see below), and likely reflect admixture with these neighbouring Apachean groups, that had not taken place at the time of sampling of ancient individuals from Picuris Pueblo.

We applied another allele-frequency based method (Treemix[172]) using all sites where the Pueblo Bonito individuals intersected with the imputed dataset, to build a maximum-likelihood tree of North American individuals (**Extended Data Figure 01**). We found Pueblo Bonito and present-day and ancient Picuris to form a clade of their own, in an intermediate position between SNA and NNA populations.

Finally, we conclude that no other sampled population is closer to Ancestral Puebloan individuals from Pueblo Bonito Room 33 than ancient and present-day individuals from Picuris Pueblo. Furthermore, we find a high level of population continuity in Picuris Pueblo, with most of the ancestry of present-day individuals stemming directly from previous occupants of the Pueblo, despite admixture with both European and other local Indigenous groups.

D(ancientPicurisPueblo, PuebloBonitoRoom33; h3, YRI)
transversions only
(PuebloBonitoRoom33,h3) <---> (ancientPicurisPueblo,h3)

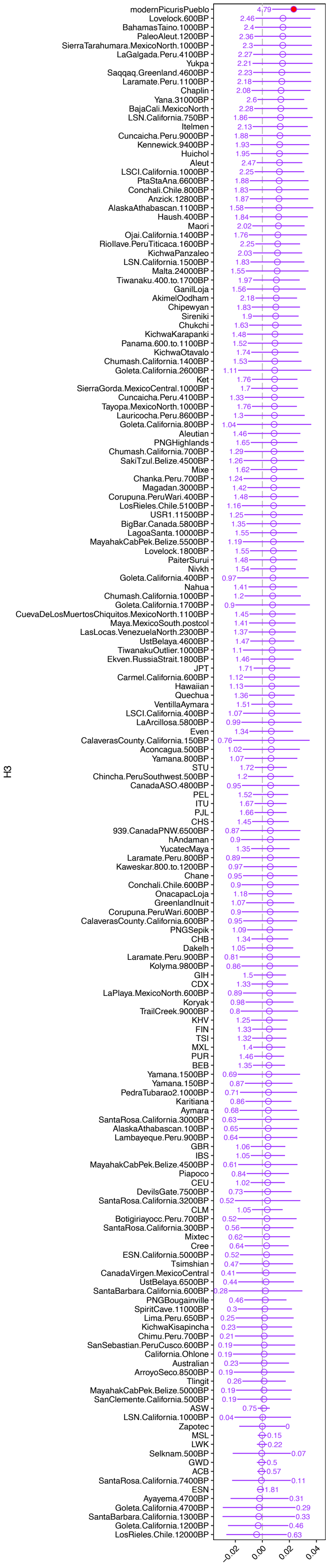

Supplemental Figure S22 D-statistics in the format D(ancientPicurisPueblo, PuebloBonitoRoom33, pop, Yorubas), restricting to transversion polymorphisms in the 1240K hybridization capture set. We find a strong rejection for any other population forming a clade with PicurisPueblo to the exclusion of PuebloBonitoRoom33 and vice-versa, with the sole exception of present-day Picuris Pueblo individuals, which are closer to Ancestral Puebloans from Picuris. Significant statistics are marked with filled red circles.

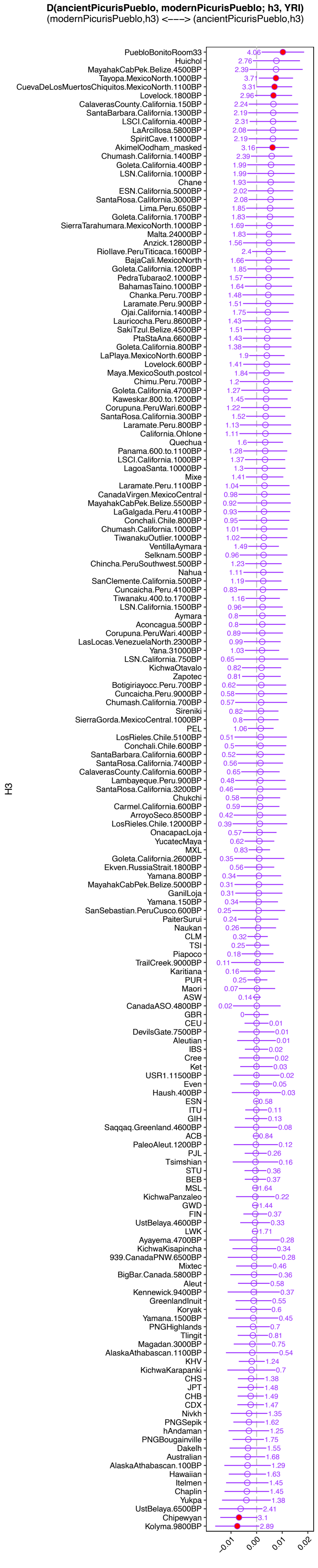
D(ancientPicurisPueblo, modernPicurisPueblo; h3, YRI)
(modernPicurisPueblo,h3) <––> (ancientPicurisPueblo,h3)
H3
PuebloBonitoRoom33 4.06
Huichol 2.76
MayahakCabPek.Belize.4500BP 2.39
Tayopa.MexicoNorth.1000BP 3.71
CuevaDeLosMuertosChiquitos.MexicoNorth.1100BP 3.31
Lovelock.1800BP 2.96
CalaverasCounty.California.150BP 2.24
SantaBarbara.California.1300BP 2.19
LSCI.California.400BP 2.31
LaArcillosa.5800BP 2.08
SpiritCave.11000BP 2.19
AkimelOodham_masked 3.16
Chumash.California.1400BP 2.39
Goleta.California.400BP 1.99
LSN.California.1000BP 1.99
Chane 1.93
ESN.California.5000BP 2.02
SantaRosa.California.3000BP 2.08
Lima.Peru.650BP 1.85
Goleta.California.1700BP 1.83
SierraTarahumara.MexicoNorth.1000BP 1.69
Malta.24000BP 1.83
Anzick.12800BP 1.56
RioIlave.PeruTiticaca.1600BP 2.4
BajaCali.MexicoNorth 1.66
Goleta.California.1200BP 1.85
PedraTubarao2.1000BP 1.57
BahamasTaino.1000BP 1.64
Chanka.Peru.700BP 1.48
Laramate.Peru.900BP 1.51
Ojai.California.1400BP 1.75
Lauricocha.Peru.8600BP 1.43
SakiTzul.Belize.4500BP 1.51
PtaStaAna.6600BP 1.43
Goleta.California.800BP 1.38
LaPlaya.MexicoNorth.600BP 1.9
Lovelock.600BP 1.41
Maya.MexicoSouth.postcol 1.84
Chimu.Peru.700BP 1.2
Goleta.California.4700BP 1.27
Kaweskar.800.to.1200BP 1.45
Corupuna.PeruWari.600BP 1.22
SantaRosa.California.300BP 1.52
Laramate.Peru.800BP 1.13
California.Ohlone 1.11
Quechua 1.6
Panama.600.to.1100BP 1.28
LSCI.California.1000BP 1.37
LagoaSanta.10000BP 1.3
Mixe 1.41
Laramate.Peru.1100BP 1.04
CanadaVirgen.MexicoCentral 0.98
MayahakCabPek.Belize.5500BP 0.92
LaGalgada.Peru.4100BP 0.93
Conchali.Chile.800BP 0.95
Chumash.California.1000BP 1.01
TiwanakuOutlier.1000BP 1.02
VentillaAymara 1.49
Selknam.500BP 0.96
Chincha.PeruSouthwest.500BP 1.23
Nahua 1.11
SanClemente.California.500BP 1.19
Cuncaicha.Peru.4100BP 0.83
Tiwanaku.400.to.1700BP 1.16
LSN.California.1500BP 0.96
Aymara 0.8
Aconcagua.500BP 0.8
Corupuna.PeruWari.400BP 0.89
LasLocas.VenezuelaNorth.2300BP 0.99
Yana.31000BP 1.03
LSN.California.750BP 0.65
KichwaOtavalo 0.82
Zapotec 0.81
Botigiriayocc.Peru.700BP 0.62
Cuncaicha.Peru.9000BP 0.58
Chumash.California.700BP 0.57
Sireniki 0.82
SierraGorda.MexicoCentral.1000BP 0.8
PEL 1.06
LosRieles.Chile.5100BP 0.51
Conchali.Chile.600BP 0.5
SantaBarbara.California.600BP 0.52
SantaRosa.California.7400BP 0.56
CalaverasCounty.California.600BP 0.65
Lambayeque.Peru.900BP 0.48
SantaRosa.California.3200BP 0.46
Chukchi 0.58
Carmel.California.600BP 0.59
ArroyoSeco.8500BP 0.42
LosRieles.Chile.12000BP 0.39
OnacapacLoja 0.57
YucatecMaya 0.62
MXL 0.83
Goleta.California.2600BP 0.35
Ekven.RussiaStrait.1800BP 0.56
Yamana.800BP 0.34
MayahakCabPek.Belize.5000BP 0.31
GanilLoja 0.31
Yamana.150BP 0.34
SanSebastian.PeruCusco.600BP 0.25
PaiterSurui 0.24
Naukan 0.26
CLM 0.32
TSI 0.25
Piapoco 0.18
TrailCreek.9000BP 0.11
Karitiana 0.16
PUR 0.25
Maori 0.07
ASW 0.14
CanadaASO.4800BP 0.02
GBR 0
CEU 0.01
DevilsGate.7500BP 0.01
Aleutian 0.01
IBS 0.02
Cree 0.02
Ket 0.03
USR1.11500BP 0.02
Even 0.05
Haush.400BP 0.03
ESN 0.58
ITU 0.11
GIH 0.13
Saqqaq.Greenland.4600BP 0.08
ACB 0.84
PaleoAleut.1200BP 0.12
PJL 0.26
Tsimshian 0.16
STU 0.36
BEB 0.37
MSL 1.64
KichwaPanzaleo 0.22
GWD 1.44
FIN 0.37
UstBelaya.4600BP 0.33
LWK 1.71
Ayayema.4700BP 0.28
KichwaKisapincha 0.34
939.CanadaPNW.6500BP 0.28
Mixtec 0.46
BigBar.Canada.5800BP 0.36
Aleut 0.58
Kennewick.9400BP 0.37
GreenlandInuit 0.55
Koryak 0.6
Yamana.1500BP 0.45
PNGHighlands 0.7
Tlingit 0.81
Magadan.3000BP 0.75
AlaskaAthabascan.1100BP 0.54
KHV 1.24
KichwaKarapanki 0.7
CHS 1.38
JPT 1.48
CHB 1.49
CDX 1.47
Nivkh 1.35
PNGSepik 1.62
hAndaman 1.25
PNGBougainville 1.75
Dakelh 1.55
Australian 1.68
AlaskaAthabascan.100BP 1.29
Hawaiian 1.63
Itelmen 1.45
Chaplin 1.45
Yukpa 1.38
UstBelaya.6500BP 2.41
Chipewyan 3.1
Kolyma.9800BP 2.89
-0.01
0.00
0.01
0.02

Supplemental Figure S23 D-statistics in the format D(ancientPicurisPueblo, modernPicurisPueblo, pop, Yorubas) in the 1240K hybridization capture set. Significant statistics are marked with filled red circles. We find present-day members of Picuris Pueblo to carry additional Indigenous American ancestry that is not present in Ancestral Puebloans from Picuris Pueblo.

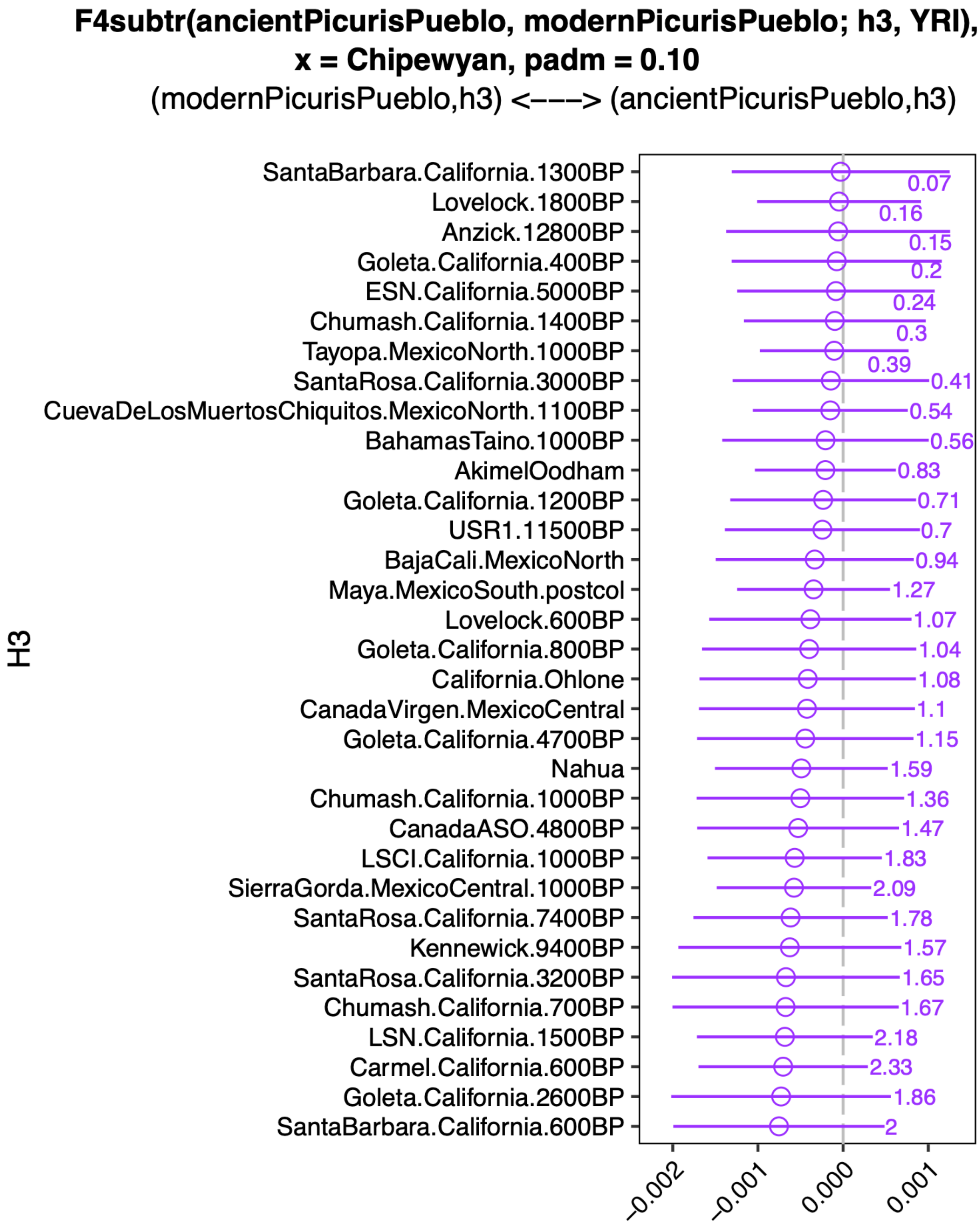

Supplemental Figure S24 Admixture subtracted *F*4-statistics in the format subtrF4(modernPicurisPueblo, ancientPicurisPueblo, pop, Yorubas; x=Chipewyan, padm=0.1) in the 1240K hybridization capture set, accounting 10% admixture from Chipewyan. When this extra Indigenous American is subtracted, we find no population, past or present, to form a clade with present-day Picuris Pueblo to the exclusion of Ancestral Puebloans from the same locality.

11.2.2. Relationship with SNA and NNA groups

In a continent-wide scale, it became a staple to divide Native American ancestry in Southern Native American (SNA) and Northern Native American (NNA) (per [145], see [173]), where SNA groups form a clade with 12.8 kya Anzick-1 genome and NNA do not. This definition intends to represent a very deep split between Native American groups, older than 13 kya, even though its range, in both space and time, it is still very poorly understood. When running a four-population test in the configuration *D(pop, Karitiana, Anzick.12800BP, Yorubas* or *Han*) (as in the original[145] – many other tests are possible and yield the same results) we see that many North American populations have Z-scores below -2.7, showing a statistically significant rejection of the scenario where those groups are equally related to Anzick1 as the Southern Amazonian Karitiana. Those populations include all Athabascan- and Algic-speakers (including Southern Athabascans), as well as all individuals from the Pacific Northwest (following [131]). However, it also includes other individuals such as Kennewick Man ('The Ancient One'), Ancient Southwestern Ontario (ASO) and many present-day and ancient individuals from North Mexico (Akimel O'odham, Yaqui, LaPlaya.600BP, CuevaDeLosMuertosChiquitos.1100BP, Tayopa.1000BP), as well as ancient and present-day Picuris Pueblo and Ancestral Puebloans from Pueblo Bonito (**Supplemental Figure S25**), who are better modelled as a mixture of SNA plus a Native American source upstream of the split with Anzick1[114].

When running a test of the form *D(pop, Karitiana, NNA, USR1.11500BP)*, where h1 is either Akimel O'odham, ancient Picuris Pueblo, LaPlaya.600BP, Tayopa.1000BP or CuevaDeLosMuertosChiquitos.1100BP, we found no population to yield a significantly positive result (maximum values for *D* and Z-score for *D*(*LaPlaya.600BP, Karitiana, CanAmerindian_1, USR1.11500BP*), *D* = 0.011, Z=1.82) (**Supplemental Figure S26-S30**). This is in contrast with Ancient Southwestern Ontario (CanadaASO.4800BP), which consistently show strong positive results when Algonquin, Ojibwa or Cree are in h3 (maximum *D* = 0.025 with Cree, maximum Z=3.94 with Ojibwa), indicating these populations are a good source for the non-SNA ancestry in ASO (**Supplemental Figure S31**).

In order to test if either Picuris or Akimel O'odham carry more of this unsampled ancestry than the other, we run all tests in the configuration *D*(*PicurisPueblo, AkimelOodham, SNA, Yorubas*) and found this test to be consistently negative (**Supplemental Figure S32**), which could indicate that Picuris carried more of this ancestry. However, this result is non-significative if any of the available Early Holocene genomes sampled is in position h3 (LagoaSanta_10000BP, *D* = -0.003, Z = -1.63; LosRieles.12000BP, *D* = -0.004, Z = -1.47; Anzick.12800BP, *D* = -0.003, Z = -1.1; SpiritCave.11000BP, *D* = -0.0001, Z= -0.04; Cuncaicha.9000BP, *D* = -0.001, Z = -0.33; Lauricocha.8600BP, *D* = -0.001, Z = -0.33). Therefore, this result could also be explained by the fact that most present-day sampled SNA can be modelled as carrying some Mesoamerican ancestry (maximised in Mixe)[114], and that Akimel O'odham have significantly more ancestry from Mesoamerica than Picuris (in the test *D*(*ancientPicurisPueblo, AkimelOodham, Mixe, YRI*), *D* = -0.08, Z = -4.26).

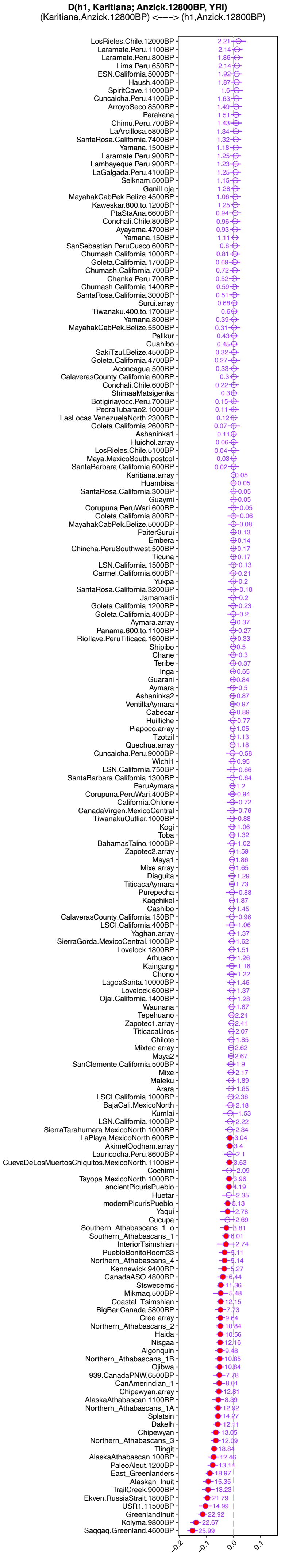
D(h1, Karitiana; Anzick.12800BP, YRI)
(Karitiana,Anzick.12800BP) <---> (h1,Anzick.12800BP)
H1
LosRieles.Chile.12000BP 2.21
Laramate.Peru.1100BP 2.14
Laramate.Peru.800BP 1.86
Lima.Peru.650BP 2.14
ESN.California.5000BP 1.92
Haush.400BP 1.87
SpiritCave.11000BP 1.6
Cuncaicha.Peru.4100BP 1.63
ArroyoSeco.8500BP 1.49
Parakana 1.51
Chimu.Peru.700BP 1.43
LaArcillosa.5800BP 1.34
SantaRosa.California.7400BP 1.32
Yamana.1500BP 1.18
Laramate.Peru.900BP 1.25
Lambayeque.Peru.900BP 1.23
LaGalgada.Peru.4100BP 1.25
Selknam.500BP 1.15
GanilLoja 1.28
MayahakCabPek.Belize.4500BP 1.06
Kaweskar.800.to.1200BP 1.25
PtaStaAna.6600BP 0.94
Conchali.Chile.800BP 0.96
Ayayema.4700BP 0.93
Yamana.150BP 1.11
SanSebastian.PeruCusco.600BP 0.8
Chumash.California.1000BP 0.81
Goleta.California.1700BP 0.69
Chumash.California.700BP 0.72
Chanka.Peru.700BP 0.52
Chumash.California.1400BP 0.59
SantaRosa.California.3000BP 0.51
Surui.array 0.68
Tiwanaku.400.to.1700BP 0.6
Yamana.800BP 0.39
MayahakCabPek.Belize.5500BP 0.31
Palikur 0.43
Guahibo 0.45
SakiTzul.Belize.4500BP 0.32
Goleta.California.4700BP 0.27
Aconcagua.500BP 0.33
CalaverasCounty.California.600BP 0.3
Conchali.Chile.600BP 0.22
ShimaaMatsigenka 0.3
Botigiriayocc.Peru.700BP 0.15
PedraTubarao2.1000BP 0.11
LasLocas.VenezuelaNorth.2300BP 0.12
Goleta.California.2600BP 0.07
Ashaninka1 0.11
Huichol.array 0.06
LosRieles.Chile.5100BP 0.04
Maya.MexicoSouth.postcol 0.03
SantaBarbara.California.600BP 0.02
Karitiana.array -0.05
Huambisa -0.05
SantaRosa.California.300BP -0.05
Guaymi -0.05
Corupuna.PeruWari.600BP -0.05
Goleta.California.800BP -0.06
MayahakCabPek.Belize.5000BP -0.08
PaiterSurui -0.13
Embera -0.14
Chincha.PeruSouthwest.500BP -0.17
Ticuna -0.17
LSN.California.1500BP -0.13
Carmel.California.600BP -0.21
Yukpa -0.2
SantaRosa.California.3200BP -0.18
Jamamadi -0.2
Goleta.California.1200BP -0.23
Goleta.California.400BP -0.2
Aymara.array -0.37
Panama.600.to.1100BP -0.27
Riollave.PeruTiticaca.1600BP -0.33
Shipibo -0.5
Chane -0.3
Teribe -0.37
Inga -0.65
Guarani -0.84
Aymara -0.5
Ashaninka2 -0.87
VentillaAymara -0.97
Cabecar -0.89
Huilliche -0.77
Piapoco.array -1.05
Tzotzil -1.13
Quechua.array -1.18
Cuncaicha.Peru.9000BP -0.58
Wichi1 -0.95
LSN.California.750BP -0.66
SantaBarbara.California.1300BP -0.64
PeruAymara -1.2
Corupuna.PeruWari.400BP -0.94
California.Ohlone -0.72
CanadaVirgen.MexicoCentral -0.76
TiwanakuOutlier.1000BP -0.88
Kogi -1.06
Toba -1.32
BahamasTaino.1000BP -1.02
Zapotec2.array -1.59
Maya1 -1.86
Mixe.array -1.65
Diaguita -1.29
TiticacaAymara -1.73
Purepecha -0.88
Kaqchikel -1.87
Cashibo -1.45
CalaverasCounty.California.150BP -0.96
LSCl.California.400BP -1.06
Yaghan.array -1.37
SierraGorda.MexicoCentral.1000BP -1.62
Lovelock.1800BP -1.51
Arhuaco -1.26
Kaingang -1.16
Chono -1.22
LagoaSanta.10000BP -1.46
Lovelock.600BP -1.37
Ojai.California.1400BP -1.28
Waunana -1.67
Tepehuano -2.24
Zapotec1.array -2.41
TiticacaUros -2.07
Chilote -1.85
Mixtec.array -2.62
Maya2 -2.67
SanClemente.California.500BP -1.9
Mixe -2.17
Maleku -1.89
Arara -1.85
LSCl.California.1000BP -2.38
BajaCali.MexicoNorth -2.18
Kumlai -1.53
LSN.California.1000BP -2.22
SierraTarahumara.MexicoNorth.1000BP -2.34
LaPlaya.MexicoNorth.600BP -3.04
AkimelOodham.array -3.4
Lauricocha.Peru.8600BP -2.1
CuevaDeLosMuertosChiquitos.MexicoNorth.1100BP -3.63
Cochimi -2.09
Tayopa.MexicoNorth.1000BP -3.96
ancientPicurisPueblo -4.19
Huetar -2.35
modernPicurisPueblo -5.13
Yaqui -2.78
Cucupa -2.69
Southern_Athabascans_1_o -3.81
Southern_Athabascans_1 -6.01
InteriorTsimshian -2.74
PuebloBonitoRoom33 -5.11
Northern_Athabascans_4 -5.14
Kennewick.9400BP -5.27
CanadaASO.4800BP -6.44
Stswecemc -11.36
Mikmaq.500BP -5.48
Coastal_Tsimshian -12.15
BigBar.Canada.5800BP -7.73
Cree.array -9.64
Northern_Athabascans_2 -10.84
Haida -10.56
Nisgaa -12.16
Algonquin -9.48
Northern_Athabascans_1B -10.85
Ojibwa -10.84
939.CanadaPNW.6500BP -7.78
CanAmerindian_1 -8.01
Chipewyan.array -12.81
AlaskaAthabascan.1100BP -8.39
Northern_Athabascans_1A -12.92
Splatsin -14.27
Dakelh -12.11
Chipewyan -13.05
Northern_Athabascans_3 -12.09
Tlingit -18.84
AlaskaAthabascan.100BP -12.46
PaleoAleut.1200BP -13.14
East_Greenlanders -18.97
Alaskan_Inuit -15.35
TrailCreek.9000BP -13.23
Ekven.RussiaStrait.1800BP -21.79
USR1.11500BP -14.99
GreenlandInuit -22.92
Kolyma.9800BP -22.67
Saqqaq.Greenland.4600BP -25.99
-0.2
-0.1
0.0
0.1

Supplemental Figure S25 D-statistics in the format D(pop, Karitiana, Anzick.12800, Yorubas) on the Illumina-chip array positions. Many individuals fail to form a clade with Southern Amazonian Karitiana (negative values; red filled circles indicate significant results), indicating the presence of some ancestry upstream of Anzick.

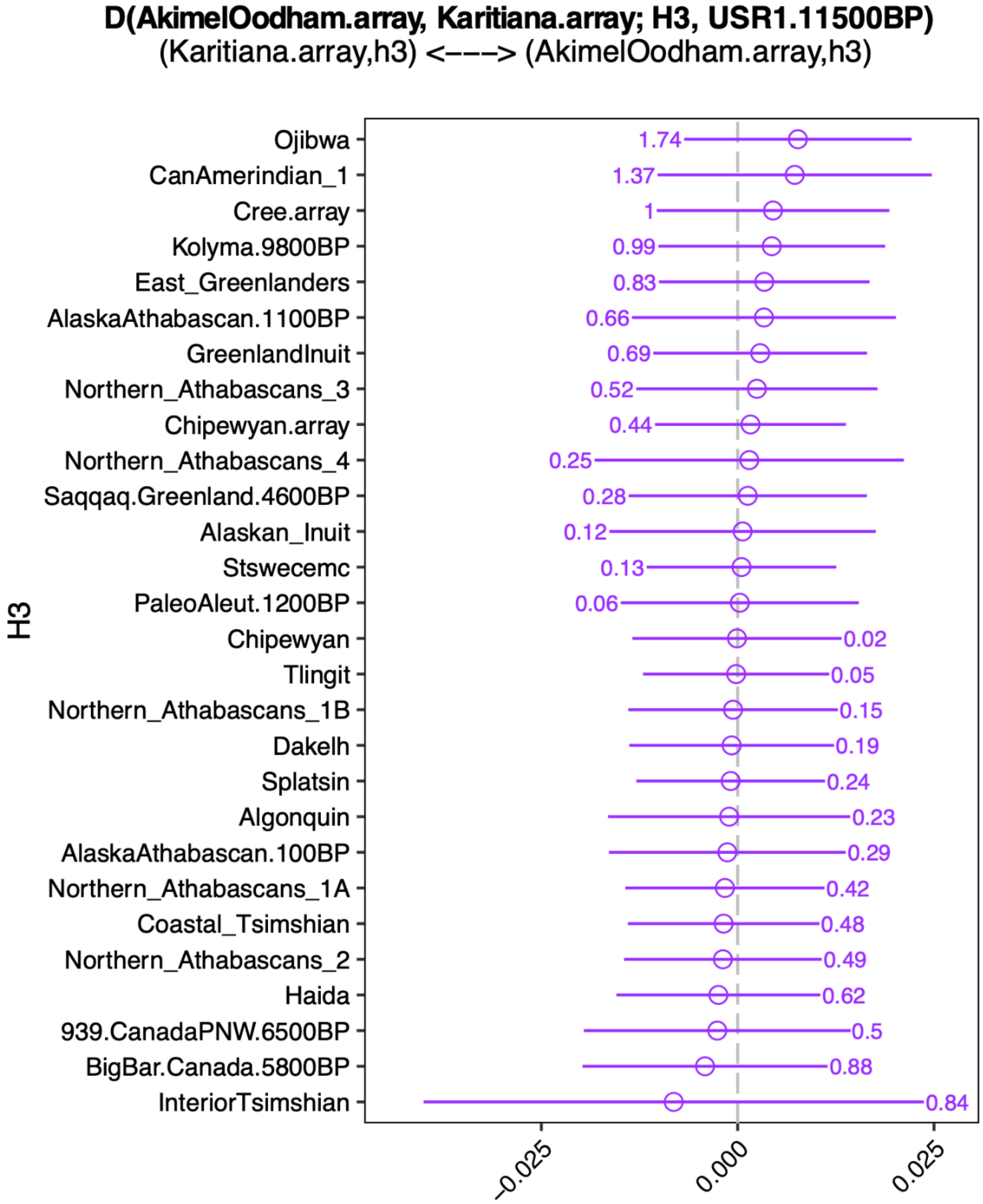

Supplemental Figure S26 *D*-statistics in the format *D*(AkimelOodham, Karitiana, pop, URS1.11500BP) in the Illumina-chip array positions, where pop is all available NNA population. Despite carrying Indigenous American ancestry present upstream to Anzick.12800BP, no sampled NNA group is a good source for this ancestry.

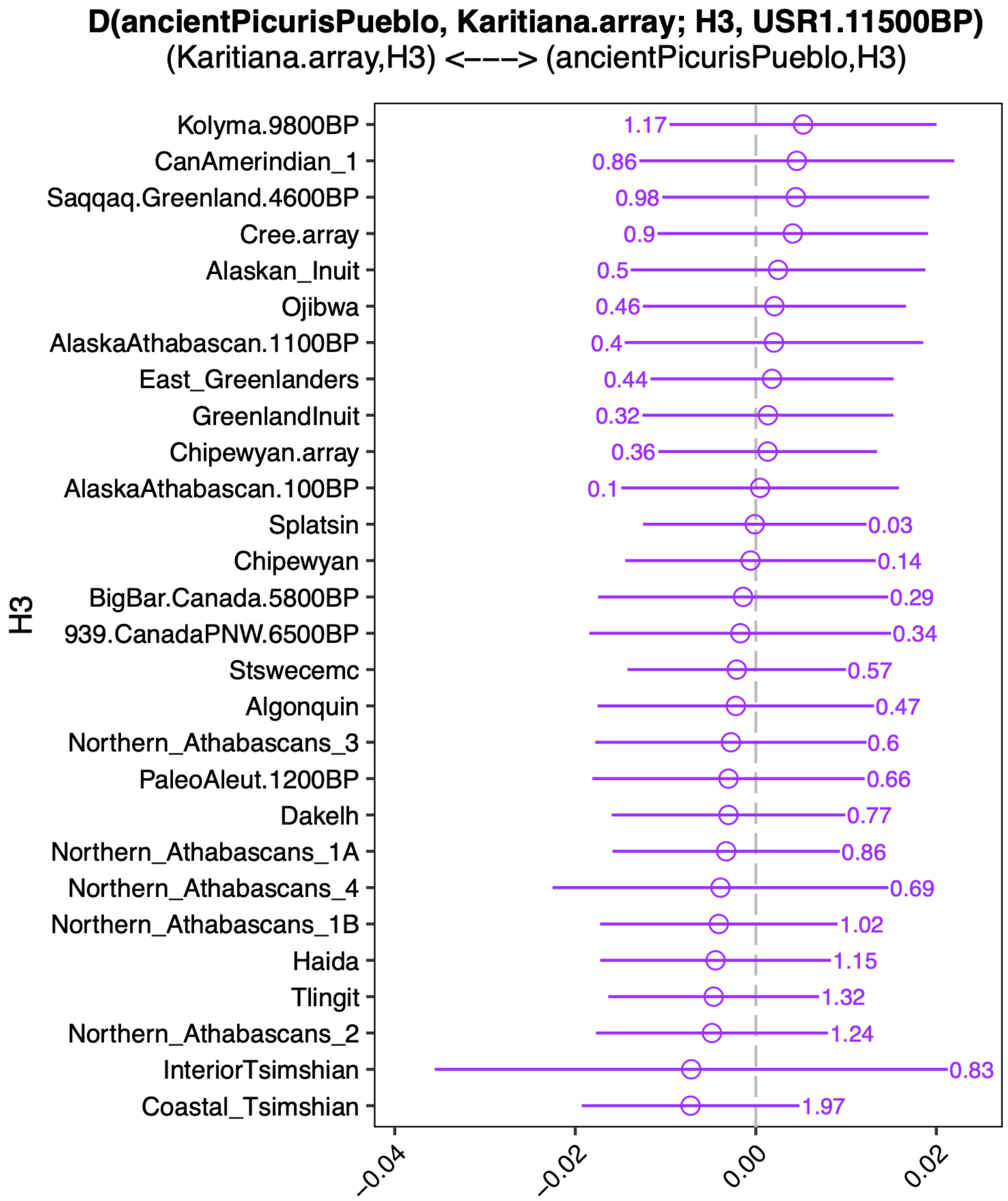

Supplemental Figure S27 *D*-statistics in the format *D*(ancientPicurisPueblo, Karitiana, pop, URS1.11500BP) in the Illumina-chip array positions, where pop is all available NNA populations. Despite carrying Indigenous American ancestry present upstream to Anzick.12800BP, no sampled NNA group is a good source for this ancestry.

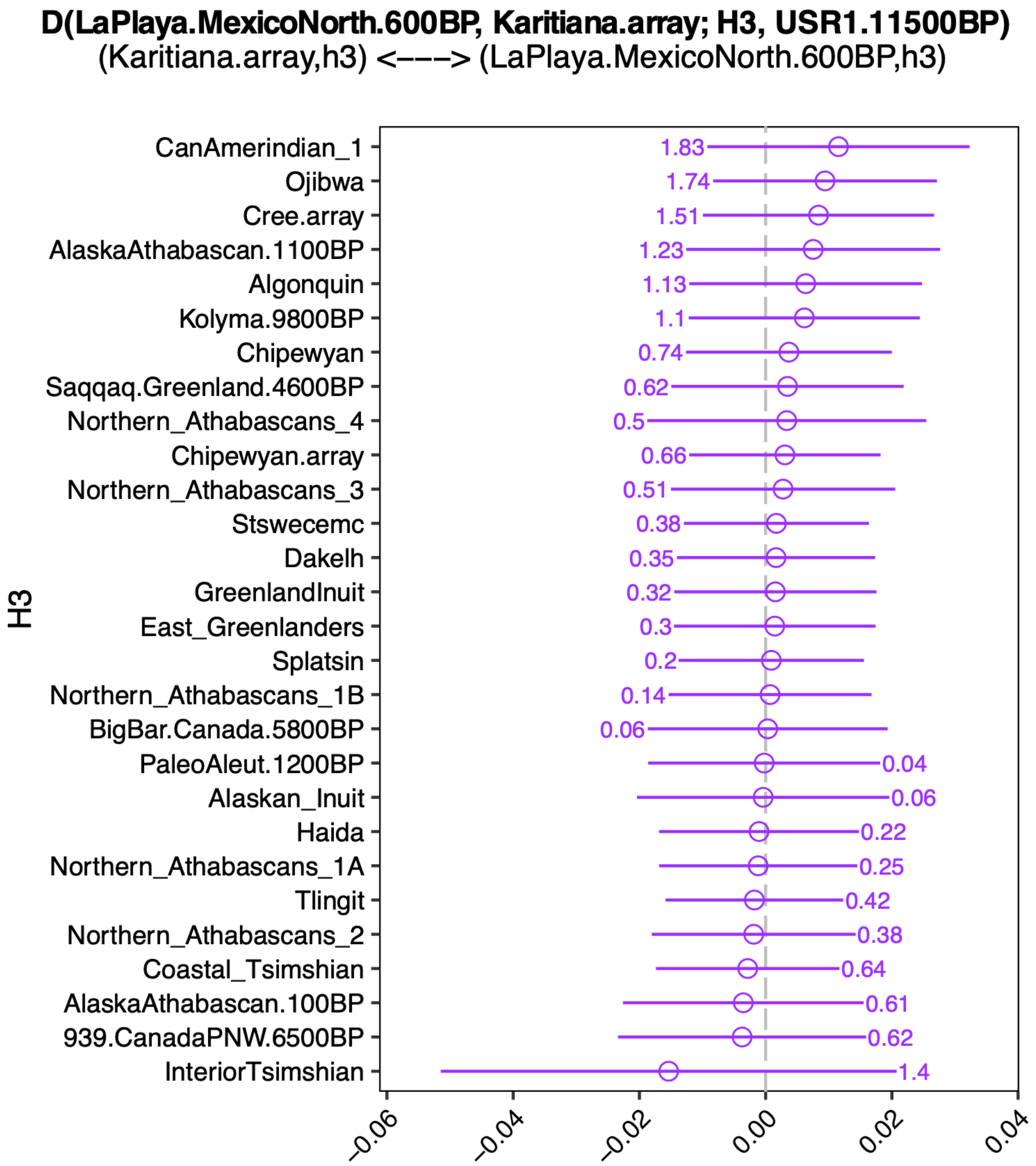

Supplemental Figure S28 *D*-statistics in the format *D*(LaPlaya.600BP, Karitiana, pop, URS1.11500BP) in the Illumina-chip array positions, where pop is all available NNA populations. Despite carrying Indigenous American ancestry present upstream to Anzick.12800BP, no sampled NNA group is a good source for this ancestry.

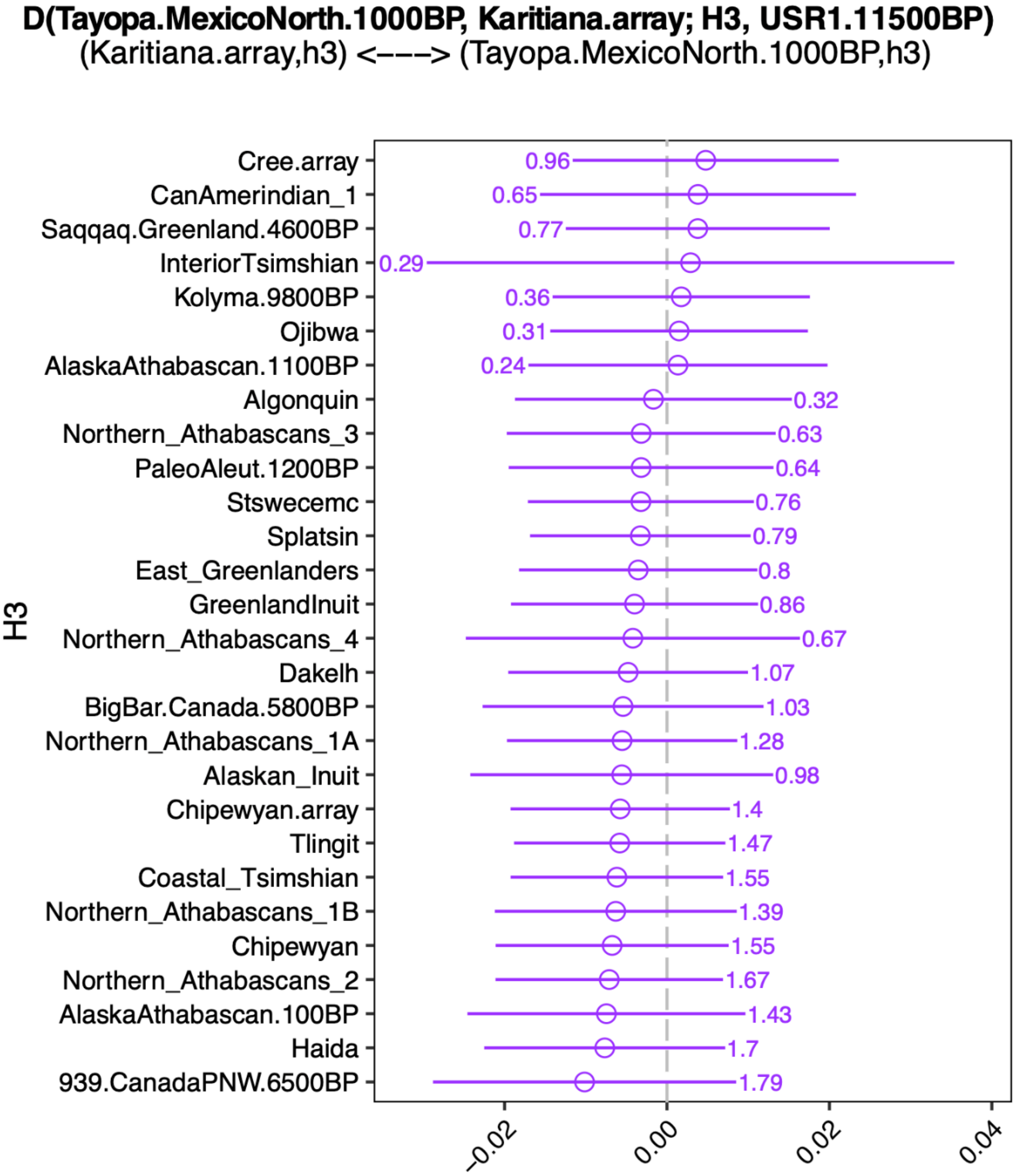

Supplemental Figure S29 *D*-statistics in the format *D*(Tayopa.1000BP, Karitiana, pop, URS1.11500BP) in the Illumina-chip array positions, where pop is all available NNA populations. Despite carrying Indigenous American ancestry present upstream to Anzick.12800BP, no sampled NNA group is a good source for this ancestry.

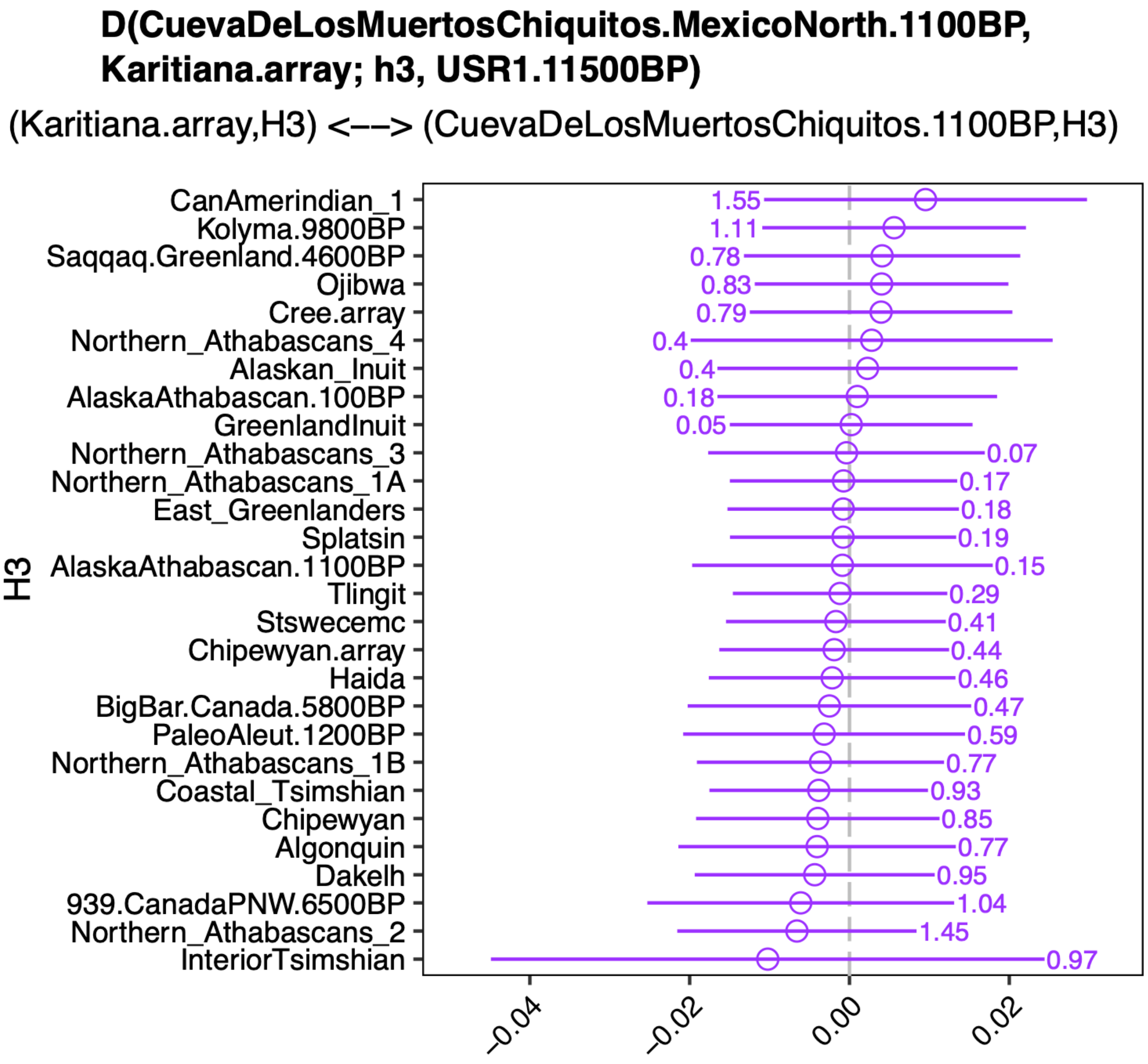

Supplemental Figure S30 *D*-statistics in the format *D*(CuevaDeLosMuertoChiquitos.1100BP, Karitiana, pop, URS1.11500BP) in the Illumina-chip array positions, where pop is all available NNA populations. Despite carrying Indigenous American ancestry present upstream to Anzick.12800BP, no sampled NNA group is a good source for this ancestry.

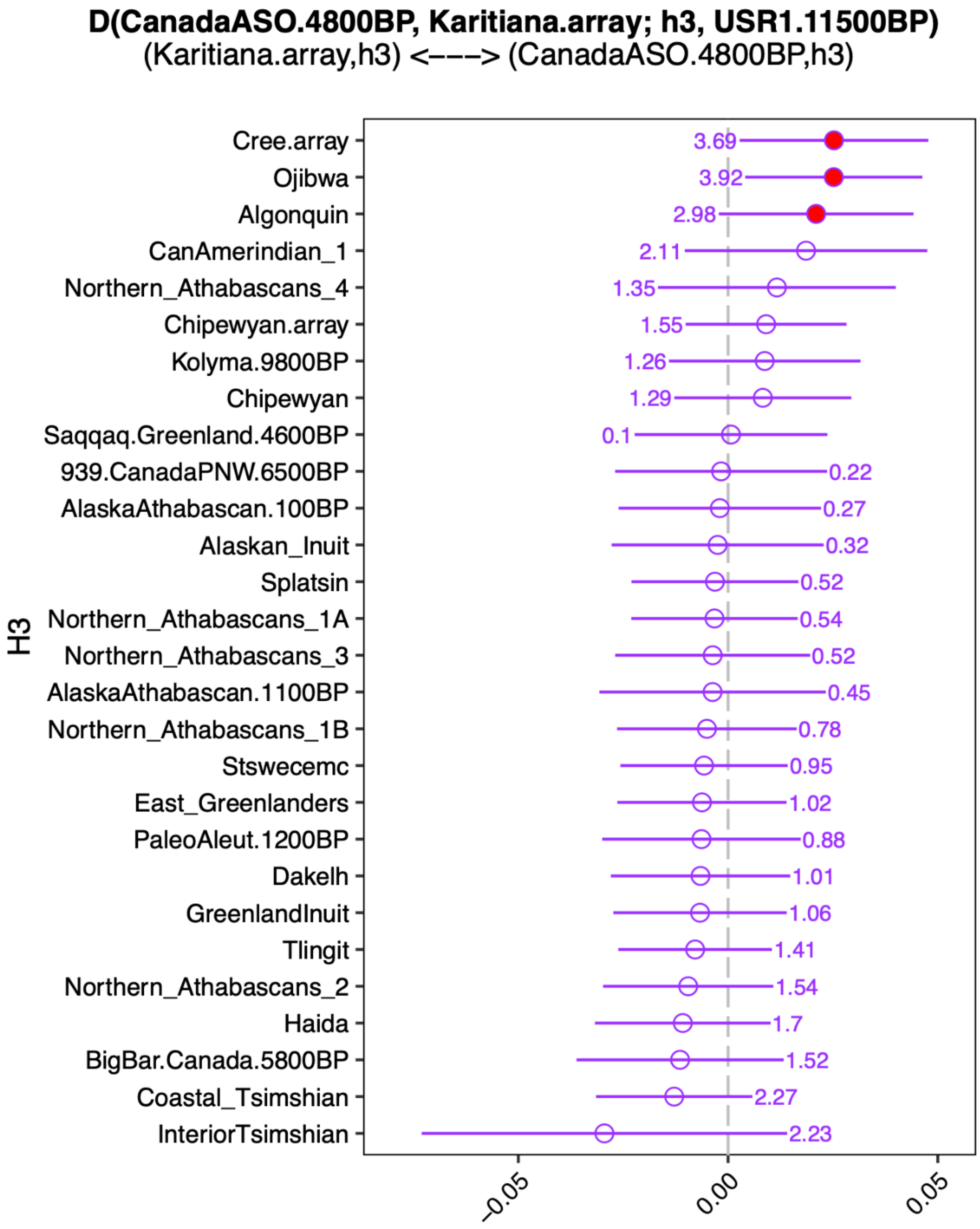

Supplemental Figure S31 *D*-statistics in the format *D*(ASO.4800BP, Karitiana, pop, URS1.11500BP) in the Illumina-chip array positions, where pop is all available NNA populations. In contrast to other North Mexican and Puebloan groups, we obtain significant positive results for Cree, Ojibwa and Algonquin being the source of the non-SNA ancestry in ASO.

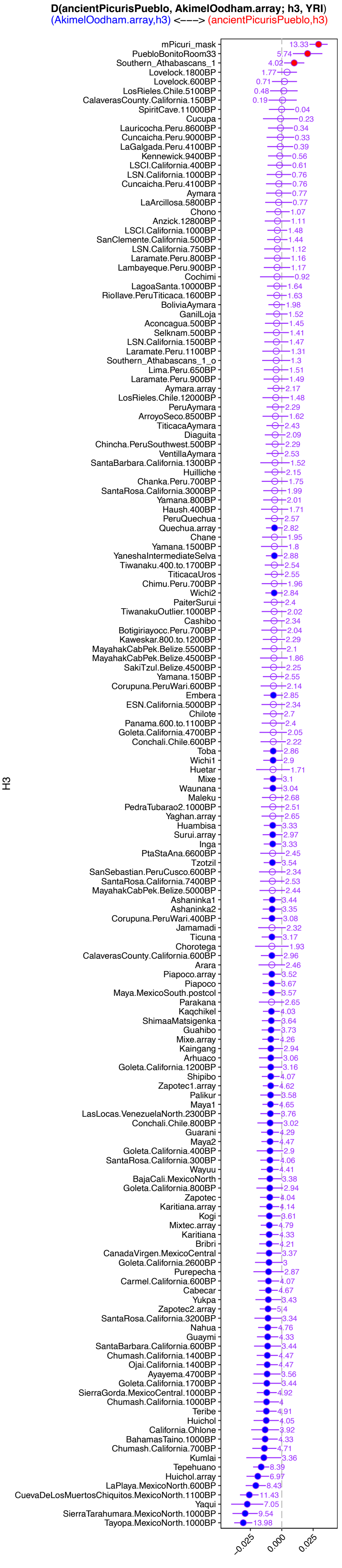
D(ancientPicurisPueblo, AkimelOodham.array; h3, YRI)
(AkimelOodham.array,h3) <---> (ancientPicurisPueblo,h3)
H3
mPicuri_mask 13.33
PuebloBonitoRoom33 5.74
Southern_Athabascans_1 4.02
Lovelock.1800BP 1.77
Lovelock.600BP 0.71
LosRieles.Chile.5100BP 0.48
CalaverasCounty.California.150BP 0.19
SpiritCave.11000BP 0.04
Cucupa 0.23
Lauricocha.Peru.8600BP 0.34
Cuncaicha.Peru.9000BP 0.33
LaGalgada.Peru.4100BP 0.39
Kennewick.9400BP 0.56
LSCI.California.400BP 0.61
LSN.California.1000BP 0.76
Cuncaicha.Peru.4100BP 0.76
Aymara 0.77
LaArcillosa.5800BP 0.77
Chono 1.07
Anzick.12800BP 1.11
LSCI.California.1000BP 1.48
SanClemente.California.500BP 1.44
LSN.California.750BP 1.12
Laramate.Peru.800BP 1.16
Lambayeque.Peru.900BP 1.17
Cochimi 0.92
LagoaSanta.10000BP 1.64
RioIlave.PeruTiticaca.1600BP 1.63
BoliviaAymara 1.98
GanilLoja 1.52
Aconcagua.500BP 1.45
Selknam.500BP 1.41
LSN.California.1500BP 1.47
Laramate.Peru.1100BP 1.31
Southern_Athabascans_1_o 1.3
Lima.Peru.650BP 1.51
Laramate.Peru.900BP 1.49
Aymara.array 2.17
LosRieles.Chile.12000BP 1.48
PeruAymara 2.29
ArroyoSeco.8500BP 1.62
TiticacaAymara 2.43
Diaguita 2.09
Chincha.PeruSouthwest.500BP 2.29
VentillaAymara 2.53
SantaBarbara.California.1300BP 1.52
Huilliche 2.15
Chanka.Peru.700BP 1.75
SantaRosa.California.3000BP 1.99
Yamana.800BP 2.01
Haush.400BP 1.71
PeruQuechua 2.57
Quechua.array 2.82
Chane 1.95
Yamana.1500BP 1.8
YaneshaIntermediateSelva 2.88
Tiwanaku.400.to.1700BP 2.54
TiticacaUros 2.55
Chimu.Peru.700BP 1.96
Wichi2 2.84
PaiterSurui 2.4
TiwanakuOutlier.1000BP 2.02
Cashibo 2.34
Botigiriayocc.Peru.700BP 2.04
Kaweskar.800.to.1200BP 2.29
MayahakCabPek.Belize.5500BP 2.1
MayahakCabPek.Belize.4500BP 1.86
SakiTzul.Belize.4500BP 2.25
Yamana.150BP 2.55
Corupuna.PeruWari.600BP 2.14
Embera 2.85
ESN.California.5000BP 2.34
Chilote 2.7
Panama.600.to.1100BP 2.4
Goleta.California.4700BP 2.05
Conchali.Chile.600BP 2.22
Toba 2.86
Wichi1 2.9
Huetar 1.71
Mixe 3.1
Waunana 3.04
Maleku 2.68
PedraTubarao2.1000BP 2.51
Yaghan.array 2.65
Huambisa 3.33
Surui.array 2.97
Inga 3.33
PtaStaAna.6600BP 2.45
Tzotzil 3.54
SanSebastian.PeruCusco.600BP 2.34
SantaRosa.California.7400BP 2.53
MayahakCabPek.Belize.5000BP 2.44
Ashaninka1 3.44
Ashaninka2 3.35
Corupuna.PeruWari.400BP 3.08
Jamamadi 2.32
Ticuna 3.17
Chorotega 1.93
CalaverasCounty.California.600BP 2.96
Arara 2.46
Piapoco.array 3.52
Piapoco 3.67
Maya.MexicoSouth.postcol 3.57
Parakana 2.65
Kaqchikel 4.03
ShimaaMatsigenka 3.64
Guahibo 3.73
Mixe.array 4.26
Kaingang 2.94
Arhuaco 3.06
Goleta.California.1200BP 3.16
Shipibo 4.07
Zapotec1.array 4.62
Palikur 3.58
Maya1 4.65
LasLocas.VenezuelaNorth.2300BP 3.76
Conchali.Chile.800BP 3.02
Guarani 4.29
Maya2 4.47
Goleta.California.400BP 2.9
SantaRosa.California.300BP 4.06
Wayuu 4.41
BajaCali.MexicoNorth 3.38
Goleta.California.800BP 2.94
Zapotec 4.04
Karitiana.array 4.14
Kogi 3.61
Mixtec.array 4.79
Karitiana 4.33
Bribri 4.21
CanadaVirgen.MexicoCentral 3.37
Goleta.California.2600BP 3
Purepecha 2.87
Carmel.California.600BP 4.07
Cabecar 4.67
Yukpa 3.43
Zapotec2.array 5.4
SantaRosa.California.3200BP 3.34
Nahua 4.76
Guaymi 4.33
SantaBarbara.California.600BP 3.44
Chumash.California.1400BP 4.47
Ojai.California.1400BP 4.47
Ayayema.4700BP 3.56
Goleta.California.1700BP 3.44
SierraGorda.MexicoCentral.1000BP 4.92
Chumash.California.1000BP 4
Teribe 4.91
Huichol 4.05
California.Ohlone 3.92
BahamasTaino.1000BP 4.33
Chumash.California.700BP 4.71
Kumlai 3.36
Tepehuano 8.39
Huichol.array 6.97
LaPlaya.MexicoNorth.600BP 8.43
CuevaDeLosMuertosChiquitos.MexicoNorth.1100BP 11.43
Yaqui 7.05
SierraTarahumara.MexicoNorth.1000BP 9.54
Tayopa.MexicoNorth.1000BP 13.98
-0.025
0.000
0.025

Supplemental Figure S32 D-statistics in the format D(ancientPicurisPueblo, AkimelOodham, pop, Yorubas) in the Illumina-chip array positions, where pop is all available Native American populations, past or present. Red and filled circles denote significant values. The only populations to significant reject the tree with a positive value are PuebloBonitoRoom33, modernPicurisPueblo and Southern_Athabascans_1.

11.2.3. Modelling the ancestry of Southern Athabascans

It is widely believed that Southern Athabascan speakers partly descend from a north-to-south migration that took place between 800 CE and 1600 CE[174]. Early studies, using Y-chromosome polymorphisms, had success identifying typically Northern Athabascan haplogroups among Southern Athabascans[36], that up to this day, seem to be restricted to the Arctic region of North America[35,175]. A distinctive (but not exclusive) genetic feature of most Athabascan speakers is a signal of extra affinity with Far East Siberians and Inuits in comparison to other Native American populations further south[131,144], but this signal is hard to untangle due to back gene flow across Beringia and in Inuits[142,176]. This ancestry, however, seems to be mostly absent among populations from Northeastern North America, such as Ojibwa, Cree, Algonquin[131] and ancient individuals from the area[115,148].

When investigating the ancestry of Southern Athabascans from [131], we found them to fail to be modelled as having a single ancestry source, but rather to be better modelled as a mixture of deep branches of Native American ancestry ("NNA" and "SNA", using the definition in [145]). We then ran all tests in the configuration *D*(Southern_Athabascans_1, SNA, NNA, Yorubas) and *D*(Southern_Athabascans_1, NNA, SNA, Yorubas), because it is an expectation of the *D*-statistic that the population genetically closer to the admixing source would provide the highest value of *D*. We found ancient Picuris Pueblo and Chipewyan to maximise these values (*D(Southern_Athabascans_1, ancientPicurisPueblo, Chipewyan, Yorubas)* = 0.025, $Z = 8.01$; *D(Southern_Athabascans_1, Chipewyan, ancientPicurisPueblo, Yorubas)* = 0.062, $Z = 17.99$), indicating them to be the publicly available most closely related proxies for the ancestry present in Southern Athabascans **(Figure 4, Supplemental Figure S33-S34)**.

Additionally, two of the top three sources for the NNA ancestry are precisely the only two Athabascan individuals East of the Rockies (Chipewyan and 'Northern_Athabascans_3'). This result could support a scenario where the Athabascan migration took an eastern route instead of down an intramountain route. The result of Chipewyan being the best NNA source of Southern Athabascan also explains the tendency of affinity to Far East Siberians and Inuits in those individuals (in the test *D*(Southern_Athabascans_1, SNA, Kolyma.9800BP, Yorubas) – e.g. *D*(Southern_Athabascans_1, ancientPicurisPueblo, Kolyma.9800BP, Yorubas) = 0.010, Z = 3.19), as Chipewyan is one of the NNA populations with highest such affinity (e.g. *D*(SpiritCave.11000BP**,** Chipewyan, Kolyma.9800BP, Yorubas) = -0.019, Z = -3.31).

We then used qpadm[177] to estimate admixture proportions of Southern Athabascans using "ancientPicurisPueblo" and "Chipewyan" as sources and a distinct set of ancestries as references ("Yorubas", "AkimelOodham", "SierraTarahumara.1000BP", "AlaskaAthabascan.1100BP", "Dakelh", "Paiter_Surui"). We found a good fit ($p = 0.34$) for the two-way admixture of 70% Picuris and 30% Chipewyan ancestry, while also obtaining very strong rejections for the alternative single-source models (maximum $p < 0.06 \times 10^{-61}$).

The fact that Southern Athabascans can be modelled as a mixture of a Puebloan and a Northern Athabascan source lets us parsimoniously explain the previous result of present-day Picuris Pueblo members bearing Chipewyan admixture in *D*-statistics (**section 11.2.1.)**, as a recent admixture with the many neighbouring Apachean speaking-groups. Similarly, it also explains the affinity of Far East Siberians and Inuits in present-day, but not ancient Picuris Pueblo (*D*(SpiritCave.11000BP, pop, Kolyma.9800BP, Yorubas) is $D = -0.014$, Z = -2.80 for present-day Picuris but $D = -0.007$, Z = -1.43 for ancient). That no ancient Picuris Pueblo individual bears this admixture, despite a similar sample size, could mean our sampling predates the arrival of this ancestry in the North American Southwest.

In line with the finding that one individual from the Southern Athabascans from [131] was an outlier in both MDS and model clustering analyses, we fail to fit this individual using the same set of populations. When doing the same *D*-statistics based tests using all sampled SNA and NNA populations, we found this individual to share a distinct pattern of SNA and NNA mixture, but with its SNA source being closer to Californian and Mexican populations (Tepehuano, Huichol, Cochimí and ancients Chumash.1000BP and SierraGorda.1000BP) and its NNA to Northeastern North America (ancient ASO.4800BP and Algonquin). In line with this result, when testing for Far East/Inuit affinity (same test as above, using Kolyma.9800BP as a source), we fail to recover any signal (*D*(*SpiritCave.11000BP, Southern_Athabascans_1_o, Kolyma.9800BP, Yorubas*) = -0.002, Z = -0.31).

We also found the whole-genome shotgun Chipewyan from [147] ("Chipewyan") to be a better source for Southern Athabascans than the SNP-array individuals from [164] ("Chipewyan.array"). When running the test *D*(*Chipewyan.array, Chipewyan, Algonquin, Yorubas*) we obtain a very significant positive result (*D* = 0.022, Z = 7.36), showing that this difference is related to admixture taking place with some individuals into the SNP-array dataset, that does not exist in the whole-genome shotgun version of the data. This can also be seen in the K=3 admixture plot (**Supplemental Figure S12**).

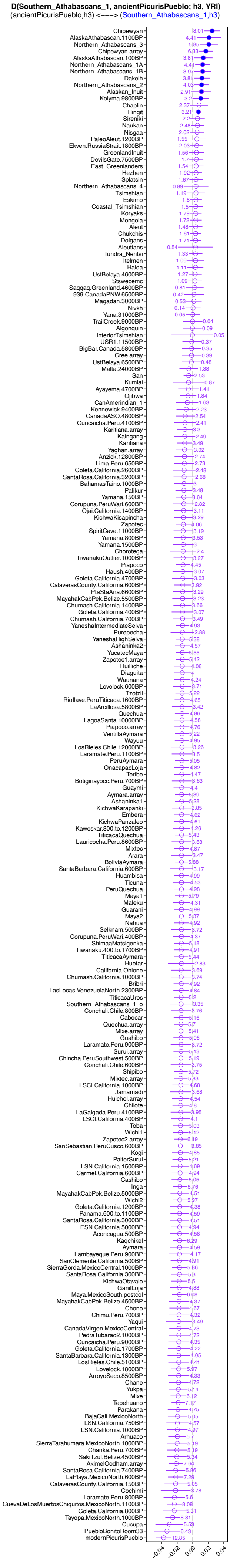
D(Southern_Athabascans_1, ancientPicurisPueblo; h3, YRI)
(ancientPicurisPueblo,h3) <----> (Southern_Athabascans_1,h3)
H3
Chipewyan
AlaskaAthabascan.1100BP
Northern_Athabascans_3
Chipewyan.array
AlaskaAthabascan.100BP
Northern_Athabascans_1A
Northern_Athabascans_1B
Dakelh
Northern_Athabascans_2
Alaskan_Inuit
Kolyma.9800BP
Chaplin
Tlingit
Sireniki
Naukan
Nisgaa
PaleoAleut.1200BP
Ekven.RussiaStrait.1800BP
GreenlandInuit
DevilsGate.7500BP
East_Greenlanders
Hezhen
Splatsin
Northern_Athabascans_4
Tsimshian
Eskimo
Coastal_Tsimshian
Koryaks
Mongola
Aleut
Chukchis
Dolgans
Aleutians
Tundra_Nentsi
Itelmen
Haida
UstBelaya.4600BP
Stswecemc
Saqqaq.Greenland.4600BP
939.CanadaPNW.6500BP
Magadan.3000BP
Nivkh
Yana.31000BP
TrailCreek.9000BP
Algonquin
InteriorTsimshian
USR1.11500BP
BigBar.Canada.5800BP
Cree.array
UstBelaya.6500BP
Malta.24000BP
San
Kumlai
Ayayema.4700BP
Ojibwa
CanAmerindian_1
Kennewick.9400BP
CanadaASO.4800BP
Cuncaicha.Peru.4100BP
Karitiana.array
Kaingang
Karitiana
Yaghan.array
Anzick.12800BP
Lima.Peru.650BP
Goleta.California.2600BP
SantaRosa.California.3200BP
BahamasTaino.1000BP
Palikur
Yamana.150BP
Corupuna.PeruWari.600BP
Ojai.California.1400BP
KichwaKisapincha
Zapotec
SpiritCave.11000BP
Yamana.800BP
Yamana.1500BP
Chorotega
TiwanakuOutlier.1000BP
Piapoco
Haush.400BP
Goleta.California.4700BP
CalaverasCounty.California.600BP
PtaStaAna.6600BP
MayahakCabPek.Belize.5500BP
Chumash.California.1400BP
Goleta.California.400BP
Chumash.California.700BP
YaneshaIntermediateSelva
Purepecha
YaneshaHighSelva
Ashaninka2
YucatecMaya
Zapotec1.array
Huilliche
Diaguita
Waunana
Lovelock.600BP
Tzotzil
Riollave.PeruTiticaca.1600BP
LaArcillosa.5800BP
Quechua
LagoaSanta.10000BP
Piapoco.array
VentillaAymara
Wayuu
LosRieles.Chile.12000BP
Laramate.Peru.1100BP
PeruAymara
OnacapacLoja
Teribe
Botigiriayocc.Peru.700BP
Guaymi
Aymara.array
Ashaninka1
KichwaKarapanki
Embera
KichwaPanzaleo
Kaweskar.800.to.1200BP
TiticacaQuechua
Lauricocha.Peru.8600BP
Mixtec
Arara
BoliviaAymara
SantaBarbara.California.600BP
Huambisa
Ticuna
PeruQuechua
Maya1
Maleku
Guarani
Maya2
Nahua
Selknam.500BP
Corupuna.PeruWari.400BP
ShimaaMatsigenka
Tiwanaku.400.to.1700BP
TiticacaAymara
Huetar
California.Ohlone
Chumash.California.1000BP
Bribri
LasLocas.VenezuelaNorth.2300BP
TiticacaUros
Southern_Athabascans_1_o
Conchali.Chile.800BP
Cabecar
Quechua.array
Mixe.array
Guahibo
Laramate.Peru.900BP
Surui.array
Chincha.PeruSouthwest.500BP
Conchali.Chile.600BP
Shipibo
Mixtec.array
LSCI.California.1000BP
Jamamadi
Huichol.array
Chilote
LaGalgada.Peru.4100BP
LSCI.California.400BP
Toba
Wichi1
Zapotec2.array
SanSebastian.PeruCusco.600BP
Kogi
PaiterSurui
LSN.California.1500BP
Carmel.California.600BP
Cashibo
Inga
MayahakCabPek.Belize.5000BP
Wichi2
Goleta.California.1200BP
Panama.600.to.1100BP
SantaRosa.California.3000BP
ESN.California.5000BP
Aconcagua.500BP
Kaqchikel
Aymara
Lambayeque.Peru.900BP
SanClemente.California.500BP
SierraGorda.MexicoCentral.1000BP
SantaRosa.California.300BP
KichwaOtavalo
GanilLoja
Maya.MexicoSouth.postcol
MayahakCabPek.Belize.4500BP
Chono
Chimu.Peru.700BP
Yaqui
CanadaVirgen.MexicoCentral
PedraTubarao2.1000BP
Cuncaicha.Peru.9000BP
Goleta.California.1700BP
SantaBarbara.California.1300BP
LosRieles.Chile.5100BP
Lovelock.1800BP
ArroyoSeco.8500BP
Chane
Yukpa
Mixe
Tepehuano
Parakana
BajaCali.MexicoNorth
LSN.California.750BP
LSN.California.1000BP
Arhuaco
SierraTarahumara.MexicoNorth.1000BP
Chanka.Peru.700BP
SakiTzul.Belize.4500BP
AkimelOodham.array
SantaRosa.California.7400BP
LaPlaya.MexicoNorth.600BP
CalaverasCounty.California.150BP
Cochimi
Laramate.Peru.800BP
CuevaDeLosMuertosChiquitos.MexicoNorth.1100BP
Goleta.California.800BP
Tayopa.MexicoNorth.1000BP
Cucupa
PuebloBonitoRoom33
modernPicurisPueblo
-0.04
-0.02
0.00
0.02
0.04

Supplemental Figure S33 D-statistics in the format D(Southern_Athabascans_1, ancientPicurisPueblo, pop, Yorubas) in the Illumina-chip array positions, where pop is all available Native American populations, past or present.

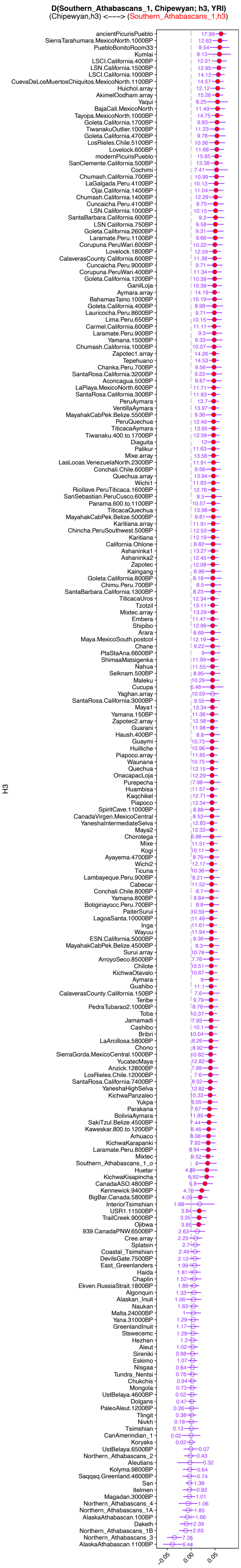

D(Southern_Athabascans_1, Chipewyan; h3, YRI)
(Chipewyan,h3) <----> (Southern_Athabascans_1,h3)
H3
ancientPicurisPueblo 17.99
SierraTarahumara.MexicoNorth.1000BP 12.63
PuebloBonitoRoom33 9.54
Kumlai 8.13
LSCI.California.400BP 12.01
LSN.California.1500BP 12.95
LSCI.California.1000BP 14.13
CuevaDeLosMuertosChiquitos.MexicoNorth.1100BP 14.57
Huichol.array 12.12
AkimelOodham.array 15.26
Yaqui 8.25
BajaCali.MexicoNorth 11.49
Tayopa.MexicoNorth.1000BP 14.75
Goleta.California.1700BP 9.83
TiwanakuOutlier.1000BP 11.23
Goleta.California.4700BP 9.78
LosRieles.Chile.5100BP 10.36
Lovelock.600BP 11.68
modernPicurisPueblo 15.65
SanClemente.California.500BP 13.38
Cochimi 7.41
Chumash.California.700BP 10.99
LaGalgada.Peru.4100BP 10.13
Ojai.California.1400BP 11.04
Chumash.California.1400BP 12.29
Cuncaicha.Peru.4100BP 9.75
LSN.California.1000BP 10.15
SantaBarbara.California.600BP 9.2
LSN.California.750BP 9.58
Goleta.California.2600BP 9.31
Laramate.Peru.1100BP 9.66
Corupuna.PeruWari.600BP 10.22
Lovelock.1800BP 12.59
CalaverasCounty.California.600BP 11.38
Cuncaicha.Peru.9000BP 9.71
Corupuna.PeruWari.400BP 11.34
Goleta.California.1200BP 10.39
GanilLoja 10.39
Aymara.array 14.19
BahamasTaino.1000BP 10.19
Goleta.California.400BP 8.98
Lauricocha.Peru.8600BP 9.71
Lima.Peru.650BP 10.15
Carmel.California.600BP 11.11
Laramate.Peru.900BP 9.5
Yamana.1500BP 9.33
Chumash.California.1000BP 10.07
Zapotec1.array 14.26
Tepehuano 14.53
Chanka.Peru.700BP 9.56
SantaRosa.California.3200BP 9.22
Aconcagua.500BP 9.67
LaPlaya.MexicoNorth.600BP 11.71
SantaRosa.California.300BP 11.83
PeruAymara 13.7
VentillaAymara 13.97
MayahakCabPek.Belize.5500BP 9.36
PeruQuechua 12.49
TiticacaAymara 13.95
Tiwanaku.400.to.1700BP 12.59
Diaguita 12
Palikur 11.63
Mixe.array 13.56
LasLocas.VenezuelaNorth.2300BP 11.91
Conchali.Chile.600BP 9.56
Quechua.array 13.94
Wichi1 11.83
RioIlave.PeruTiticaca.1600BP 12.76
SanSebastian.PeruCusco.600BP 8.5
Panama.600.to.1100BP 10.57
TiticacaQuechua 13.98
MayahakCabPek.Belize.5000BP 9.81
Karitiana.array 11.91
Chincha.PeruSouthwest.500BP 12.53
Karitiana 12.19
California.Ohlone 8.82
Ashaninka1 13.27
Ashaninka2 12.45
Zapotec 12.08
Kaingang 8.96
Goleta.California.800BP 8.18
Chimu.Peru.700BP 8.5
SantaBarbara.California.1300BP 8.23
TiticacaUros 12.34
Tzotzil 13.11
Mixtec.array 13.29
Embera 11.47
Shipibo 12.99
Arara 8.69
Maya.MexicoSouth.postcol 12.19
Chane 9.22
PtaStaAna.6600BP 9
ShimaaMatsigenka 11.99
Nahua 11.55
Selknam.500BP 8.85
Maleku 10.29
Cucupa 6.48
Yaghan.array 10.59
SantaRosa.California.3000BP 9.55
Maya1 13.34
Yamana.150BP 11.38
Zapotec2.array 12.58
Guarani 11.98
Haush.400BP 8.8
Guaymi 10.73
Huilliche 10.96
Piapoco.array 11.85
Waunana 10.75
Quechua 12.15
OnacapacLoja 12.29
Purepecha 7.98
Huambisa 11.57
Kaqchikel 12.71
Piapoco 12.34
SpiritCave.11000BP 8.88
CanadaVirgen.MexicoCentral 8.53
YaneshaIntermediateSelva 12.83
Maya2 12.33
Chorotega 6.98
Mixe 11.51
Kogi 10.11
Ayayema.4700BP 8.76
Wichi2 12.17
Ticuna 10.36
Lambayeque.Peru.900BP 8.21
Cabecar 11.52
Conchali.Chile.800BP 8.7
Yamana.800BP 9.84
Botigiriayocc.Peru.700BP 8.8
PaiterSurui 10.59
LagoaSanta.10000BP 11.49
Inga 11.61
Wayuu 11.94
ESN.California.5000BP 9.36
MayahakCabPek.Belize.4500BP 8.3
Surui.array 10.78
ArroyoSeco.8500BP 7.76
Chilote 10.51
KichwaOtavalo 10.67
Aymara 9
Guahibo 11.1
CalaverasCounty.California.150BP 7.6
Teribe 9.79
PedraTubarao2.1000BP 8.76
Toba 10.37
Jamamadi 7.93
Cashibo 10.1
Bribri 10.04
LaArcillosa.5800BP 8.26
Chono 8.92
SierraGorda.MexicoCentral.1000BP 10.82
YucatecMaya 12.82
Anzick.12800BP 7.99
LosRieles.Chile.12000BP 7.6
SantaRosa.California.7400BP 8.52
YaneshaHighSelva 12.82
KichwaPanzaleo 10.33
Yukpa 8.05
Parakana 7.67
BoliviaAymara 11.85
SakiTzul.Belize.4500BP 7.44
Kaweskar.800.to.1200BP 8.46
Arhuaco 8.08
KichwaKarapanki 7.92
Laramate.Peru.800BP 6.94
Mixtec 9.52
Southern_Athabascans_1_o 6
Huetar 4.89
KichwaKisapincha 6.92
CanadaASO.4800BP 5.8
Kennewick.9400BP 4.78
BigBar.Canada.5800BP 4.09
InteriorTsimshian 1.88
USR1.11500BP 3.84
TrailCreek.9000BP 3.35
Ojibwa 3.88
939.CanadaPNW.6500BP 2.63
Cree.array 2.25
Splatsin 2.7
Coastal_Tsimshian 2.49
DevilsGate.7500BP 2.13
East_Greenlanders 1.99
Haida 1.81
Chaplin 1.52
Ekven.RussiaStrait.1800BP 1.88
Algonquin 1.33
Alaskan_Inuit 1.06
Naukan 1.63
Malta.24000BP 1
Yana.31000BP 1.29
GreenlandInuit 1.17
Stswecemc 1.28
Hezhen 1.2
Aleut 1.02
Sireniki 0.88
Eskimo 1.07
Nisgaa 0.84
Tundra_Nentsi 0.76
Chukchis 0.94
Mongola 0.73
UstBelaya.4600BP 0.52
Dolgans 0.47
PaleoAleut.1200BP 0.26
Tlingit 0.38
Nivkh 0.18
Tsimshian 0.13
CanAmerindian_1 0.02
Koryaks 0.02
UstBelaya.6500BP -0.07
Northern_Athabascans_2 -0.43
Aleutians -0.32
Kolyma.9800BP -0.64
Saqqaq.Greenland.4600BP -0.74
San -1.38
Itelmen -0.82
Magadan.3000BP -1.01
Northern_Athabascans_4 -1.06
Northern_Athabascans_1A -1.85
AlaskaAthabascan.100BP -1.66
Dakelh -2.39
Northern_Athabascans_1B -2.63
Northern_Athabascans_3 -7.35
AlaskaAthabascan.1100BP -6.44
-0.05
0.00
0.05

Supplemental Figure S34 D-statistics in the format D(Southern_Athabascans_1, Chipewyan, pop, Yorubas) in the Illumina-chip array positions, where pop is all available Native American populations, past or present.

12. Founder population age and bottleneck estimation

Using the correlation between allele sharing in pairs of individuals from the same population ASCEND[178] estimates the age of founder populations (Tf) and the strength of the bottleneck (If). Using the imputed diploid genotypes for the 1240K positions and ancient individuals for Picuris Pueblo, we obtain a significant fit for a bottleneck (28.2 - 31.7%, NRMSD: 0.014) taking place only between 180.3 and 208.8 generations before sampling or, at 28 years per generation, around 5,700 to 6,400 before present. While significant, this estimate is very close to the simulated limit of the method, and therefore should be interpreted with caution. Crucially, however, we detect no recent bottleneck in ancient Picuris Pueblo history, therefore providing further evidence against a supposed demographic collapse taking around 1,100 CE.

13. Estimating the timing of admixture events in present-day Picuris Pueblo

We used DATES[179] v. 4010 to estimate the timing of both Athabascan and European admixture in present-day individuals from Picuris Pueblo using imputed diploid genotypes. As we expect both those events to be recent and, therefore, admixture LD blocks to be large, we increased the maximum allowed distance between SNPs to 0.5 Morgans (--maxdis: 0.5); elsewhere, we used default settings. We considered results significant following [179], namely, Z-score above 2, Normalized Root Mean Square Distribution (NRMSD) score to be below 0.7 and 2 standard errors not overlapping with zero. Due to the low sample size of Chipewyan (n=2), we pooled those individuals with 2 other Northern Athabascans (Dakelh) from British Columbia.

We estimate the date of the Athabascan admixture into present-day Picuris Pueblo to have taken place 19.871 (+/- 6.439) generations or, at 28 years per generation, 556 (+/- 180) years ago (Z-score: 2.574; NRMDS: 0.204, **Supplemental Figure S35**). The large confidence interval can be both attributed to the overall low sample size or the low genetic differentiation between the two sources, and the 95% confidence interval range of 203-909 years ago does not allow us to place the admixture confidently on having taking place before or after European arrival.

Conversely, using IBS as a proxy for European ancestry, we date European admixture to have taken place more recently, at 9.986 (+/- 1.077) generations or 279 (+/- 30) years ago (Z-score: 9.275; NRMDS: 0.049, **Supplemental Figure S35**). The 95% confidence interval of 220-338 years ago is consistent with it taking place on the period of more intense settlement of the region by Spanish colonists, following Juan de Oñate settlement in the region in 1598.

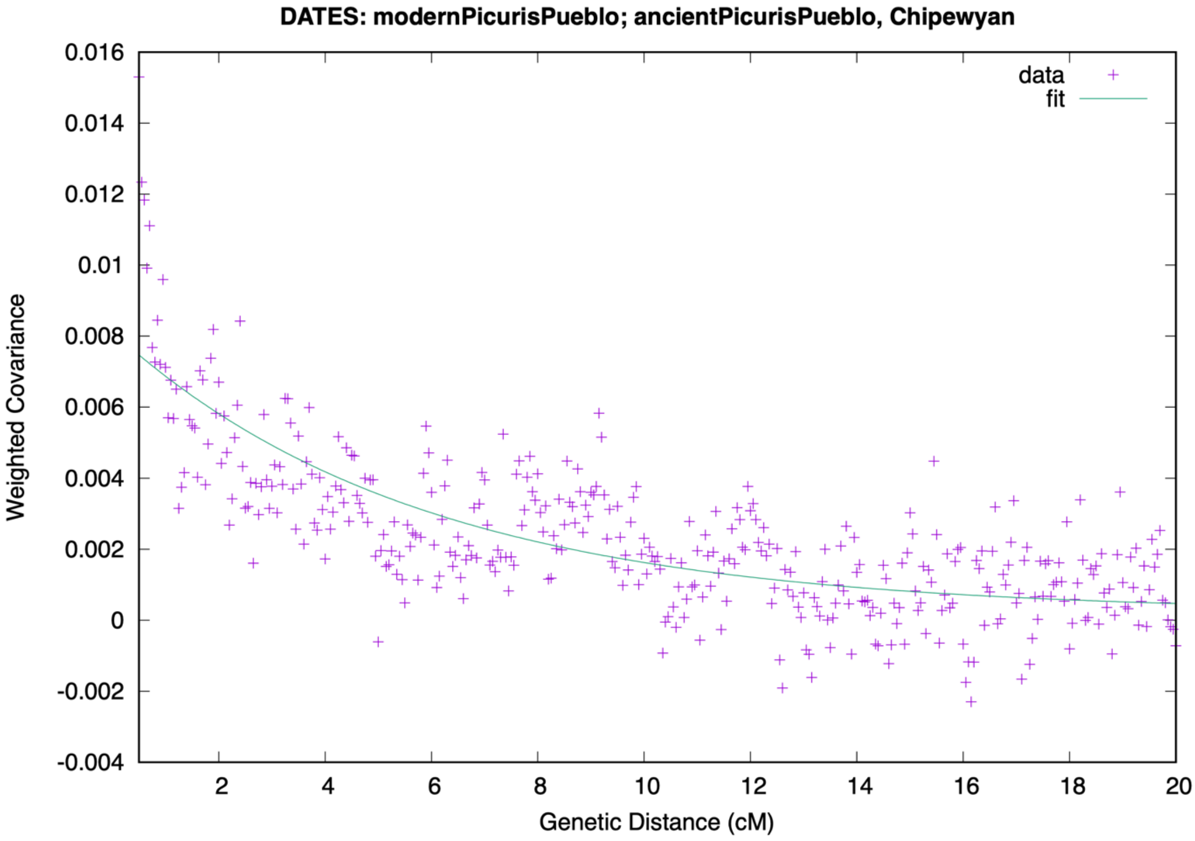
DATES: modernPicurisPueblo; ancientPicurisPueblo, Chipewyan
data
fit
Weighted Covariance
Genetic Distance (cM)
0.016
0.014
0.012
0.01
0.008
0.006
0.004
0.002
0
-0.002
-0.004
2
4
6
8
10
12
14
16
18
20

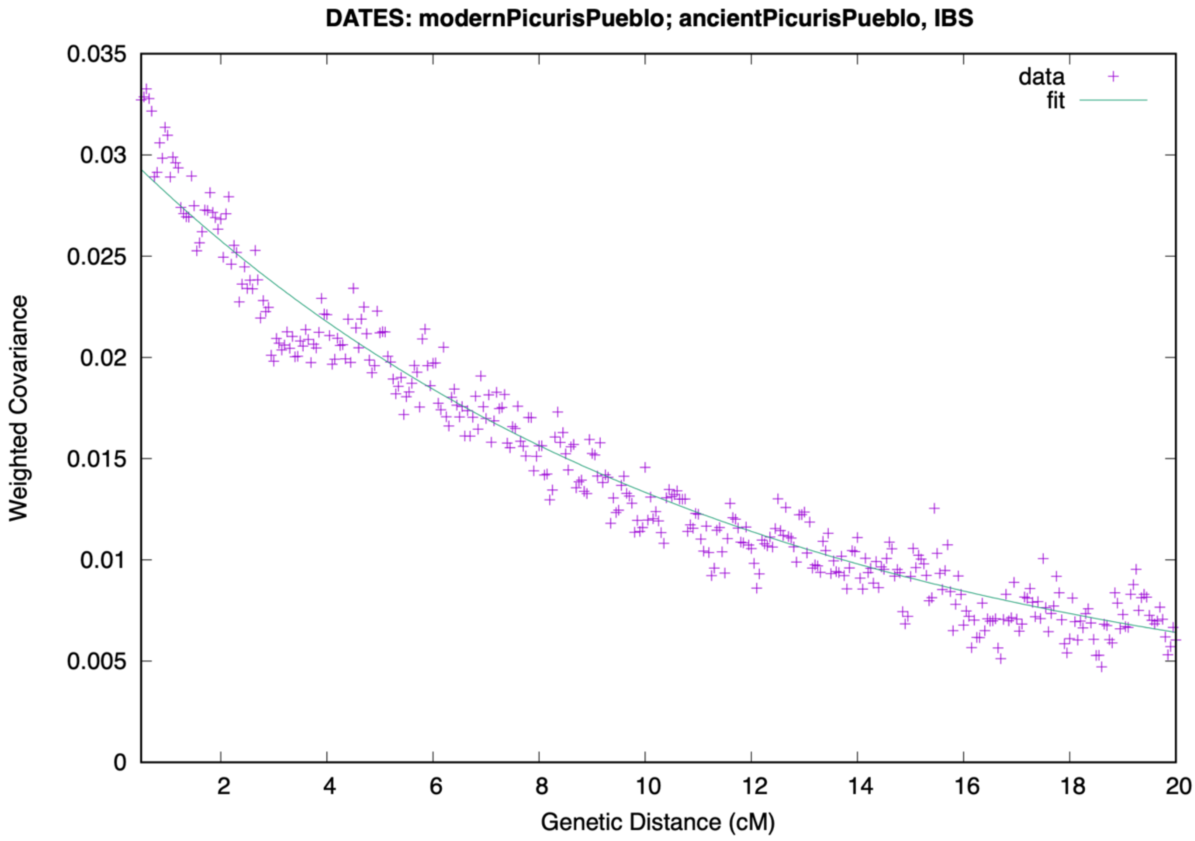
DATES: modernPicurisPueblo; ancientPicurisPueblo, IBS
data
fit
Weighted Covariance
Genetic Distance (cM)
0.035
0.03
0.025
0.02
0.015
0.01
0.005
0
2
4
6
8
10
12
14
16
18
20

Supplemental Figure S35 DATES output for timing admixture events in present-day individuals from Picuris Pueblo.

14. Identity-by-Descent (IBD) segment sharing

14.1. Detection and validation of Identical-by-Descent (IBD) segments

Haplotypes are termed to be identical by descent (henceforth, IBD) if it can be shown they were inherited from a common ancestor. Because it is possible to identify genomic segments that are likely to be IBD, it is possible to infer sharing of genealogical ancestors between two individuals using only sequence information. While, in theory, all humans descend from a set of common ancestors that lived at some point in the past, in practice, recombination and mutation events break down and modify those haplotypes making them difficult to be identified. However, individuals from the same area may share not one, but likely many, much more recent, common ancestors. This, together with comprehensive reference panels, has been shown to allow the detection of IBD segments between individuals in Eurasia spanning the last 30 kya[128]. A special case of IBD sharing is when this happens within a diploid individual. These regions, termed homozygous by descent (HBD), indicate some level of parental relatedness, and can be used to estimate population size and inbreeding.

We used IBDseq[180] in all genomes passing all filters, restricting to polymorphisms with both reference and panel allele frequency above 0.01. We only considered segments with a LOD score higher than 3, and applied a length threshold of 2 cM for IBD. We removed regions of excess IBD sharing following [128,180], and segments that became shorter than the length threshold after this filter. We used similar parameters for analysing HBD segments, but instead restricted to transversion polymorphisms and only removed segments shorter than 0.5 cM. The total amount of HBD per individual can be found in **(Supplemental Figure S36)**.

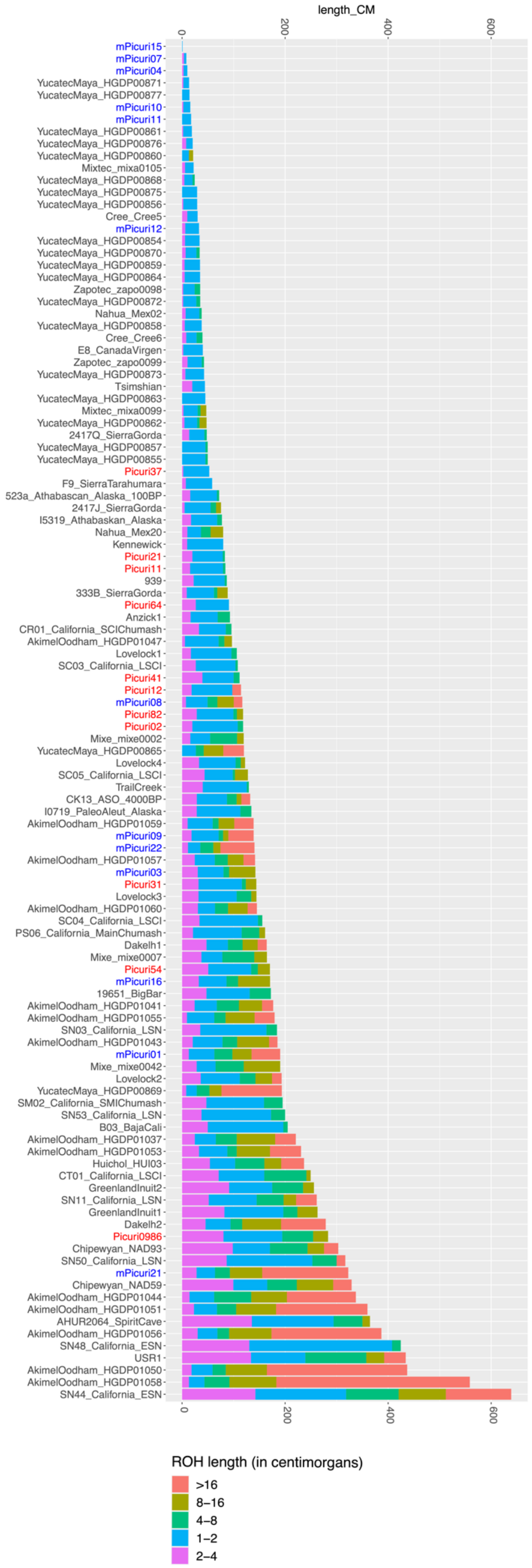
length_CM
0
200
400
600
mPicuri15
mPicuri07
mPicuri04
YucatecMaya_HGDP00871
YucatecMaya_HGDP00877
mPicuri10
mPicuri11
YucatecMaya_HGDP00861
YucatecMaya_HGDP00876
YucatecMaya_HGDP00860
Mixtec_mixa0105
YucatecMaya_HGDP00868
YucatecMaya_HGDP00875
YucatecMaya_HGDP00856
Cree_Cree5
mPicuri12
YucatecMaya_HGDP00854
YucatecMaya_HGDP00870
YucatecMaya_HGDP00859
YucatecMaya_HGDP00864
Zapotec_zapo0098
YucatecMaya_HGDP00872
Nahua_Mex02
YucatecMaya_HGDP00858
Cree_Cree6
E8_CanadaVirgen
Zapotec_zapo0099
YucatecMaya_HGDP00873
Tsimshian
YucatecMaya_HGDP00863
Mixtec_mixa0099
YucatecMaya_HGDP00862
2417Q_SierraGorda
YucatecMaya_HGDP00857
YucatecMaya_HGDP00855
Picuri37
F9_SierraTarahumara
523a_Athabascan_Alaska_100BP
2417J_SierraGorda
I5319_Athabaskan_Alaska
Nahua_Mex20
Kennewick
Picuri21
Picuri11
939
333B_SierraGorda
Picuri64
Anzick1
CR01_California_SCIChumash
AkimelOodham_HGDP01047
Lovelock1
SC03_California_LSCI
Picuri41
Picuri12
mPicuri08
Picuri82
Picuri02
Mixe_mixe0002
YucatecMaya_HGDP00865
Lovelock4
SC05_California_LSCI
TrailCreek
CK13_ASO_4000BP
I0719_PaleoAleut_Alaska
AkimelOodham_HGDP01059
mPicuri09
mPicuri22
AkimelOodham_HGDP01057
mPicuri03
Picuri31
Lovelock3
AkimelOodham_HGDP01060
SC04_California_LSCI
PS06_California_MainChumash
Dakelh1
Mixe_mixe0007
Picuri54
mPicuri16
19651_BigBar
AkimelOodham_HGDP01041
AkimelOodham_HGDP01055
SN03_California_LSN
AkimelOodham_HGDP01043
mPicuri01
Mixe_mixe0042
Lovelock2
YucatecMaya_HGDP00869
SM02_California_SMIChumash
SN53_California_LSN
B03_BajaCali
AkimelOodham_HGDP01037
AkimelOodham_HGDP01053
Huichol_HUI03
CT01_California_LSCI
GreenlandInuit2
SN11_California_LSN
GreenlandInuit1
Dakelh2
Picuri0986
Chipewyan_NAD93
SN50_California_LSN
mPicuri21
Chipewyan_NAD59
AkimelOodham_HGDP01044
AkimelOodham_HGDP01051
AHUR2064_SpiritCave
AkimelOodham_HGDP01056
SN48_California_ESN
USR1
AkimelOodham_HGDP01050
AkimelOodham_HGDP01058
SN44_California_ESN
0
200
400
600
ROH length (in centimorgans)
>16
8–16
4–8
1–2
2–4

Supplemental Figure S36 Total sum of regions identified as Homozygous by Descent in the analysed imputed whole-genomes. Ancient Picuris individuals are highlighted in red.

14.2. Average IBD sharing profile with other populations

Sharing of IBD segments are indicative of shared ancestors and therefore a shared population history. To obtain a population estimate of present-day Picuris Pueblo IBD sharing with other groups, we followed the approach in [181] and averaged the total IBD per size bins (**Figure 3**). We found an overwhelming signal of population continuity locally between ancient and present-day Picuris, with multiple, long shared haplotypes (>32 cM) among them.

15. Estimation of population demography in recent time

We used ibdNe[182] to estimate effective population size using the IBD segments estimated from 11 unrelated ancient Picuris individuals at sufficient depth of coverage for imputation, using a 2 centimorgans threshold **(Supplemental Figure S37)**, and found no signal of recent population collapse, but rather a recent population expansion. Because a recent study have shown that ibdNe can underestimate recent effective population size at low sample size, we also used a Linkage Disequilibrium (LD) based estimate of population demography (HapNe-LD)[183], and while we could not replicate the recent expansion, we obtained a constant population size and no signal of any recent demographical change in the recent past of Picuris Pueblo (**Supplemental Figure S38**).

We calculated the harmonic mean of the effective population size (Ne) over the last 10 generations and obtained compatible values with both methods, 3,190 (95% CI: 1,756 - 8,296) using ibdNe and 5,300 (97.5% CI: 4,948-5,904) using HapNe-LD.

In addition, we also used HapNe-LD on the present-day Picuris individuals, and we recover a strong signal of population collapse taking place at the time of the European arrival (around 20 generations ago), but concordantly obtain no population bottleneck before that (**Figure 3**). To estimate the rate of population decline following European arrival, we calculated the harmonic mean of Ne for 20 to 30 generations ago and of the last 5 estimates. Strikingly, the estimate for population size 20 to 30 generations ago is very similar with the one from ancient Picuris individuals, which would have lived at this moment in time (5,480, 97.5% CI: 4,292-6,679). The mean for the last five generations, however, is 805 (97.5% CI: 643-1,028), which represents an 85% population decline. Because admixture with European and African sources increase the Ne, we consider this value of 85% represents an upper bound for the real population decline rate.

To test the efficacy of the method in other populations with a well-documented recent population collapse, we applied the same methodology for present-day Akimel O'odham, Karitiana and Paiter Suruí individuals from [117] and for historical Maya individuals from [158] (**Supplemental Figure S39-S42**). In all cases we obtain both a pre-colonial effective population size estimate (1,558, 1,254, 1,524 and 13,172) and a subsequent depopulation rate (81%, 91%, 93% and 91%, respectively) congruent with previous literature estimates[184]. This suggests the method is robust for properly detecting signals of recent population contraction.

In conclusion, we used three different methods (allele age, IBD-based and LD-based demography estimate) but fail to detect any signal of population decline taking place in Picuris Pueblo before European arrival. This strongly suggest a thriving population up until Colonial times and that the depopulation of some sites in the Southwest likely represent a population reshuffling in the landscape, rather than some depopulation event.

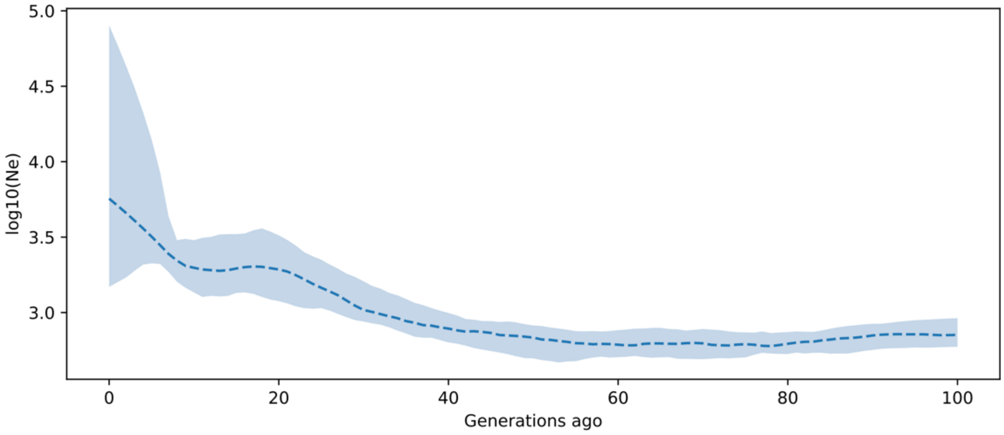

Supplemental Figure S37 Demographic trajectory estimated by ibdNe using ancient Picuris Pueblo individuals.

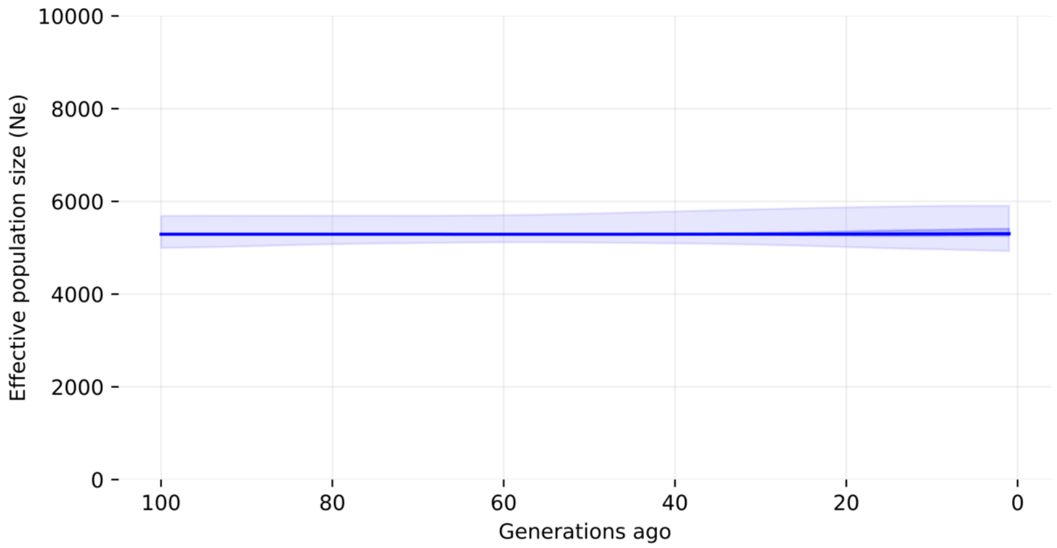

Supplemental Figure S38 Demographic trajectory estimated by HapNe-LD using ancient Picuris Pueblo individuals.

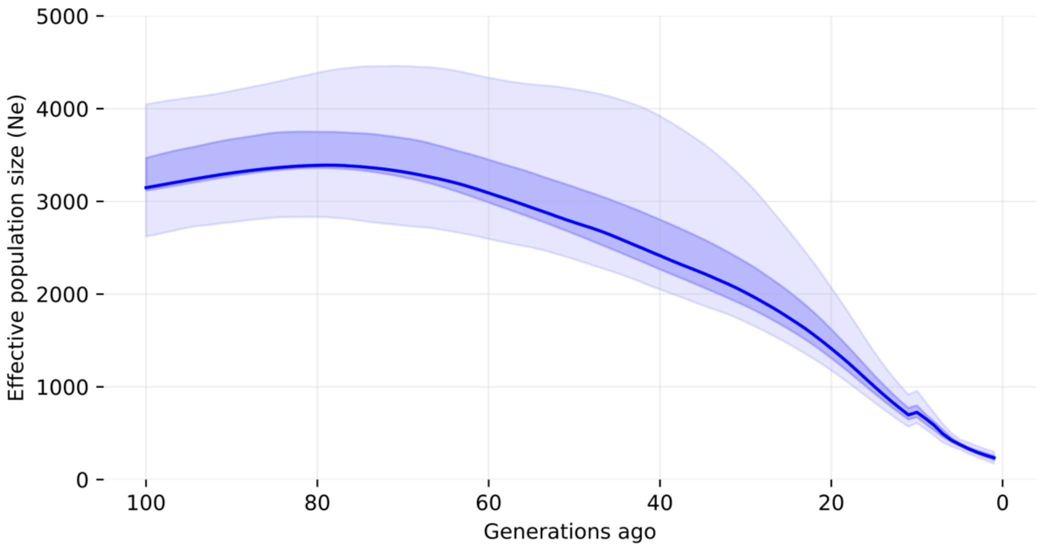

Supplemental Figure S39 Demographic trajectory estimated by HapNe-LD using present-day Akimel O'odham individuals.

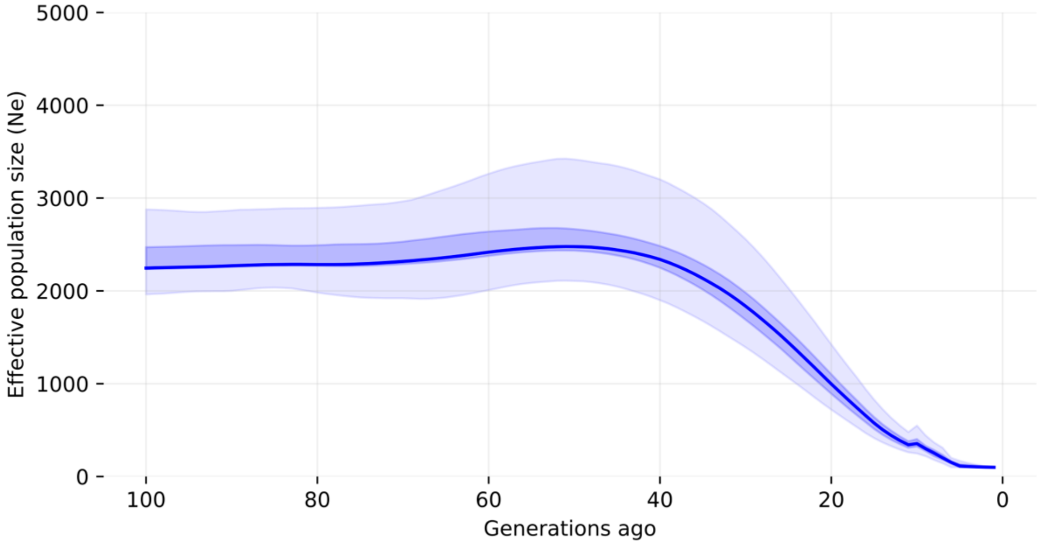

Supplemental Figure S40 Demographic trajectory estimated by HapNe-LD using present-day Karitiana individuals.

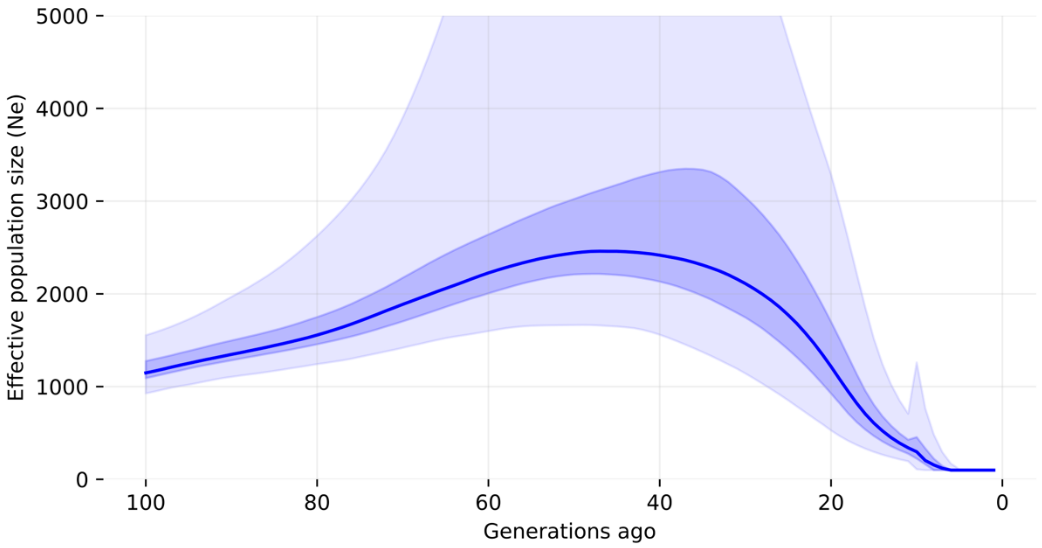

Supplemental Figure S41 Demographic trajectory estimated by HapNe-LD using present-day Paiter Suruí individuals.

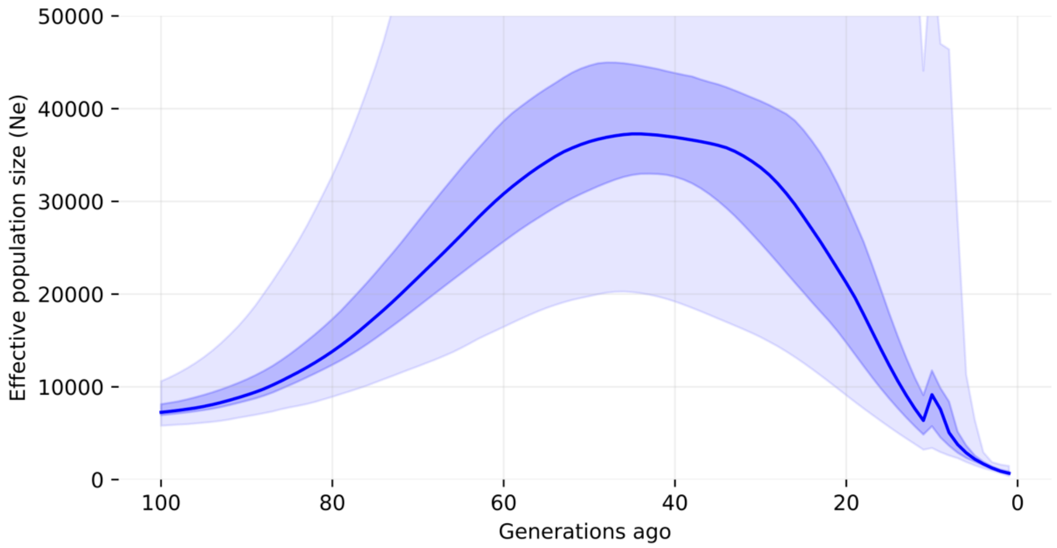

Supplemental Figure S42 Demographic trajectory estimated by HapNe-LD using historical post-colonial Campeche Mayan individuals.

16. Pairwise branch lengths

We used the method described in [145] to measure the amount of drift that leads to each one of the ancient Picuris individuals after splitting from a set of reference genomes (including present-day Picuris). This method was originally devised as a test for direct ancestry – corresponding to the case of no drift along one of the two branches. Given the sampling age of the ancient Picuris, we do not expect the branches leading to any of them to be zero. Instead, we use these estimates to explore whether the ancient Picuris diverged from present-day Picuris more recently than they diverged from other populations in the Americas and the rest of the world. We used imputed diploid genotypes, restricting to positions passing filters described Section 9.1.2. and using a 5% reference panel allele frequency threshold for inclusion. Additionally, we set to missing positions with low genotype probability score (<0.95) per individual. We restricted this analysis to sites that are variable in the African individuals that are included in the 1000 Genomes Project to minimise the presence of mutations that arose after the split between the tested genomes and excluded transition polymorphisms to minimise error deriving from post-mortem ancient DNA modifications.

We estimated the shortest lengths for the branches leading to the ancient Picuris individuals whenever we considered their split from present-day Picuris carrying low European and Athabascan admixture (**Supplemental Figure S43**). Furthermore, we observed that these values increased as we compared the ancient Picuris with individuals from populations sampled farther away from the U.S. Southwest. These results confirm our allele frequency- and IBD-based results showing that present-day Picuris are most closely related to the ancient Picuris, thus supporting population genetic in the last 1,000 years.

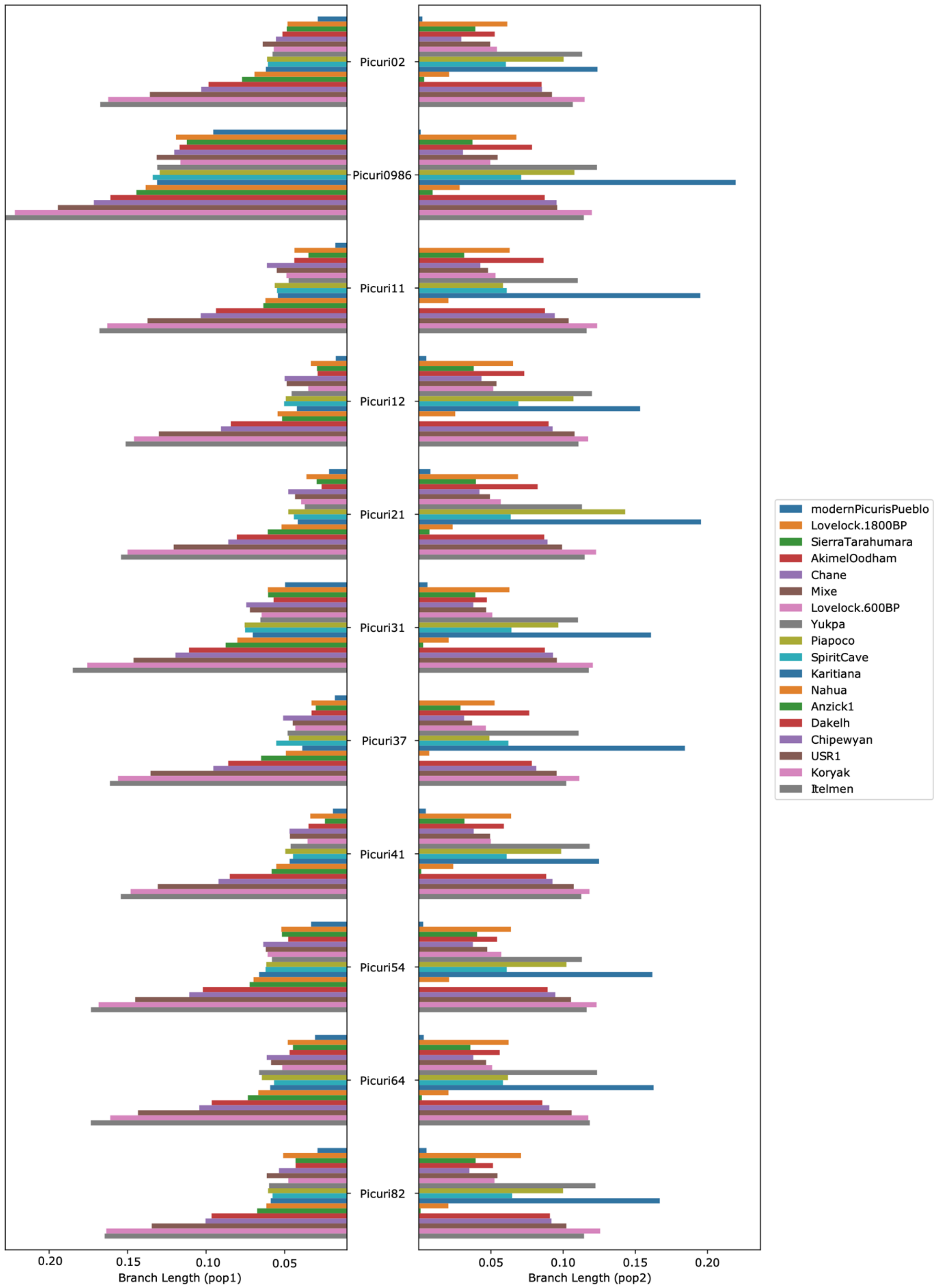

Supplemental Figure S43 Pairwise branch length estimated between ancient Picuris Pueblo individuals and a set of high-coverage genomes from Northeast Asia and the Americas. In all cases, the branch leading to ancient Picuris (left) is shorter when measured against present-day individuals from Picuris Pueblo.

References:

1. Claw, K. G. *et al.* Chaco Canyon Dig Unearths Ethical Concerns. *Hum. Biol.* **89**, 177 (2017).
2. Cortez, A. D., Bolnick, D. A., Nicholas, G., Bardill, J. & Colwell, C. An ethical crisis in ancient DNA research: Insights from the Chaco Canyon controversy as a case study. *J. Soc. Archaeol.* **21**, 157–178 (2021).
3. Mithun, M. *The Languages of Native North America*. (Cambridge University Press, 2001).
4. Lewis, A. C. F. *et al.* Getting genetic ancestry right for science and society. *Science* **376**, 250–252 (2022).
5. Adler, M. A. & Dick, H. W. *Picuris Pueblo through Time: Eight Centuries of Change in a Northern Rio Grande Pueblo*. (William P. Clements Center for Southwest Studies, Southern Methodist University, 1999).
6. Dick, H. W. *Picuris Pueblo Excavations, November 1964*. (Department of the Interior, National Park Service, Santa Fe. Document NO. PB 177047, National Technical Service Information, Springfield, VA, 1965).
7. Friedman, R. A., Sofaer, A. & Weiner, R. Remote Sensing of Chaco Roads Revisited: Lidar Documentation of the Great North Road, Pueblo Alto Landscape, and Aztec Airport Mesa Road. *Adv. Archaeol. Pract.* **5**, 363–381 (2017).
8. Crotty, H. K. Kiva Murals and Iconography at Picuris Pueblo. in *Picuris Pueblo Through Time: Eight Centuries of Change at a Northern Rio Grande Pueblo* 149–188 (Clements Center for Southwest Studies, Southern Methodist University, Dallas, TX, 1999).
9. Reimer, P. J. *et al.* The IntCal20 Northern Hemisphere Radiocarbon Age Calibration Curve (0–55 cal kBP). *Radiocarbon* **62**, 725–757 (2020).
10. Reed, P. F. *The Puebloan Society of Chaco Canyon*. (Greenwood Press, Westport, Conn., 2004).

11. Lekson, S. H., Windes, T. C., Stein, J. R. & Judge, W. J. The Chaco Canyon Community. *Sci. Am.* **259**, 100–109 (1988).
12. Allen, F. W. & Larsen, H. D. Heredity of Agglutinogens M and N Among Pueblo and Blackfeet Indians. *J. Immunol.* **32**, 301–305 (1937).
13. Gerheim, E. B. Incidence of Rh factor among the Indians of the Southwest. *Proc. Soc. Exp. Biol. Med.* **66**, 419–420 (1947).
14. Hanna, B. L., Dahlberg, A. A. & Strandskov, H. H. A preliminary study of the population history of the Pima Indians. *Am. J. Hum. Genet.* **5**, 377–388 (1953).
15. Brown, K. S., Hanna, B. L., Dahlberg, A. A. & Strandskov, H. H. The distribution of blood group alleles among Indians of Southwest North America. *Am. J. Hum. Genet.* **10**, 175–195 (1958).
16. Boyd, W. C. & Boyd, L. G. Blood Grouping by Means of Preserved Muscle. *Science* **78**, 578–578 (1933).
17. Boyd, W. C. & Boyd, L. G. Blood Grouping Tests on 300 Mummies. *J. Immunol.* **32**, 307–319 (1937).
18. Wyman, L. C. & Boyd, W. C. Blood Group Determinations of Prehistoric American Indians. *Am. Anthropol.* **39**, 583–592 (1937).
19. Pääbo, S. Molecular cloning of Ancient Egyptian mummy DNA. *Nature* **314**, 644–645 (1985).
20. Shearin, N. L., King, E. J. & O'Rourke, D. H. DNA preservation in Precolumbian remains from the American Southwest. *Hum. Evol.* **4**, 263–270 (1989).
21. Willerslev, E. & Cooper, A. Ancient DNA. *Proc. R. Soc. B Biol. Sci.* **272**, 3–16 (2004).
22. Cappellini, E. *et al.* Ancient Biomolecules and Evolutionary Inference. *Annu. Rev. Biochem.* **87**, 1029–1060 (2018).

23. Parr, R. L., Carlyle, S. W. & O'Rourke, D. H. Ancient DNA analysis of Fremont Amerindians of the Great Salt Lake Wetlands. *Am. J. Phys. Anthropol.* **99**, 507–518 (1996).

24. O'Rourke, D. H., Carlyle, S. W. & Parr, R. L. Ancient DNA: Methods, progress, and perspectives. *Am. J. Hum. Biol.* **8**, 557–571 (1996).

25. Carlyle, S. W., Parr, R. L., Hayes, M. G. & O'Rourke, D. H. Context of maternal lineages in the greater Southwest. *Am. J. Phys. Anthropol.* **113**, 85–101 (2000).

26. Kaestle, F. A. & Smith, D. G. Ancient mitochondrial DNA evidence for prehistoric population movement: The numic expansion. *Am. J. Phys. Anthropol.* **115**, 1–12 (2001).

27. Cann, R. L., Stoneking, M. & Wilson, A. C. Mitochondrial DNA and human evolution. *Nature* **325**, 31–36 (1987).

28. Schurr, T. G. *et al.* Amerindian mitochondrial DNAs have rare Asian mutations at high frequencies, suggesting they derived from four primary maternal lineages. *Am. J. Hum. Genet.* **46**, 613–623 (1990).

29. Lorenz, J. G. & Smith, D. G. Distribution of four founding mtDNA haplogroups among Native North Americans. *Am. J. Phys. Anthropol.* **101**, 307–323 (1996).

30. Malhi, R. S. *et al.* The Structure of Diversity within New World Mitochondrial DNA Haplogroups: Implications for the Prehistory of North America. *Am. J. Hum. Genet.* **70**, 905–919 (2002).

31. Malhi, R. S. *et al.* Native American mtDNA prehistory in the American Southwest. *Am. J. Phys. Anthropol.* **120**, 108–124 (2003).

32. Kemp, B. M. *et al.* Evaluating the Farming/Language Dispersal Hypothesis with genetic variation exhibited by populations in the Southwest and Mesoamerica. *Proc. Natl. Acad. Sci.* **107**, 6759–6764 (2010).

33. Bodner, M. *et al.* The Mitochondrial DNA Landscape of Modern Mexico. *Genes* **12**, 1453 (2021).

34. Jobling, M. A. & Tyler-Smith, C. Human Y-chromosome variation in the genome-sequencing era. *Nat. Rev. Genet.* **18**, 485–497 (2017).

35. Pinotti, T. *et al.* Y Chromosome Sequences Reveal a Short Beringian Standstill, Rapid Expansion, and early Population structure of Native American Founders. *Curr. Biol.* **29**, 149-157.e3 (2019).

36. Malhi, R. S. *et al.* Distribution of Y chromosomes among native North Americans: A study of Athapaskan population history. *Am. J. Phys. Anthropol.* **137**, 412–424 (2008).

37. Garrison, N. A. Genomic Justice for Native Americans: Impact of the Havasupai Case on Genetic Research. *Sci. Technol. Hum. Values* **38**, 201–223 (2013).

38. Bardill, J. *et al.* Advancing the ethics of paleogenomics. *Science* **360**, 384–385 (2018).

39. Garrison, N. A. *et al.* Genomic Research Through an Indigenous Lens: Understanding the Expectations. *Annu. Rev. Genomics Hum. Genet.* **20**, 495–517 (2019).

40. Tsosie, K. S. *et al.* Ancient-DNA researchers write their own rules. *Nature* **600**, 37–37 (2021).

41. Alpaslan-Roodenberg, S. *et al.* Ethics of DNA research on human remains: five globally applicable guidelines. *Nature* **599**, 41–46 (2021).

42. Ávila-Arcos, M. C., de la Fuente Castro, C., Nieves-Colón, M. A. & Raghavan, M. Recommendations for Sustainable Ancient DNA Research in the Global South: Voices From a New Generation of Paleogenomicists. *Front. Genet.* **13**, (2022).

43. Argüelles, J. M., Fuentes, A. & Yáñez, B. Analyzing asymmetries and praxis in aDNA research: A bioanthropological critique. *Am. Anthropol.* **124**, 130–140 (2022).

44. Kowal, E. *et al.* Community partnerships are fundamental to ethical ancient DNA research. *Hum. Genet. Genomics Adv.* 100161 (2023) doi:10.1016/j.xhgg.2022.100161.

45. Kennett, D. J. *et al.* Archaeogenomic evidence reveals prehistoric matrilineal dynasty. *Nat. Commun.* **8**, 14115 (2017).

46. Boaz, F. Evolution or Diffusion. *Am. Anthropol.* **26**, 340–344 (1924).

47. Steward, J. H. Ecological Aspects of Southwestern Society. *Anthropos* **32**, 87–104 (1937).

48. Ware, J. Kinship and Community in the Northern Southwest: Chaco and Beyond. *Am. Antiq.* **83**, 639–658 (2018).

49. Trager, G. L. Taos and Picuris: How Long Separated? *Int. J. Am. Linguist.* **35**, 180–182 (1969).

50. Leap, W. L. Who Were the Piro? *Anthropol. Linguist.* **13**, 321–330 (1971).

51. Harrington, J. P. Notes on the Piro Language. *Am. Anthropol.* **11**, 563–594 (1909).

52. Bartlett, J. R. The Language of the Piro. *Am. Anthropol.* **11**, 426–433 (1909).

53. Powell, J. W. *Indian Linguistic Families of America North of Mexico*. (Government Publishing Office, Washington, DC, 1891).

54. Harrington, J. P. *On Phonetic and Lexic Resemblances between Kiowa and Tanoan*. (Archaeological Institute of America, 1910).

55. Harrington, J. Vocabulary of the Kiowa Language. in *Bureau of American Ethnology Bulletin* vol. 84 (Smithsonian Institute, Washington, DC, 1928).

56. Miller, W. R. A Note on Kiowa Linguistic Affiliations. *Am. Anthropol.* **61**, 102–105 (1959).

57. Hale, K. Jemez and Kiowa Correspondences in Reference to Kiowa-Tanoan. *Int. J. Am. Linguist.* **28**, 1–5 (1962).

58. Hale, K. L. Toward a Reconstruction of Kiowa-Tanoan Phonology. *Int. J. Am. Linguist.* **33**, 112–120 (1967).
59. Davis, I. The Kiowa-Tanoan, Keresan, and Zuni Languages. in *The Languages of Native America* (eds. Campbell, L. & Mithun, M.) 390–443 (University of Texas Press, 1979). doi:10.7560/746244-009.
60. Spielmann, K. Coercion or cooperation? Plains-Pueblo interaction in the Protohistoric period. in *Farmers, Hunters, and Colonists: Interaction Between the Southwest and the Southern Plains* (ed. Spielmann, K.) 36–50 (University of Arizona Press, Tucson, 1991).
61. Greenhill, S. J. *et al.* A Recent Northern Origin for the Uto-Aztecan Family. *Language* **99**, (2023).
62. Gray, R. D. & Atkinson, Q. D. Language-tree divergence times support the Anatolian theory of Indo-European origin. *Nature* **426**, 435–439 (2003).
63. Bouckaert, R. *et al.* Mapping the Origins and Expansion of the Indo-European Language Family. *Science* **337**, 957–960 (2012).
64. Heggarty, P. *et al.* Language trees with sampled ancestors support a hybrid model for the origin of Indo-European languages. *Science* **381**, eabg0818 (2023).
65. Anthony, D. W. & Ringe, D. The Indo-European Homeland from Linguistic and Archaeological Perspectives. *Annu. Rev. Linguist.* **1**, 199–219 (2015).
66. Ortman, S. G. & McNeil, L. D. The Kiowa Odyssey: Evidence of historical relationships among Pueblo, Fremont, and Northwest Plains peoples. *Plains Anthropol.* **63**, 152–174 (2018).
67. Schillaci, M. A., Sutton, L. D., Wichmann, S. & López-Torres, S. Linguistic Clues to Kiowa-Tanoan Prehistory. *J. Southwest* **63**, 255–368 (2021).

68. Schillaci, M. A. The Development of Population Diversity at Chaco Canyon. *KIVA* **68**, 221–245 (2003).

69. Schillaci, M. A., Lakatos, S. A., Ferguson, J. R. & Wilson, C. D. Analysis of Ceramic Compositional Data from Late Developmental Period Sites in the Tewa Basin, New Mexico. *KIVA* **86**, 70–107 (2020).

70. Fowles, S. The Pueblo Village in an Age of Reformation (AD 1300–1600). in *The Oxford Handbook of North American Archaeology* (ed. Pauketat, T. R.) 0 (Oxford University Press, 2012). doi:10.1093/oxfordhb/9780195380118.013.0052.

71. Cooper, Z. J. The Initial Farming Population of the Northern Rio Grande: A Multidisciplinary Analysis. *J. Anthropol. Res.* **76**, 439–479 (2020).

72. Ortman, S. G. *Winds from the North: Tewa Origins and Historical Anthropology*. (University of Utah Press, 2012). doi:10.1353/book41479.

73. Damgaard, P. B. *et al.* Improving access to endogenous DNA in ancient bones and teeth. *Sci. Rep.* **5**, 11184 (2015).

74. Hansen, H. B. *et al.* Comparing Ancient DNA Preservation in Petrous Bone and Tooth Cementum. *PLOS ONE* **12**, e0170940 (2017).

75. Rohland, N., Glocke, I., Aximu-Petri, A. & Meyer, M. Extraction of highly degraded DNA from ancient bones, teeth and sediments for high-throughput sequencing. *Nat. Protoc.* **13**, 2447–2461 (2018).

76. Allentoft, M. E. *et al.* Population genomics of Bronze Age Eurasia. *Nature* **522**, 167–172 (2015).

77. Meyer, M. & Kircher, M. Illumina Sequencing Library Preparation for Highly Multiplexed Target Capture and Sequencing. *Cold Spring Harb. Protoc.* **2010**, pdb.prot5448 (2010).

78. Margaryan, A. *et al.* Population genomics of the Viking world. *Nature* **585**, 390–396 (2020).
79. Briggs, A. W. *et al.* Patterns of damage in genomic DNA sequences from a Neandertal. *Proc. Natl. Acad. Sci.* **104**, 14616–14621 (2007).
80. Briggs, A. W. *et al.* Removal of deaminated cytosines and detection of in vivo methylation in ancient DNA. *Nucleic Acids Res.* **38**, e87 (2010).
81. Gansauge, M.-T. & Meyer, M. Single-stranded DNA library preparation for the sequencing of ancient or damaged DNA. *Nat. Protoc.* **8**, 737–748 (2013).
82. Gansauge, M.-T. *et al.* Single-stranded DNA library preparation from highly degraded DNA using T4 DNA ligase. *Nucleic Acids Res.* **45**, e79 (2017).
83. Gansauge, M.-T., Aximu-Petri, A., Nagel, S. & Meyer, M. Manual and automated preparation of single-stranded DNA libraries for the sequencing of DNA from ancient biological remains and other sources of highly degraded DNA. *Nat. Protoc.* **15**, 2279–2300 (2020).
84. Schubert, M., Lindgreen, S. & Orlando, L. AdapterRemoval v2: rapid adapter trimming, identification, and read merging. *BMC Res. Notes* **9**, 88 (2016).
85. Li, H. & Durbin, R. Fast and accurate short read alignment with Burrows-Wheeler transform. *Bioinformatics* **25**, 1754–1760 (2009).
86. Li, H. *et al.* The Sequence Alignment/Map format and SAMtools. *Bioinformatics* **25**, 2078–2079 (2009).
87. Danecek, P. *et al.* Twelve years of SAMtools and BCFtools. *GigaScience* **10**, giab008 (2021).
88. Poznik, G. D. *et al.* Sequencing Y Chromosomes Resolves Discrepancy in Time to Common Ancestor of Males versus Females. *Science* **341**, 562–565 (2013).

89. Jónsson, H., Ginolhac, A., Schubert, M., Johnson, P. L. F. & Orlando, L. mapDamage2.0: fast approximate Bayesian estimates of ancient DNA damage parameters. *Bioinforma. Oxf. Engl.* **29**, 1682–1684 (2013).

90. Renaud, G., Slon, V., Duggan, A. T. & Kelso, J. Schmutzi: estimation of contamination and endogenous mitochondrial consensus calling for ancient DNA. *Genome Biol.* **16**, 224 (2015).

91. Fu, Q. *et al.* A Revised Timescale for Human Evolution Based on Ancient Mitochondrial Genomes. *Curr. Biol.* **23**, 553–559 (2013).

92. Rasmussen, M. *et al.* An Aboriginal Australian Genome Reveals Separate Human Dispersals into Asia. *Science* **334**, 94–98 (2011).

93. Korneliussen, T. S., Albrechtsen, A. & Nielsen, R. ANGSD: Analysis of Next Generation Sequencing Data. *BMC Bioinformatics* **15**, 356 (2014).

94. Moreno-Mayar, J. V. *et al.* A likelihood method for estimating present-day human contamination in ancient male samples using low-depth X-chromosome data. *Bioinformatics* **36**, 828–841 (2020).

95. Gower, G. *et al.* Widespread male sex bias in mammal fossil and museum collections. *Proc. Natl. Acad. Sci.* **116**, 19019–19024 (2019).

96. Weissensteiner, H. *et al.* HaploGrep 2: mitochondrial haplogroup classification in the era of high-throughput sequencing. *Nucleic Acids Res.* **44**, W58–W63 (2016).

97. Ingman, M., Kaessmann, H., Pääbo, S. & Gyllensten, U. Mitochondrial genome variation and the origin of modern humans. *Nature* **408**, 708–713 (2000).

98. Herrnstadt, C. *et al.* Reduced-Median-Network Analysis of Complete Mitochondrial DNA Coding-Region Sequences for the Major African, Asian, and European Haplogroups. *Am. J. Hum. Genet.* **70**, 1152–1171 (2002).

99. Kong, Q.-P. *et al.* Phylogeny of East Asian Mitochondrial DNA Lineages Inferred from Complete Sequences. *Am. J. Hum. Genet.* **73**, 671–676 (2003).

100. Achilli, A. *et al.* The Phylogeny of the Four Pan-American MtDNA Haplogroups: Implications for Evolutionary and Disease Studies. *PLOS ONE* **3**, e1764 (2008).

101. Fagundes, N. J. R. *et al.* Mitochondrial Population Genomics Supports a Single Pre-Clovis Origin with a Coastal Route for the Peopling of the Americas. *Am. J. Hum. Genet.* **82**, 583–592 (2008).

102. Just, R. S., Diegoli, T. M., Saunier, J. L., Irwin, J. A. & Parsons, T. J. Complete mitochondrial genome sequences for 265 African American and U.S. “Hispanic” individuals. *Forensic Sci. Int. Genet.* **2**, e45–e48 (2008).

103. Derenko, M. *et al.* Origin and Post-Glacial Dispersal of Mitochondrial DNA Haplogroups C and D in Northern Asia. *PLOS ONE* **5**, e15214 (2010).

104. Kong, Q.-P. *et al.* Large-Scale mtDNA Screening Reveals a Surprising Matrilineal Complexity in East Asia and Its Implications to the Peopling of the Region. *Mol. Biol. Evol.* **28**, 513–522 (2011).

105. Kumar, S. *et al.* Large scale mitochondrial sequencing in Mexican Americans suggests a reappraisal of Native American origins. *BMC Evol. Biol.* **11**, 293 (2011).

106. Pope, A. M., Carr, S. M., Smith, K. N. & Marshall, H. D. Mitogenomic and microsatellite variation in descendants of the founder population of Newfoundland: high genetic diversity in an historically isolated population. *Genome* **54**, 110–119 (2011).

107. Behar, D. M. *et al.* A “Copernican” Reassessment of the Human Mitochondrial DNA Tree from its Root. *Am. J. Hum. Genet.* **90**, 675–684 (2012).

108. Achilli, A. *et al.* Reconciling migration models to the Americas with the variation of North American native mitogenomes. *Proc. Natl. Acad. Sci.* **110**, 14308–14313 (2013).

109. Sevini, F. *et al.* Analysis of Population Substructure in Two Sympatric Populations of Gran Chaco, Argentina. *PLOS ONE* **8**, e64054 (2013).

110. Just, R. S. *et al.* Full mtGenome reference data: Development and characterization of 588 forensic-quality haplotypes representing three U.S. populations. *Forensic Sci. Int. Genet.* **14**, 141–155 (2015).

111. Poznik, G. D. *et al.* Punctuated bursts in human male demography inferred from 1,244 worldwide Y-chromosome sequences. *Nat. Genet.* **48**, 593–599 (2016).

112. Llamas, B. *et al.* Ancient mitochondrial DNA provides high-resolution time scale of the peopling of the Americas. *Sci. Adv.* **2**, e1501385 (2016).

113. Morales-Arce, A. Y. *et al.* Successful reconstruction of whole mitochondrial genomes from ancient Central America and Mexico. *Sci. Rep.* **7**, 18100 (2017).

114. Moreno-Mayar, J. V. *et al.* Early human dispersals within the Americas. *Science* **362**, eaav2621 (2018).

115. Scheib, C. L. *et al.* Ancient human parallel lineages within North America contributed to a coastal expansion. *Science* **360**, 1024–1027 (2018).

116. Wood, M. R. *et al.* Resolving mitochondrial haplogroups B2 and B4 with next-generation mitogenome sequencing to distinguish Native American from Asian haplotypes. *Forensic Sci. Int. Genet.* **43**, 102143 (2019).

117. Bergström, A. *et al.* Insights into human genetic variation and population history from 929 diverse genomes. *Science* (2020) doi:10.1126/science.aay5012.

118. Taylor, C. R. *et al.* Platinum-Quality Mitogenome Haplotypes from United States Populations. *Genes* **11**, 1290 (2020).

119. Katoh, K. & Standley, D. M. MAFFT Multiple Sequence Alignment Software Version 7: Improvements in Performance and Usability. *Mol. Biol. Evol.* **30**, 772–780 (2013).

120. Quinlan, A. R. & Hall, I. M. BEDTools: a flexible suite of utilities for comparing genomic features. *Bioinformatics* **26**, 841–842 (2010).

121. van Oven, M. PhyloTree Build 17: Growing the human mitochondrial DNA tree. *Forensic Sci. Int. Genet. Suppl. Ser.* **5**, e392–e394 (2015).

122. Kozlov, A. M., Darriba, D., Flouri, T., Morel, B. & Stamatakis, A. RAxML-NG: a fast, scalable and user-friendly tool for maximum likelihood phylogenetic inference. *Bioinformatics* **35**, 4453–4455 (2019).

123. Pattengale, N. D., Alipour, M., Bininda-Emonds, O. R. P., Moret, B. M. E. & Stamatakis, A. How Many Bootstrap Replicates Are Necessary? *J. Comput. Biol.* **17**, 337–354 (2010).

124. Felsenstein, J. Confidence Limits on Phylogenies: an Approach Using the Bootstrap. *Evolution* **39**, 783–791 (1985).

125. Bouckaert, R. *et al.* BEAST 2: A Software Platform for Bayesian Evolutionary Analysis. *PLOS Comput. Biol.* **10**, e1003537 (2014).

126. Begay, R. L. *et al.* Weaving the Strands of Life (Iiná Bitł'ool): History of Genetic Research Involving Navajo People. *Hum. Biol.* **91**, 189–208 (2020).

127. Danecek, P. *et al.* The variant call format and VCFtools. *Bioinformatics* **27**, 2156–2158 (2011).

128. Allentoft, M. E. *et al.* Population Genomics of Stone Age Eurasia. 2022.05.04.490594 Preprint at https://doi.org/10.1101/2022.05.04.490594 (2022).

129. Colombo, G. *et al.* Overview of the Americas' First Peopling from a Patrilineal Perspective: New Evidence from the Southern Continent. *Genes* **13**, 220 (2022).

130. Adhikari, K., Mendoza-Revilla, J., Chacón-Duque, J. C., Fuentes-Guajardo, M. & Ruiz-Linares, A. Admixture in Latin America. *Curr. Opin. Genet. Dev.* **41**, 106–114 (2016).

131. Raghavan, M. *et al.* Genomic evidence for the Pleistocene and recent population history of Native Americans. *Science* **349**, aab3884 (2015).
132. Schroeder, H. *et al.* Origins and genetic legacies of the Caribbean Taino. *Proc. Natl. Acad. Sci.* **115**, 2341–2346 (2018).
133. Popović, D. *et al.* Ancient genomes reveal long-range influence of the pre-Columbian culture and site of Tiwanaku. *Sci. Adv.* **7**, eabg7261 (2021).
134. Campelo dos Santos, A. L. *et al.* Genomic evidence for ancient human migration routes along South America's Atlantic coast. *Proc. R. Soc. B Biol. Sci.* **289**, 20221078 (2022).
135. de la Fuente, C. *et al.* Genomic insights into the origin and diversification of late maritime hunter-gatherers from the Chilean Patagonia. *Proc. Natl. Acad. Sci.* **115**, E4006–E4012 (2018).
136. Rasmussen, M. *et al.* Ancient human genome sequence of an extinct Palaeo-Eskimo. *Nature* **463**, 757–762 (2010).
137. Villa-Islas, V. *et al.* Demographic history and genetic structure in pre-Hispanic Central Mexico. *Science* **380**, eadd6142 (2023).
138. Capodiferro, M. R. *et al.* Archaeogenomic distinctiveness of the Isthmo-Colombian area. *Cell* **184**, 1706-1723.e24 (2021).
139. Bongers, J. L. *et al.* Integration of ancient DNA with transdisciplinary dataset finds strong support for Inca resettlement in the south Peruvian coast. *Proc. Natl. Acad. Sci.* **117**, 18359–18368 (2020).
140. Lindo, J. *et al.* The genetic prehistory of the Andean highlands 7000 years BP though European contact. *Sci. Adv.* **4**, eaau4921 (2018).
141. Raghavan, M. *et al.* Upper Palaeolithic Siberian genome reveals dual ancestry of Native Americans. *Nature* **505**, 87–91 (2014).

142. Sikora, M. *et al.* The population history of northeastern Siberia since the Pleistocene. *Nature* **570**, 182–188 (2019).

143. Flegontov, P. *et al.* Palaeo-Eskimo genetic ancestry and the peopling of Chukotka and North America. *Nature* **570**, 236–240 (2019).

144. Moreno-Mayar, J. V. *et al.* Terminal Pleistocene Alaskan genome reveals first founding population of Native Americans. *Nature* **553**, 203–207 (2018).

145. Rasmussen, M. *et al.* The genome of a Late Pleistocene human from a Clovis burial site in western Montana. *Nature* **506**, 225–229 (2014).

146. Rasmussen, M. *et al.* The ancestry and affiliations of Kennewick Man. *Nature* **523**, 455–458 (2015).

147. Mallick, S. *et al.* The Simons Genome Diversity Project: 300 genomes from 142 diverse populations. *Nature* **538**, 201–206 (2016).

148. Raghavan, M. *et al.* The genetic prehistory of the New World Arctic. *Science* **345**, 1255832 (2014).

149. Joseph, S. K. *et al.* Genomic evidence for adaptation to tuberculosis in the Andes before European contact. *iScience* **26**, 106034 (2023).

150. Mota, B. S. da *et al.* Imputation of ancient genomes. 2022.07.19.500636 Preprint at https://doi.org/10.1101/2022.07.19.500636 (2022).

151. Auton, A. *et al.* A global reference for human genetic variation. *Nature* **526**, 68–74 (2015).

152. Rubinacci, S., Ribeiro, D. M., Hofmeister, R. J. & Delaneau, O. Efficient phasing and imputation of low-coverage sequencing data using large reference panels. *Nat. Genet.* **53**, 120–126 (2021).

153. Posth, C. *et al.* Reconstructing the Deep Population History of Central and South America. *Cell* **175**, 1185-1197.e22 (2018).

154. Nägele, K. *et al.* Genomic insights into the early peopling of the Caribbean. *Science* **369**, 456–460 (2020).

155. Nakatsuka, N. *et al.* A Paleogenomic Reconstruction of the Deep Population History of the Andes. *Cell* **181**, 1131-1145.e21 (2020).

156. Nakatsuka, N. *et al.* Ancient genomes in South Patagonia reveal population movements associated with technological shifts and geography. *Nat. Commun.* **11**, 3868 (2020).

157. Fernandes, D. M. *et al.* A genetic history of the pre-contact Caribbean. *Nature* **590**, 103–110 (2021).

158. Tiesler, V. *et al.* Life and death in early colonial Campeche: new insights from ancient DNA. *Antiquity* **96**, 937–954 (2022).

159. Kennett, D. J. *et al.* South-to-north migration preceded the advent of intensive farming in the Maya region. *Nat. Commun.* **13**, 1530 (2022).

160. Nakatsuka, N. *et al.* Genetic continuity and change among the Indigenous peoples of California. *Nature* **624**, 122–129 (2023).

161. Pritchard, J. K., Stephens, M. & Donnelly, P. Inference of Population Structure Using Multilocus Genotype Data. *Genetics* **155**, 945–959 (2000).

162. Alexander, D. H., Novembre, J. & Lange, K. Fast model-based estimation of ancestry in unrelated individuals. *Genome Res.* **19**, 1655–1664 (2009).

163. Maples, B. K., Gravel, S., Kenny, E. E. & Bustamante, C. D. RFMix: A Discriminative Modeling Approach for Rapid and Robust Local-Ancestry Inference. *Am. J. Hum. Genet.* **93**, 278–288 (2013).

164. Reich, D. *et al.* Reconstructing Native American population history. *Nature* **488**, 370–374 (2012).

165. Verdu, P. *et al.* Patterns of Admixture and Population Structure in Native Populations of Northwest North America. *PLOS Genet.* **10**, e1004530 (2014).

166. Gnecchi-Ruscone, G. A. *et al.* Dissecting the Pre-Columbian Genomic Ancestry of Native Americans along the Andes–Amazonia Divide. *Mol. Biol. Evol.* **36**, 1254–1269 (2019).

167. Borda, V. *et al.* The genetic structure and adaptation of Andean highlanders and Amazonians are influenced by the interplay between geography and culture. *Proc. Natl. Acad. Sci.* **117**, 32557–32565 (2020).

168. Purcell, S. *et al.* PLINK: A Tool Set for Whole-Genome Association and Population-Based Linkage Analyses. *Am. J. Hum. Genet.* **81**, 559–575 (2007).

169. Patterson, N., Price, A. L. & Reich, D. Population Structure and Eigenanalysis. *PLoS Genet.* **2**, e190 (2006).

170. Patterson, N. *et al.* Ancient Admixture in Human History. *Genetics* **192**, 1065–1093 (2012).

171. Moreno-Mayar, J. V. FrAnTK: a Frequency-based Analysis ToolKit for efficient exploration of allele sharing patterns in present-day and ancient genomic datasets. *G3 GenesGenomesGenetics* jkab357 (2021) doi:10.1093/g3journal/jkab357.

172. Pickrell, J. K. & Pritchard, J. K. Inference of Population Splits and Mixtures from Genome-Wide Allele Frequency Data. *PLOS Genet.* **8**, e1002967 (2012).

173. Willerslev, E. & Meltzer, D. J. Peopling of the Americas as inferred from ancient genomics. *Nature* **594**, 356–364 (2021).

174. Seymour, D. J. Gateways for Athabascan Migration to the American Southwest. *Plains Anthropol.* **57**, 149–161 (2012).

175. Dulik, M. C. *et al.* Y-chromosome analysis reveals genetic divergence and new founding native lineages in Athapaskan- and Eskimoan-speaking populations. *Proc. Natl. Acad. Sci.* **109**, 8471–8476 (2012).

176. Nielsen, S. V. *et al.* Bayesian inference of admixture graphs on Native American and Arctic populations. 63 (2022).

177. Harney, É., Patterson, N., Reich, D. & Wakeley, J. Assessing the performance of qpAdm: a statistical tool for studying population admixture. *Genetics* **217**, iyaa045 (2021).

178. Tournebize, R., Chu, G. & Moorjani, P. Reconstructing the history of founder events using genome-wide patterns of allele sharing across individuals. *PLOS Genet.* **18**, e1010243 (2022).

179. Chintalapati, M., Patterson, N. & Moorjani, P. The spatiotemporal patterns of major human admixture events during the European Holocene. *eLife* **11**, e77625 (2022).

180. Browning, B. L. & Browning, S. R. Detecting Identity by Descent and Estimating Genotype Error Rates in Sequence Data. *Am. J. Hum. Genet.* **93**, 840–851 (2013).

181. Moreno-Estrada, A. *et al.* Reconstructing the Population Genetic History of the Caribbean. *PLOS Genet.* **9**, e1003925 (2013).

182. Browning, S. R. & Browning, B. L. Accurate Non-parametric Estimation of Recent Effective Population Size from Segments of Identity by Descent. *Am. J. Hum. Genet.* **97**, 404–418 (2015).

183. Fournier, R., Tsangalidou, Z., Reich, D. & Palamara, P. F. Haplotype-based inference of recent effective population size in modern and ancient DNA samples. *Nat. Commun.* **14**, 7945 (2023).

184. Castro e Silva, M. A. *et al.* Population Histories and Genomic Diversity of South American Natives. *Mol. Biol. Evol.* msab339 (2021) doi:10.1093/molbev/msab339.
