# Supplementary material for: Picuris Pueblo oral history and genomics reveal continuity in US Southwest
Source: Nature. 2025 Apr 30;642(8066):125–32. doi: 10.1038/s41586-025-08791-9 (PMC12137115; doi:10.1038/s41586-025-08791-9)
Supplement: Supplementary file 2 — Reporting Summary [file 41586_2025_8791_MOESM2_ESM.pdf]

nature portfolio

Corresponding author(s): Craig Quanchello; Eske Willerslev

Last updated by author(s): 05/02/2025

Reporting Summary

Nature Portfolio wishes to improve the reproducibility of the work that we publish. This form provides structure for consistency and transparency in reporting. For further information on Nature Portfolio policies, see our Editorial Policies and the Editorial Policy Checklist.

Statistics

For all statistical analyses, confirm that the following items are present in the figure legend, table legend, main text, or Methods section.

n/a	Confirmed	
☐	☒	The exact sample size (*n*) for each experimental group/condition, given as a discrete number and unit of measurement
☐	☒	A statement on whether measurements were taken from distinct samples or whether the same sample was measured repeatedly
☐	☒	The statistical test(s) used AND whether they are one- or two-sided<br>*Only common tests should be described solely by name; describe more complex techniques in the Methods section.*
☐	☒	A description of all covariates tested
☐	☒	A description of any assumptions or corrections, such as tests of normality and adjustment for multiple comparisons
☐	☒	A full description of the statistical parameters including central tendency (e.g. means) or other basic estimates (e.g. regression coefficient) AND variation (e.g. standard deviation) or associated estimates of uncertainty (e.g. confidence intervals)
☐	☒	For null hypothesis testing, the test statistic (e.g. *F*, *t*, *r*) with confidence intervals, effect sizes, degrees of freedom and *P* value noted<br>*Give P values as exact values whenever suitable.*
☐	☒	For Bayesian analysis, information on the choice of priors and Markov chain Monte Carlo settings
☐	☒	For hierarchical and complex designs, identification of the appropriate level for tests and full reporting of outcomes
☒	☐	Estimates of effect sizes (e.g. Cohen's *d*, Pearson's *r*), indicating how they were calculated

Our web collection on statistics for biologists contains articles on many of the points above.

Software and code

Policy information about availability of computer code

Data collection	No software was used for data collection.
Data analysis	AdapterRemoval (v.2.3.2)<br>BWA (v.0.7-17)<br>Picard (v.2.25.0)<br>pysam v0.221<br>mapDamage (v2.2.1)<br>bam2prof (v1.0)<br>ContamMix (v1.0)<br>ANGSD (v.0931)<br>samtools (v.1.3.1)<br>Haplogrep (v.3)<br>mafft (v7.490)<br>raxml-ng (v.0.8.1)<br>BEAST (v.2.6)<br>fastq-pair (v0.3)<br>bcftools (v.1.17)<br>GLIMPSE (v.1.1.1)<br>KING (v2.3.0)<br>ADMIXTURE (v.1.3.0)<br>RFMIX (v2.03-r0)

bedtools (v.2.31.0)
plink (v.1.90)
FrAnTK (6d61ab8)
Treemix (v1.12)
ADMIXTOOLS2 (v2.0.0)
ASCEND (v.10.1.1)
DATES (v4010)
IBDseq (r1206)
GenomicRanges (v3.15)
IBDNe (04Sep15.e78)
HapNe-LD (a7f0c66)
OxCal (v.4.4)
ESRI ArcGIS Pro 3.0
Cartopy (v0.24.1)

For manuscripts utilizing custom algorithms or software that are central to the research but not yet described in published literature, software must be made available to editors and reviewers. We strongly encourage code deposition in a community repository (e.g. GitHub). See the Nature Portfolio guidelines for submitting code & software for further information.

Data

Policy information about availability of data

All manuscripts must include a data availability statement. This statement should provide the following information, where applicable:

- Accession codes, unique identifiers, or web links for publicly available datasets
- A description of any restrictions on data availability
- For clinical datasets or third party data, please ensure that the statement adheres to our policy

AAll maps were made using freely available vector and raster data from Natural Earth or the US Geological Survey and plotted using ESRI ArcGIS Pro 3.0 and cartopy 0.24.1. Compressed sequence alignment map files (BAM) aligned using human reference genome GRCh38 for ancient and present-day individuals are available in the European Genome-Phenome Archive under accession number EGAD50000001245 and EGAD50000001246. Picuris Pueblo is the sole owner of this dataset. Data access is controlled by the Picuris Pueblo Tribal Council through standard signed agreements that regulate the use of the ancient and/or present-day data. Consent for access will be granted exclusively to universities and research institutions exclusively for studies in population genetics that focus on understanding population history. The agreement strictly prohibits the use of the data for commercial purposes, inclusion in any kind of private databases (including forensic, mitochondrial or Y-chromosome) or medical or natural selection studies. Data cannot be used for tribal enrollment purposes. Present-day Picuris genomes are available exclusively for replication purposes using exactly identical comparison datasets, parameters and software and are controlled under a different agreement. Requests for data access will be evaluated jointly by the authors and the currently serving Picuris Pueblo Tribal Council every three months during the first year since publication, and every six months thereafter. Legitimate medical applications will be considered by the Picuris Pueblo Tribal Council if they explicitly serve Picuris health interests and guarantee that Picuris Pueblo retains full ownership of the data at all times and the right to halt any project at its sole discretion. Additionally, previously published genomic data used for comparison are detailed and referenced in Supplementary Tables S4-S6. They were obtained from the following sources:
• access granted by authors
• custom repositories (https://ftp.1000genomes.ebi.ac.uk/vol1/ftp/data_collections/)
• European Nucleotide Archive: SRX3810325, SRS937956, PRJEB203987, SRP09496510, PRJEB973359, PRJEB2039867, PRJEB2544568, PRJEB2896169, PRJEB6631971, PRJEB5144072, PRJEB2462993, PRJEB2257894, PRJEB4237295, PRJEB41550111, PRJNA883375112, SRA010102113, PRJEB37726114, PRJNA470966115, SRP029640116, PRJEB29700 and PRJEB26336117, PRJEB30575118, PRJNA883976122, PRJEB39010124, PRJEB38555125, PRJEB37518126, PRJEB50901127, PRJEB37446128 and PRJEB49391129.

Research involving human participants, their data, or biological material

Policy information about studies with human participants or human data. See also policy information about sex, gender (identity/presentation), and sexual orientation and race, ethnicity and racism.

Reporting on sex and gender	No gender or sex information of the participants was disclosed to researchers.
Reporting on race, ethnicity, or other socially relevant groupings	No race, ethnicity or other socially relevant groupings were used as variables in the manuscript.
Population characteristics	Participants are enrolled members of Picuris Pueblo Tribal Nation. No other information was disclosed to researchers.
Recruitment	Participation was voluntary, and individuals were informed on the nature of the study by the Tribal liaison in both English and Northern Tiwa. Volunteers contributed with their biological sample after signing an informed consent form.
Ethics oversight	Picuris Pueblo Tribal Council and National Committee on Health Research Ethics in Denmark

Note that full information on the approval of the study protocol must also be provided in the manuscript.

Field-specific reporting

Please select the one below that is the best fit for your research. If you are not sure, read the appropriate sections before making your selection.

☐ Life sciences ☐ Behavioural & social sciences ☒ Ecological, evolutionary & environmental sciences

For a reference copy of the document with all sections, see nature.com/documents/nr-reporting-summary-flat.pdf

Ecological, evolutionary & environmental sciences study design

All studies must disclose on these points even when the disclosure is negative.

Study description	Sequence data from 16 ancient and 13 present-day individuals from Picuris Pueblo, Southwest USA was generated by initiative of the Tribal Nation. It was combined with data previously generated from the American continent and worldwide, to better understand Tribal history and establish links with other populations in the past.
Research sample	Ancient human remains (bone and dental cementum) and present-day saliva from Picuris Pueblo.
Sampling strategy	Samples were evaluated for macroscopic criteria known to correlate with ancient DNA preservation. Present-day samples were collected from volunteering participants.
Data collection	Sampled human remains were petrous bones, teeth and long bones (n=28). All lab work was conducted in a dedicated ancient DNA clean lab in the Centre for GeoGenetics, GLOBE Institute, University of Copenhagen.
Timing and spatial scale	Chronology is based on archaeological evidence and radiocarbon dating, and spans 900 CE to present-day.
Data exclusions	Screened samples with low endogenous DNA content (n=6) were not further processed nor included in subsequent analyses.
Reproducibility	Data quality and uncertainty was accounted for in computation analyses, and reported whenever applicable. Methods are published and freely available, and all data is available for replication purposes
Randomization	Groups were defined to represent temporal, geographical and/or genetic grouping
Blinding	Blinding was not applicable to the study.

Did the study involve field work? ☐ Yes ☒ No

Reporting for specific materials, systems and methods

We require information from authors about some types of materials, experimental systems and methods used in many studies. Here, indicate whether each material, system or method listed is relevant to your study. If you are not sure if a list item applies to your research, read the appropriate section before selecting a response.

Materials & experimental systems

n/a	Involved in the study	
☒	☐	Antibodies
☒	☐	Eukaryotic cell lines
☐	☒	Palaeontology and archaeology
☒	☐	Animals and other organisms
☒	☐	Clinical data
☒	☐	Dual use research of concern
☒	☐	Plants

Methods

n/a	Involved in the study	
☒	☐	ChIP-seq
☒	☐	Flow cytometry
☒	☐	MRI-based neuroimaging

Palaeontology and Archaeology

Specimen provenance	Ancient individuals were excavated in the early 1960s by Herbert Dick in Picuris Pueblo and in long term loan with Southern Methodist University. After two years consultation with the community, 28 samples of 22 ancient individuals were sent to Copenhagen for DNA and radiocarbon analyses.
Specimen deposition	Following the conclusion of this study, ancient individuals (including bone powder, digests, extracts, libraries and pools) will be returned and reburied in Picuris Pueblo, and will not be available for other kinds of research.
Dating methods	Two new radiocarbon dates were generated at the Keck-CCAMS Group, Irvine, California, USA.

☒ Tick this box to confirm that the raw and calibrated dates are available in the paper or in Supplementary Information.

Ethics oversight	Picuris Pueblo Tribal Nation, Southern Methodist University and University of Copenhagen.

Note that full information on the approval of the study protocol must also be provided in the manuscript.

Plants

Seed stocks	*Report on the source of all seed stocks or other plant material used. If applicable, state the seed stock centre and catalogue number. If plant specimens were collected from the field, describe the collection location, date and sampling procedures.*
Novel plant genotypes	*Describe the methods by which all novel plant genotypes were produced. This includes those generated by transgenic approaches, gene editing, chemical/radiation-based mutagenesis and hybridization. For transgenic lines, describe the transformation method, the number of independent lines analyzed and the generation upon which experiments were performed. For gene-edited lines, describe the editor used, the endogenous sequence targeted for editing, the targeting guide RNA sequence (if applicable) and how the editor was applied.*
Authentication	*Describe any authentication procedures for each seed stock used or novel genotype generated. Describe any experiments used to assess the effect of a mutation and, where applicable, how potential secondary effects (e.g. second site T-DNA insertions, mosiacism, off-target gene editing) were examined.*
